# Molecularly Engineered Phenoxazinone‐Skeleton Cascade‐Activated NIR Probes for Monitoring Fe^2+^/Viscosity in Ferroptosis‐Mediated Parkinson's Disease

**DOI:** 10.1002/advs.202524057

**Published:** 2026-03-01

**Authors:** Lixia Guo, Tao Liu, Hongfei Wang, Qi Gao, Zhaobo Yang, Zumeng Wang, Chenlong Li, Yubing Kang, Kahleong Lim, Wen Liu, Li Lu, Chengwu Zhang

**Affiliations:** ^1^ Third Hospital of Shanxi Medical University Shanxi Bethune Hospital Taiyuan P. R. China; ^2^ School of Basic Medical Sciences Shanxi Medical University Taiyuan P. R. China; ^3^ Department of Chemical and Materials Engineering Lyuliang University Lvliang P. R. China; ^4^ Lee Kong Chian School of Medicine Nanyang Technological University Singapore Singapore

**Keywords:** Parkinson's disease, cascade‐activated probes, ferroptosis, ferrous ion, viscosity, quercetin

## Abstract

Parkinson's disease (PD) is the second most common neurodegenerative disease, in which ferroptosis may be the crucial event leading to dopaminergic neuron death. Accumulated ferrous ions (Fe^2+^) and increased intracellular viscosity promote of ferroptosis. Precisely monitoring Fe^2+^/Viscosity, especially in ferroptosis, is crucial for PD theranostic. However, a feasible strategy is lacking. In this study, series of Fe^2+^/Viscosity cascade‐activated near‐infrared fluorescence probes (NP1–5) are synthesized. Through optical characterization and theoretical calculations, NP3 is selected as the optimal probe to monitor Fe^2^^+^/Viscosity via redox reactions and twisted intramolecular charge transfer processes. To verify this concept in the context of ferroptosis intervention in PD, an innovative nanoplatform is constructed based on NP3 and quercetin, modified with rabies virus glycoprotein‐29 and defined as PQR nanoparticles (PQR NPs). In vitro and in vivo experiments demonstrate that PQR NPs not only alleviate ferroptosis‐induced loss of dopaminergic neurons by reducing oxidative stress and neuroinflammation, mitigating α‐synuclein aggregation, and restoring mitochondrial function, but also could monitor the elevated Fe^2^^+^/Viscosity in ferroptosis of PD models. Present study developed a facile tool for monitoring Fe^2+^/Viscosity in ferroptosis. The findings have strong application potential in theranostics of PD and other ferroptosis related diseases.

## Graphical abstract

A series of Fe^2^^+^/viscosity cascade‐activated NIR fluorescence probes (NP1–5) are synthesized, and NP3 is selected for its optimal properties. To verify application of NP3 in ferroptosis intervention in PD, PQR NPs, is constructed by NP3 and quercetin self‐assembling. The result demonstrate that PQR NPs alleviate ferroptosis‐induced dopaminergic neuron loss and could monitoring Fe^2^^+^/viscosity fluctuation in ferroptosis of PD models.

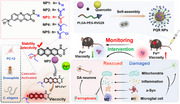

## 1 Introduction

Parkinson's disease (PD), the second most common neurodegenerative disease, is characterized by loss of doparminergic (DA) neurons and presence of Lewybody in substantia nigra (SN), accompanied by motor and non‐motor symptoms. Statistics showed that over 8.5 million individuals worldwide had PD in 2019, and this number was projected to reach 25 million by 2050 [1]. The incidence of PD has been annually increasing with the aging global population, making it become a major socioeconomic threat to the health and quality of life of middle‐aged and elderly individuals [2]. More notably, the pathological progression of PD is latent. By the time clinical symptoms appear, over 50% of DA neurons in the SN of brain of patients have been lost, making early detection and intervention crucial for better treatment of PD [3].

Currently, the diagnosis of PD mainly relies on clinical symptom assessment and bioimaging examinations, which have inevitable limitations. Clinical symptoms assessment is prone to being affected by subjective factors. Moreover, due to the existence of atypical symptoms, the misdiagnosis rate is as high as 20%–30% [4]. Bioimaging, such as magnetic resonance imaging (MRI) or positron emission tomography (PET), is able to detect pathological alteration in SN, but high cost and low equipment availability make it challenging as the routine detection option [5, 6]. Recently, biomarkers, including α‐Synuclein (α‐Syn) and Tau protein, were used in PD detection and exhibited some advantages for facile monitoring of pathological progress of PD, but the invasiveness, insufficient specificity and sensitivity restrict its application [7]. Fluorescent probes, with their advantages of high sensitivity, real‐time imaging capabilities, and non‐invasive detection strategies, providing a revolutionary strategy for the precise diagnosis of diseases. Ourself as well as other researchers have developed fluorescence‐based responsive probes to detect reactive species or enzymes and applied for PD investigation [8, 9, 10, 11, 12]. However, so far reported probes still have some drawbacks. For example, those probes target only one biomarker, the specificity of which is often compromised by complex biological environments [13, 14]. By constructing, cascade‐activated probes, by stepwise triggering fluorescent signal, improve the diagnostic specificity to over 90%, effectively eliminating the misdiagnosis risk caused by fluctuations of a single factor [15].

Ferroptosis is an iron‐dependent, non‐apoptotic type of programmed cell death pattern, which is implicated in various type of diseases, including PD [16, 17]. Ryan et al. demonstrated that ferroptosis related genes were substantially up‐regulated in the brain and blood samples of PD compared with that in control [18]. During the process of ferroptosis, overloaded iron ions, excessive reactive species, and elevated viscosity all get involved [19, 20]. Currently, in‐situ monitoring is a promising strategy for in‐depth understanding the role of ferroptosis in PD. In the occurrence of ferroptosis, aberrant accumulation of ferrous ion (Fe^2+^) is though to be the key initiator. In the SN of PD patients, excessive Fe^2+^ was observed, which catalytically generates a large amount of hydroxyl radicals through the Fenton reaction and leads to DA neurons loss [21, 22, 23, 24]. Viscosity is another mediator of ferroptosis [25]. We, in one recent study, found that the viscosity of the in vitro and in vivo PD models was increased [26]. However, to the best of our knowledge, there are no reported fluorescent probes that simultaneously monitoring Fe^2+^/Viscosity of ferroptosis in PD.

Considering the involvement of ferroptosis in PD pathogenesis, ferroptosis targeted intervention such as deferoxamine, resveratrol, quercetin (QC), has been proposed to treat PD [27, 28, 29]. Among them, QC is a natural active compound with anti‐inflammatory, antioxidant, and anti‐cell death properties, which has been utilized to treat various diseases including PD [30]. Notably, QC blocks ferroptosis‐related pathway and to chelates Fe^2^^+^ [31, 32]. However, QC has inherent defects, such as poor water solubility, low bioavailability, and insufficient blood‐brain barrier (BBB) penetration, which severely restrict its application [33, 34]. Nanoplatforms with good biocompatibility and targeted drug delivery capacity provide an effective strategy to solve these problems [35, 36]. In this context, a nanoplatform encapsulating the Fe^2+^/Viscosity fluorescent probe and QC may be an effective strategy for monitoring Fe^2+^/Viscosity in ferroptosis and verifying intervention effect in PD.

**SCHEME 1:**
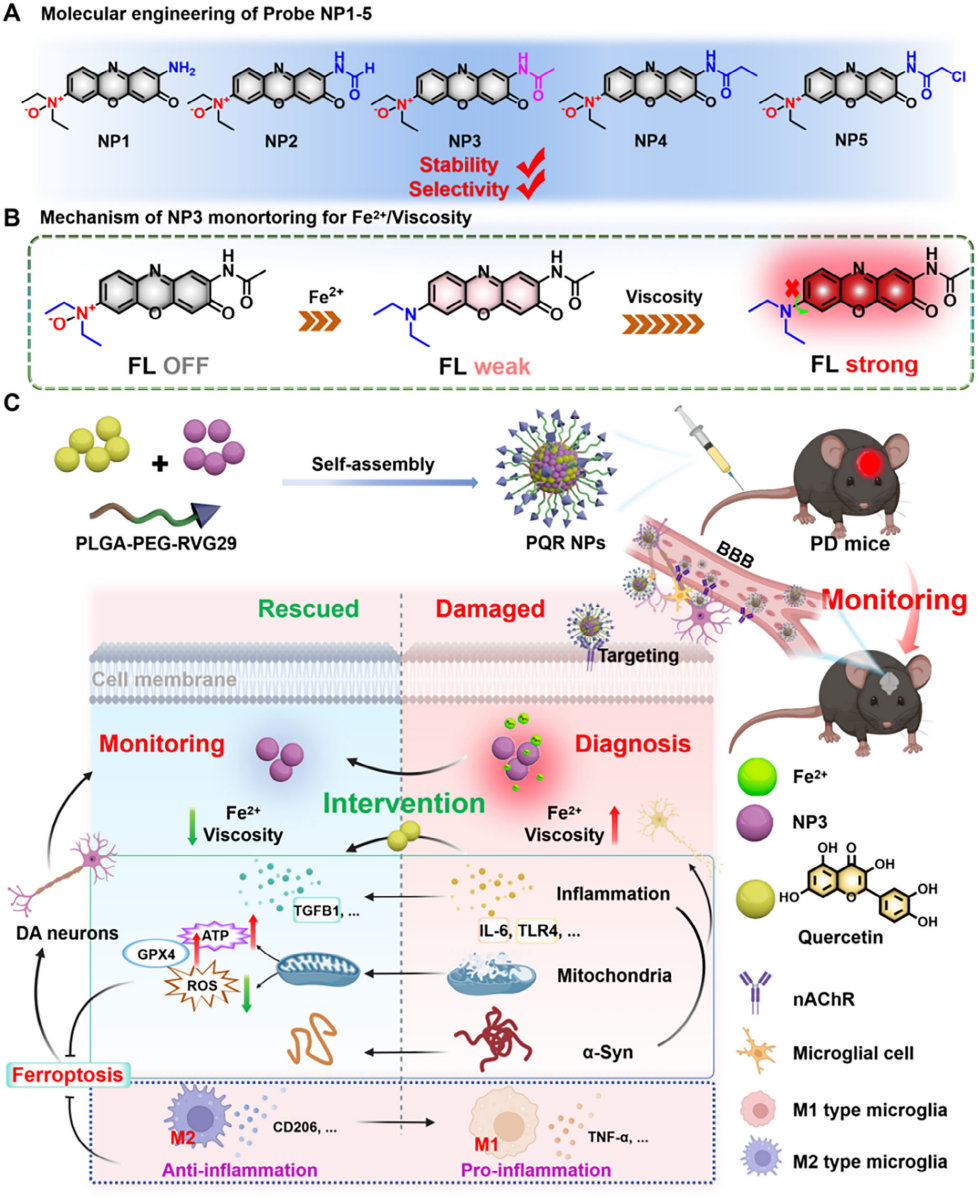
Design and bio‐application of Fe^2^^+^/Viscosity cascade‐activated NIR probe. (A) Structure of NP1–5. (B) Schematic diagram of the mechanism of NP3 response to Fe^2^^+^/Viscosity. (C) Schematic illustration of PQR NPs monitoring Fe^2^^+^/Viscosity in PD mice under intervention.

Herein, a Fe^2^^+^/Viscosity cascade‐activated near‐infrared (NIR) probe with high stability, sensitivity, and selectivity, is proposed for in‐situ diagnosis and monitoring of Fe^2+^/Viscosity in ferroptosis‐mediated PD (Scheme 1). Based on optical characterization and theoretical calculation toward Fe^2^^+^ and viscosity, NP3 was screened out among series probes NP1–5. To verify this concept for ferroptosis intervention in PD, an innovative nanoplatform, PQR NPs, is constructed by self‐assembly of NP3 and QC. As developed PQR NPs not only realized monitoring Fe^2+^/Viscosity for ferroptosis in PD but also provided an innovative concept “diagnosis‐monitoring” system for neurodegenerative diseases.

## 2 Results and Discussion

### 2.1 Synthesis and Characteristic of Fe^2^^+^/Viscosity Responsive Probes NP1–5

**FIGURE 1:**
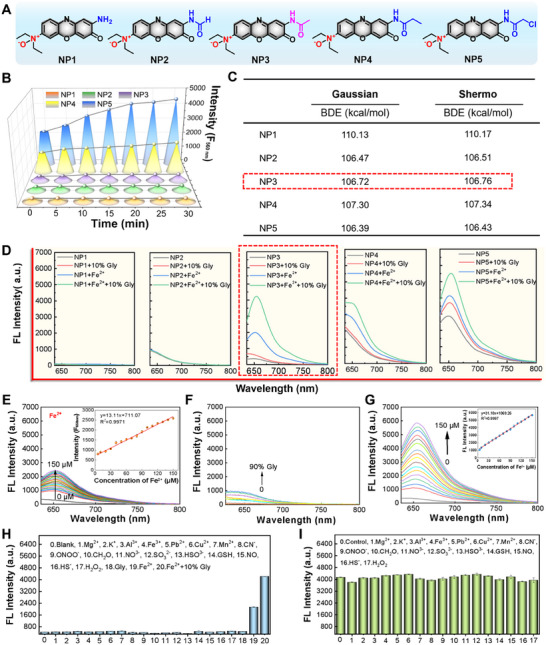
Synthesis and characterization of NP1–5. (A) Structures of NP1–5. (B) FI of NP1–5 over different time. (C) Theoretical calculation of BDE of N─O bond for NP1–5. (D) Fluorescence spectra of NP1–5 in the presence or absence of Fe^2^^+^, Gly, and Fe^2^^+^/Gly. (E) Fluorescence spectra of NP3 in response to different concentrations of Fe^2^^+^, with the inset showing the linear relationship between FI and Fe^2^^+^ concentration. (F) Fluorescence spectra of NP3 in response to Gly of different volume percentages. (G) Fluorescence spectra of NP3 in response to different concentrations of Fe^2^^+^ with 10% Gly, with the inset showing the linear relationship between FI and Fe^2^^+^ concentration. Data presented as mean ± SD; *n* = 3 for each. (H) Selectivity of NP3 towards relevant analytes. (I) Anti‐interference of NP3 against relevant analytes.

Accumulated Fe^2^^+^ and increased intracellular viscosity were featured alteration of ferroptosis. Based on the structural properties of N‐oxide and N,N‐dimethyl groups [37, 38], we designed and synthesized a series of Fe^2^^+^/Viscosity cascade‐activated NIR fluorescent probes (NP1–5) (Scheme S1). Phenoxazinone was used as the fluorescent scaffold, and their radiative transition was regulated by acylation of the ─NH_2_ group with different substituents. Meanwhile, the N‐oxide group in the probes could specifically respond to Fe^2^^+^, and generated N,N‐diethyl groups which sensitively recognize viscosity, ultimately successfully constructing Fe^2^^+^/Viscosity cascade‐activated probes. The target probes were prepared by oxidizing the precursors Pre‐NP1–5 with m‐chloroperoxybenzoic acid, and their structures were characterized by nuclear nagnetic resonance spectroscopy (NMR) and high resolution mass spectrometry (HR‐MS) (Figures S1–S15). The structures of N‐oxidized phenoxazinone derivatives, NP1–5, were phenoxazinone‐amino, phenoxazinone‐formamido, phenoxazinone‐acetamido, phenoxazinone‐acrylamido, and phenoxazinone‐chloroacetamido, respectively (Figure 1A). First, we investigated the stability of probes NP1–5. It showed that probes NP1‐4 exhibited good stability, while NP5 had relatively poor stability possibly due to the electron‐withdrawing effect of the chlorine atom (Figure 1B). N‐oxide probes can be converted into their corresponding amine derivatives by breaking the N─O bond, thereby altering the intramolecular charge transfer (ICT) properties [39, 40]. Herin, we investigated the bond dissociation energy (BDE) of the N─O bond in NP1–5 via theoretical calculations. As shown in Figure 1C, NP3 showed moderate BDE of the N─O bond, which might account for its high stability and also suggested the optimal responsive capability toward Fe^2^^+^. Further, we used a fluorescence spectrometer to investigate the optical properties of NP1–5 toward Fe^2^^+^, glycerol (Gly), and Fe^2^^+^/Gly. NP1 and NP2 showed no responsiveness; NP4 and NP5 had responsiveness accompanied by excessively high background; and NP3 displayed good responsiveness with neglectable background, establishing it as the optimal candidate (Figure 1D). It was consistent with the above N─O bond BDE theoretical calculation results. Subsequently, we tested the response time of NP3 to Fe^2^^+^, Gly, and Fe^2^^+^/Gly, and its stability with different temperatures and pH. The results showed that the fluorescence signal of NP3 in response to the Fe^2^^+^/Gly mixed system reached a plateau at 2.5 min (Figure S16), which showed almost no obvious change under different pH (Figure S17) and temperatures (Figure S18). It indicated that NP3 had a fast response speed to Fe^2^^+^, Gly, and the Fe^2^^+^/Gly as well as high stability. Furthermore, fluorescence spectra of probe NP3 showed that the fluorescence intensity (FI) moderately increased with Fe^2^^+^ concentration, with a detection limit of 1.79 µM (Figure 1E), and only slightly increased with the volume percentage of Gly (Figure 1F). Notably, within 10% Gly, NP3 fluorescence intensity significantly increased with Fe^2^^+^ concentration, with a detection limit as low as 0.75 µM (Figure 1G). It indicated that fluorescence signal of NP3 was cascade‐activated by simultaneous presence of Fe^2^^+^/Viscosity. Given the complexity of biological environments, we validated the selectivity and anti‐interference of NP3 in response to Fe^2^^+^/Viscosity in the presence of relevant bioanalytes. The results showed that the probe exhibited good selectivity (Figure 1H) and anti‐interference ability (Figure 1I). Collectively, above results demonstrated that NP3 had high sensitivity, excellent stability, and good selectivity, making it a high‐performance Fe^2^^+^/Viscosity cascade‐activated probe.

### 2.2 Response Mechanism of NP3 Toward Fe^2^^+^/Viscosity

**FIGURE 2:**
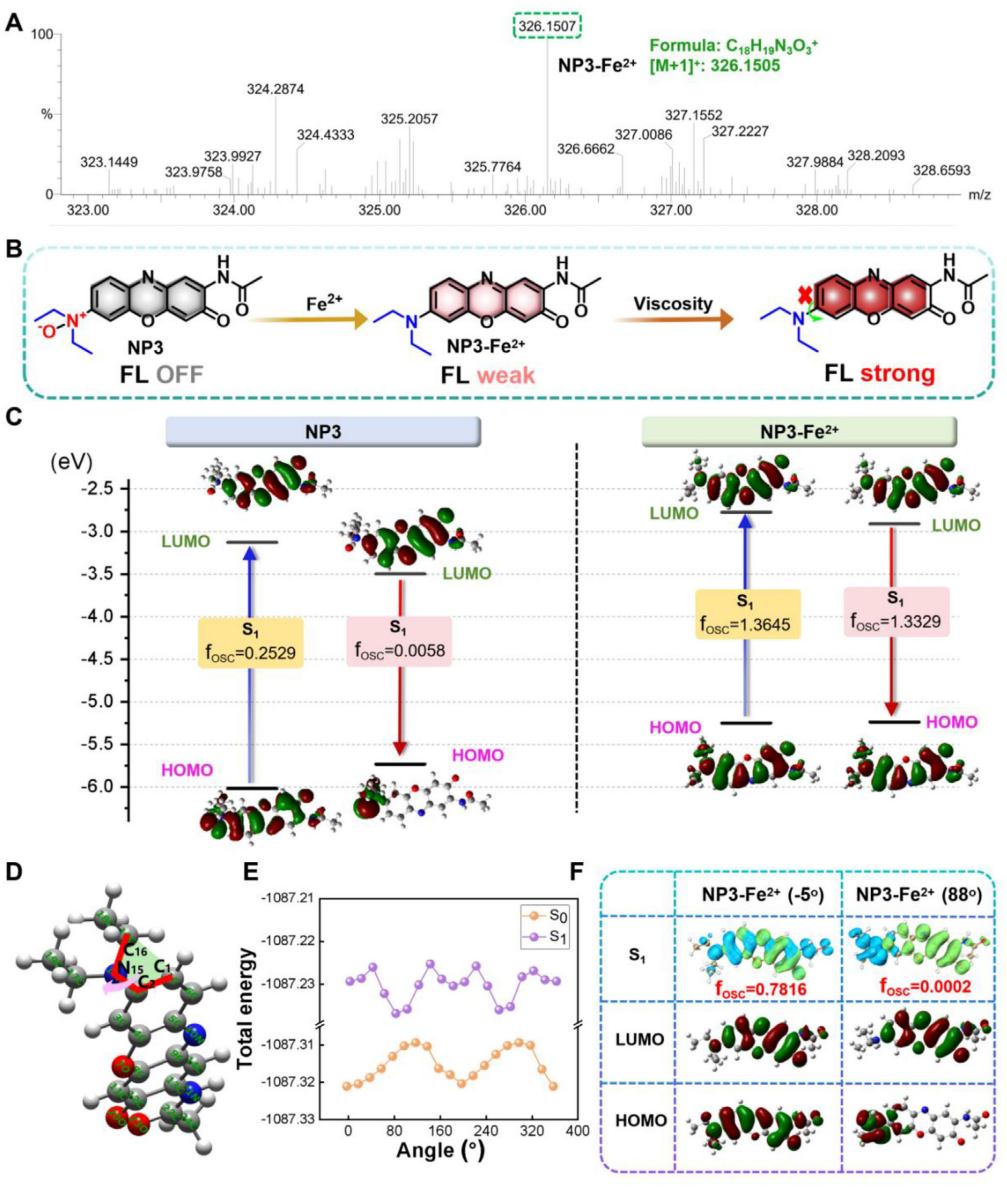
Response mechanism of NP3 toward Fe^2^^+^/Viscosity. (A) Mass spectrum of NP3 and Fe^2^^+^ reacting solution. (B) Schematic diagram of the response mechanism of NP3 to Fe^2^^+^/Viscosity. (C) Theoretical modeling of HOMO/LUMO orbital energies, calculated electronic excitation energies (eV) of major transitions, and the corresponding f_osc_. of NP3 before and after response to Fe^2^^+^. (D) Schematic diagram of dihedral angle rotation in the NP3‐Fe^2^^+^ structure. (E) Energy curve showing the variation of energy of NP3‐Fe^2^^+^ with dihedral angle in the range of 0°–360°. (F) Hole‐electron distribution and corresponding f_osc_, theoretical modeling of HOMO/LUMO energies of the S_1_ state of the NP3‐Fe^2^^+^ at different dihedral angles, of which blue represents electrons and green represents holes.

After clarifying the optical properties of NP3 in response to the Fe^2^^+^/Viscosity, we further explored its response mechanism using mass spectrometry (MS) and theoretical calculations. MS results showed that the N‐oxide moiety in NP3 was reduced to a tertiary amine after the addition of Fe^2^^+^ (Figure 2A). This finding demonstrated that the lone pair electrons localized on the nitrogen atom of the N‐oxide moiety of NP3 were unable to form an effective conjugated system with the fluorescent chromophore, resulting in the fluorescence quenching of the probe. In contrast, in the presence of Fe^2^^+^, the in‐situ generated N,N‐diethylamino group of NP3 re‐established a conjugated framework with the aromatic ring of the fluorescent chromophore, leading to enhanced fluorescence. Importantly, this observation was in consistent with the aforementioned fluorescence spectroscopy results. Therefore, we proposed a mechanism for the response of NP3 to the Fe^2^^+^/Viscosity (Figure 2B), where in the N,N‐diethyl group generated from the reaction between NP3 and Fe^2^^+^ presumably dominated the optical property changes. To further reveal the photophysical mechanism of NP3 in response to Fe^2^^+^/Viscosity, theoretical calculations on its excitation and emission processes were performed. The generation of fluorescence depended on the radiative transition of molecules from the lowest singlet excited state (S_1_) to the ground state (S_0_) [41]. The oscillator strength (f_o__s__c_) was a physical quantity that described the probability of absorbing electromagnetic radiation during the transition between atomic or molecular energy levels, and it could be used to determine whether a radiative transition or non‐radiative transition occurred in the emissive state [42, 43]. The f_osc_ for the S_0_→S_1_ and S_1_→S_0_ transitions of NP3 were 0.2529 and 0.0058, respectively. After the reaction of NP3 with Fe^2^^+^, the f_osc_ for the S_0_→S_1_ and S_1_→S_0_ transitions of the resulting system significantly increased to 1.3645 and 1.3329, respectively (Figure 2C). This finding demonstrates that the transition behavior of NP3 is primarily governed by non‐radiative transitions, attributed to its relatively low f_osc_. However, after NP3 interacted with Fe^2^^+^, the luminescence mechanism of the system was dominated by radiative transitions, which ultimately manifested as enhancement of the fluorescence signal at the macroscopic level. Although the FI of NP3‐Fe^2^^+^ was enhanced compared with that of NP3 only, the overall FI remained relatively weak. We hypothesized that this observation could be attributed to the N,N‐diethylamino group in the NP3‐Fe^2^^+^, which had been proved possessing rotatable properties and participating in the non‐fluorescent twisted intramolecular charge transfer (TICT) process [44]. Therefore, the C1‐C2‐N15‐C16 bond at the linkage between the N,N‐diethylamino group and the benzene ring was selected as the dihedral angle rotation axis (Figure 2D), and density functional theory (DFT) and time‐dependent DFT (TD‐DFT) methods were used to calculate the energy variation of the NP3‐Fe^2^^+^ with the dihedral angle [45]. As shown in Figure 2E, the S_0_ state energy curve of NP3‐Fe^2^^+^ remained smooth, while the S_1_ state exhibited multiple energy peaks. This result indicated that the energy of the excited state increased with the torsion of the dihedral angle, further confirming the existence of the TICT process. Furthermore, an analysis of the energy levels of the NP3‐Fe^2^^+^ at different dihedral angles revealed that, when the dihedral angle between the N,N‐diethylamino group and the benzene ring rotated to a perpendicular state, the f_osc_ significantly decreased from 0.7816 to 0.0002. Concurrently, a distinct separation of electrons and holes occurred, leading to the formation of a TICT state (Figure 2F). Above results confirmed that the response mechanism of NP3 toward Fe^2^^+^/Viscosity was based on the redox reaction and TICT, which ultimately achieved the cascade activation of fluorescence signal.

### 2.3 NP3 Monitoring Fe^2^^+^/Viscosity In Vitro and in vivo PD Models

**FIGURE 3:**
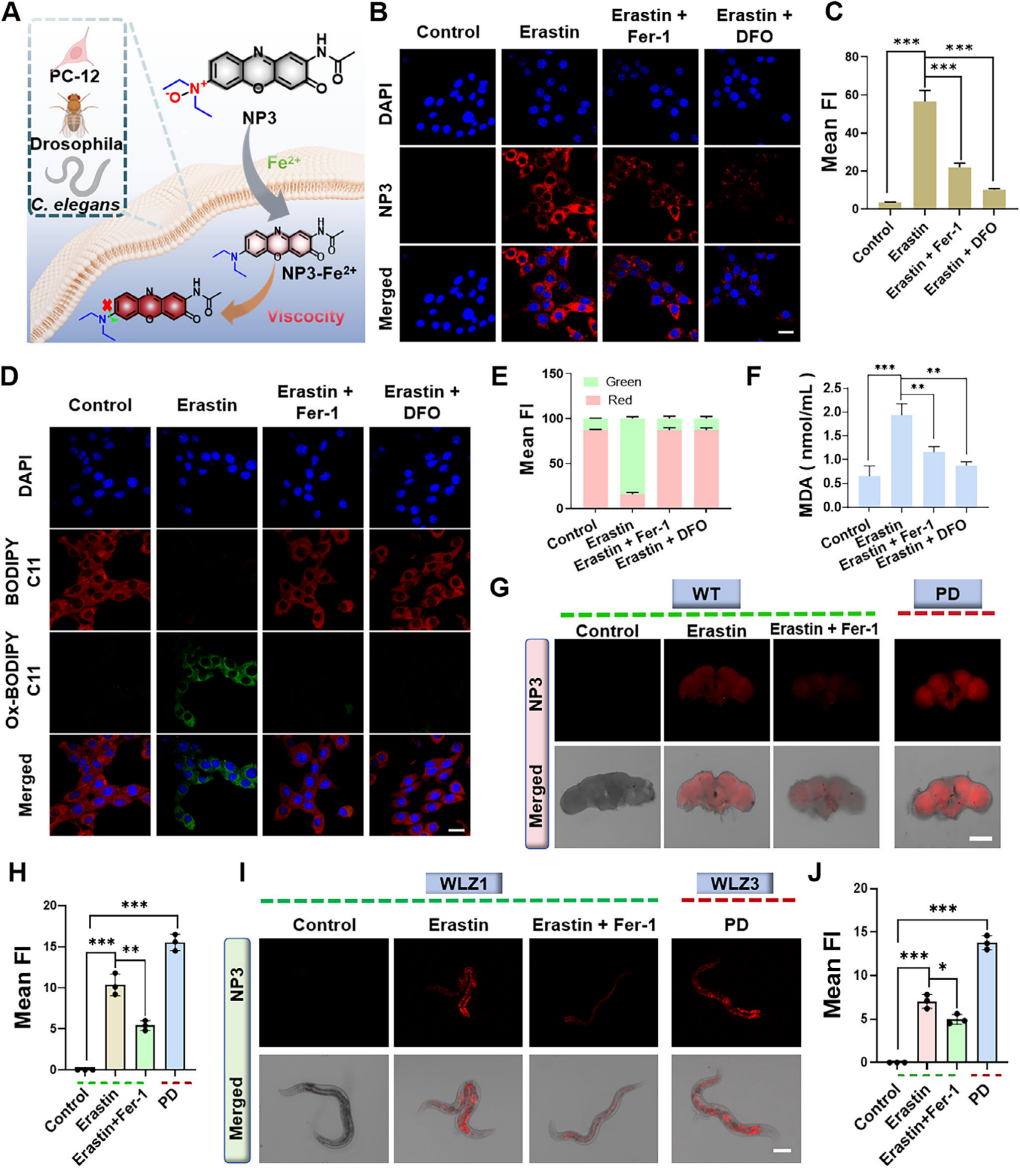
NP3 imaging Fe^2+^/Viscosity in vitro and in vivo PD models. (A) Schematic diagram of the probe for monitoring Fe^2+^/Viscosity in cells, Drosophila, and *C. elegans*. (B) Confocal imaging and (C) Quantitative statistical graph of PC‐12 cells after different treatment incubated with NP3 (10 µM) at 37°C for 30 min (Scale bar: 20 µm). (D) Confocal imaging and (C) Quantitative statistical graph of PC‐12 cells after different treatment incubated with BODIPY 581/591 C11 (10 µM) at 37°C for 30 min (Scale bar: 20 µm). (F) MDA levels of PC‐12 cells with different treatments. (G) Confocal imaging and (H) Quantitative statistical graph of WT Drosophila and PD Drosophila with different treatment (Scale bar: 200 µm). (I) Confocal imaging and (J) quantitative statistical graph of *C. elegans* with different treatment (Scale bar: 200 µm). Data presented as mean ± SD, *n* = 3 for each, *ANOVA test*, *p < 0.05, **p < 0.01, ***p < 0.001.

After clarifying the optical response performance and mechanism of NP3 toward Fe^2^^+^/Viscosity, we further investigated its imaging capability in cells, Drosophila, and *Caenorhabditis elegans* (*C. elegans*) PD models (Figure 3A). First, we assessed the biosafety of NP3 by MTT assays. As shown in Figure S19, in the presence of NP3 up to 20 µM, the cell viability of PC‐12 cells was above 80%, indicating its good biocompatibility. Subsequently, we investigated the response of NP3 to exogenous and endogenous Fe^2^^+^ and viscosity in PC‐12 cells by confocal imaging. As shown in Figure S20, NP3 could specifically respond to Fe^2^^+^ in PC‐12 cells, exhibiting a gradient increase in red fluorescence signal with the increase of Fe^2^^+^ concentrations; no discernible fluorescence signal was observed in the starvation‐induced high‐viscosity PC‐12 cell model. However, under the synergistic condition of high Fe^2^^+^/viscosity, fluorescence signal markedly intensified. Given the involvement of ferroptosis in PD pathogenesis, we induced a cellular PD model by administration of the ferroptosis activator, Erastin, which had been proved to induce elevated Fe^2^^+^/Viscosity [46, 47]. First, Erastin induced cellular PD model was verified by assessing the expression of tyrosine hydroxylase (TH), the rate limiting enzyme for dopamine synthesis. As shown in Figure S21, the Erastin treated PC‐12 cells showed a significant decrease of TH, whereas the ferroptosis inhibitor (Ferrostatin‐1, Fer‐1) treatment substantially reversed the reduction of TH. To investigate whether NP3 could monitor the alteration of endogenous Fe^2^^+^/viscosity in cellular PD model, we administrated Fer‐1 or deferoxamine (DFO, an iron chelator) into Erastin treated PC‐12 cells. As shown in Figure 3B,C, the PC‐12 cells treated with Erastin exhibited the strongest fluorescence signal derived from NP3, while it was significantly weakened upon the addition of the Fer‐1 or DFO. These results confirmed that NP3 could monitor alteration of endogenous Fe^2^^+^/Viscosity. To clarify Erastin induced ferroptosis in PC‐12 cells, the intracellular lipid peroxides (LPO) and malondialdehyde (MDA) levels, which constituted core endpoints for ferroptosis, were determined by commercial probe. Cellular imaging revealed that, Erastin treatment significantly promoted LPO accumulation within PC‐12 cells, as manifested by marked increased greenfluorescence signal; which was substantially reduced by Fer‐1 or DFO administration (Figure 3D,E). MDA content assays revealed that it was significantly elevated with treatment of Erastin, which was reduced by Fer‐1 or DFO (Figure 3F). The above results suggested that Erastin could induce ferroptosis in PC‐12 cells, and NP3 enabled to monitor altered Fe^2^^+^/viscosity in ferroptosis. Moreover, we evaluated the ability of NP3 to monitor Fe^2^^+^/Viscosity in Drosophila or *C. elegans* PD model. We first established Erastin induced Drosophila PD model and verified through behavioral analysis and DA neuron imaging (Figure S22). Thereafter, wild‐type (WT) Drosophila, Erastin induced WT Drosophila, Fer‐1‐treated Erastin induced WT Drosophila, and Parkin‐null PD Drosophila were selected to assess the imaging capacity of NP3. As shown in Figure 3G,H, the brain of Erastin induced or Parkin null PD Drosophila exhibited the strongest fluorescence signals, while the Fer‐1 treatment significantly reduced the fluorescence signals in Erastin induced PD Drosophila. These results demonstrated that NP3 could distinguish PD Drosophila from the control by detecting elevated Fe^2^^+^/Viscosity. After that, we investigated the ability of NP3 to monitor Fe^2^^+^/Viscosity in *C. elegans* PD model. Wild‐type *C. elegans* (WLZ1) were treated with Erastin, and the successful establishment of the *C. elegans* PD model was confirmed via behavioral analysis and DA neuron imaging (Figure S23). After incubation with the probe NP3, the Erastin‐treated WLZ1 *C. elegans* as well as LRRK2 overexpressed transgenic PD *C. elegans* (WLZ3) exhibited stronger fluorescence signal compared with control, while the Fer‐1‐treated *C. elegans* showed reduced fluorescence signal (Figure 3I,J), indicating that the probe could distinguish between control and PD *C. elegans*. Summarily, the above results demonstrated that NP3 could visually monitor elevated Fe^2^^+^/Viscosity, thereby distinguishing PD in vitro and in vivo.

### 2.4 Preparation and Characterization of PQR NPs

**FIGURE 4:**
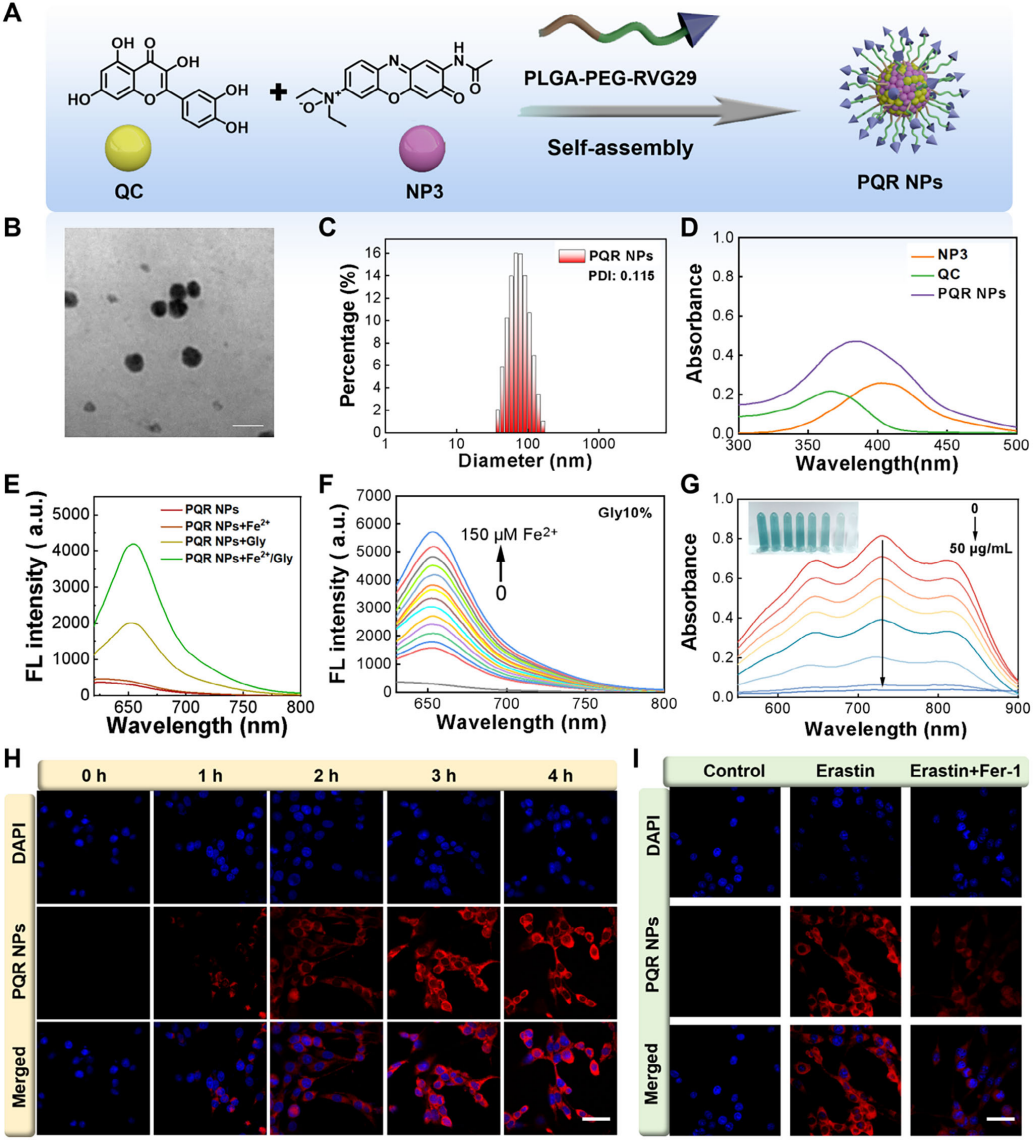
Preparation and characterization of PQR NPs. (A) Schematic diagram of PQR NPs construction. (B) TEM image of PQR NPs (Scale bar: 100 nm). (C) DLS histogram of PQR NPs size distribution. (D) Absorption spectra of NP3, QC, and PQR NPs. (E) Fluorescence spectra of PQR NPs (50 µg/mL) with Fe^2^^+^, Gly, Fe^2+^/Gly. (F) Fluorescence spectra of PQR NPs (50 µg/mL) with concentrations ranging from 0 to 150 µM at a fixed Gly volume fraction (10%). (G) Absorption spectra and corresponding color changes of ABTS after reacting with different concentration PQR NPs. (H) Fluorescence imaging PC‐12 with PQR NPs (50 µg/mL) at different time points (Scale bar: 20 µm). (I) Fluorescence imaging of PC‐12 cells with PQR NPs (50 µg/mL) after treated by Erastin, Erastin+Fer1 (Scale bar: 20 µm).

Following the performance validation of NP3, to achieve in vivo monitoring Fe^2+^/Vicosity ferroptosis based PD therapeutic efficacy, we constructed the nanoplatform PQR NPs by self‐assembly of NP3, ferroptosis inhibitor QC, and modified with penetrating peptide rabies virus glycoprotein‐29 (RVG‐29) (Figure 4A). Transmission electron microscopy (TEM) and dynamic light scattering (DLS) results revealed that PQR NPs were spherical nanoparticles with an average diameter of approximately 100 nm (Figure 4B,C). Absorption spectra showed that PQR NPs exhibited a characteristic absorption peak at 380 nm (Figure 4D), which further confirmed the successful construction of PQR NPs. The drug loading efficiency of QC and probe NP3 was determined via high‐performance liquid chromatography (HPLC), and it got to be 10.41% and 7.58%, respectively. Optical tests revealed that PQR NPs showed weak responses to Fe^2^^+^ or viscosity alone (Figures S24 and S25), and fluorescence signals were only substantially triggered in the simultaneous presence of Fe^2^^+^ and Viscosity (Figure 4E), which exhibited concentration‐dependent manner (Figure 4F). QC was a bioactive compound with antioxidant activity [48]. Therefore, we evaluated the free radical scavenging capacity of PQR NPs using a commercial probe, 2,2'‐azino‐bis(3‐ethylbenzthiazoline‐6‐sulfonic acid (ABTS). As the PQR NPs concentration increased, the absorbance of ABTS at 734 nm gradually decreased, accompanied by a distinct color change, confirming that PQR NPs possessed excellent free radical scavenging activity (Figure 4G). After that, we further investigated the cellular uptake efficiency and imaging capability of PQR NPs. It showed that PQR NPs were liable to be uptook by cells and it reached a plateau after 3 h (Figure 4H; Figure S26). Importantly, PQR NPs monitored Fe^2^^+^/Viscosity in ferroptosis activated or inhibited PC‐12 cells (Figure 4I; Figure S27). Above results proved that a self‐assembly nanoplatform, PQR NPs, with Fe^2^^+^/Viscosity monitoring and reactive oxygen species (ROS) scavenging capacity was successfully prepared.

### 2.5 Neuroprotective Effect of PQR NPs on Cellular PD Model

**FIGURE 5:**
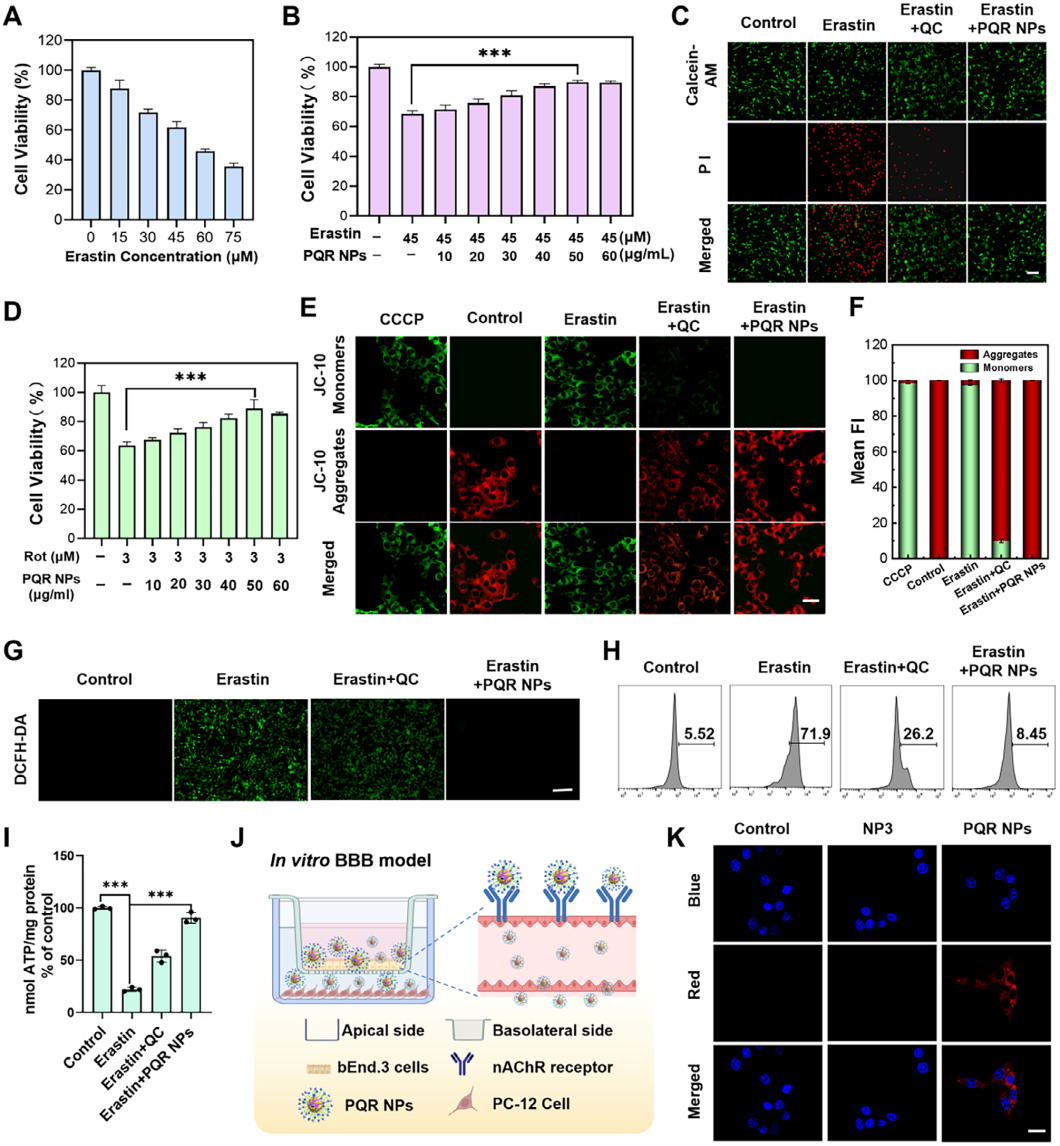
Neuroprotective effect of PQR NPs on cellular PD models. (A) Viability of PC‐12 cells with different concentration Erastin. (B) Concentration‐dependent protective effects of PQR NPs on Erastin induced PC‐12 cells. (C) Calcein‐AM/PI double‐staining PC‐12 cells in different treatment groups (Scale bar: 100 µm). (D) Concentration‐dependent protective effects of PQR NPs on Rot‐induced PC‐12 cells. (E) Fluorescence imaging and (F) Quantitative statistical graph of MMP in PC‐12 cells of different treatments labeled with JC‐10 probe (Scale bar: 20 µm). (G) Fluorescence imaging and (H) Flow cytometry results of ROS in PC‐12 cells of different treatments labeled with DCFH‐DA probe (Scale bar: 100 µm). (I) ATP levels in PC‐12 cells with different treatments. (J) Schematic diagram of the Transwell double‐chamber co‐culture system. (K) Fluorescence imaging of PC‐12 cells in the lower chamber of Transwell (Scale bar: 15 µm). Data presented as mean ± SD, *n* = 3 for each, *Student's t‐test*, ***p < 0.001.

After confirming that PQR NPs could visually monitor Fe^2+^/Viscosity in the cellular PD model, we further conducted systematic investigations to evaluate its neuroprotective performance. First, we created one cellular PD model by administration of Erastin. As shown in Figure 5A, Erastin treatments significantly induced PC‐12 cell death as assessed by MTT. 45 µM Erastin rendered 60% cell death, which was adopted for the following experiments. Then, we assessed the neuroprotective effect of PQR NPs. PQR NPs effectively rescued Erastin induced PC‐12 cell death as confirmed using the MTT assay and AM/PI imaging. Notably, the intervention effect of PQR NPs was superior to that of the QC treatment (Figure 5B,C). In addition, the neuroprotective effect of PQR NPs was further verified with the classic PD inducing neurotoxin rotenone (Rot). It showed that PQR NPs also significantly increased the viability of Rot treated PC‐12 cells (Figure 5D; Figures S28 and S29). Mitochondria, as the central hub of cellular energy metabolism, played a key role in maintaining cell survival [49]. Herein, we investigated the effects of PQR NPs on mitochondrial function (membrane potential, ROS content, and ATP level) in Rot and Erastin induced PD cell models. The JC‐10 probe was used to detect changes in mitochondrial membrane potential (MMP). As shown in Figure 5E,F, the Erastin‐treated PC‐12 cells exhibited enhanced green fluorescence signal, indicating severe depolarization of MMP. In contrast, the PQR NPs treatment reversed Erastin induced MMP depolarization as indicated by enhanced red fluorescence and reduced green fluorescence. Subsequently, the DCFH‐DA probe was used to evaluate intracellular ROS levels. Cell imaging (Figure 5G) and flow cytometry (Figure 5H) results consistently showed PQR NPs treatment significantly alleviated ROS accumulated in Erastin induced PC‐12 cell. Moreover, ATP detection kit was used for quantitative analysis of ATP content in different treated PC‐12 cells. As shown in Figure 5I, PQR NPs intervention significantly increased cellular ATP content which was reduced by Erastin treatment. Consistently, PQR NPs administration restored mitochondria function disrupted by Rot (Figures S30–S32). The BBB served as the barrier to protect brain from exogenous invasive injury, but it also restricted the drugs form reaching into brain [50]. To verify whether PQR NPs could cross the BBB, we established an in vitro BBB model using a Transwell double‐chamber co‐culture system. The working principle of system was clearly illustrated via a schematic diagram (Figure 5J). PQR NPs were added into the upper chamber, and red fluorescent signals of PQR‐NPs were observed for 12 h in Erastin treated PC‐12 cells in the lower chamber, whereas there was no visible fluorescent signals from cells subjected to NP3 (Figure 5K; Figure S33). Collectively, it demonstrated that PQR NPs could not only exerted neuroprotective effect on Erastin and Rot induced cellular PD models by restoring mitochondria function, but also penetrated in vitro BBB model, indicating the in vivo application potential.

### 2.6 Neuroprotective Mechanism of PQR NPs on Cellular PD Model

**FIGURE 6:**
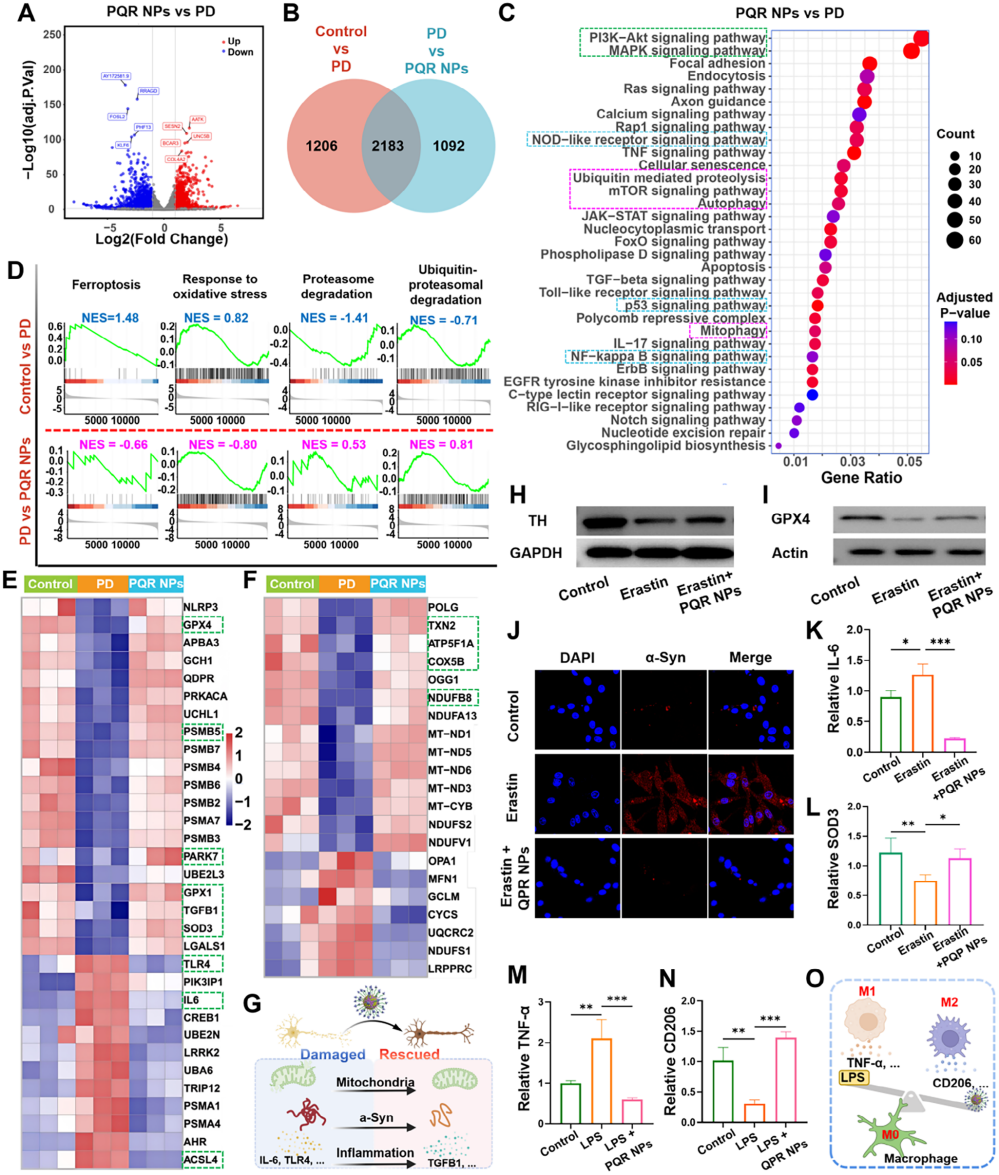
Neuroprotective mechanism of PQR NPs. (A) Volcano plot showing DEGs in PD and PQR NPs‐treated group. (B) Venn diagram showing DEGs in the different groups. (C) KEGG enrichment analysis of DEGs between PD and PQR NPs‐treated group. (D) GSEA analysis presenting the signaling pathways in the different groups. Heatmap showing DEGs related to (E) ferroptosis, oxidative stress, protein degradation, and (F) mitochondria among different groups. (G) Schematic diagram of the mechanism by which PQR NPs alleviate cellular ferroptosis. WB analysis of (H) TH and (I) GPX4 expression. (J) α‐Syn IFL staining in different groups. qPCR validation of (K) IL‐6 and (L) SOD3 mRNA levels in PC‐12 cells, and (M) TNF‐α and (N) CD206 mRNA levels in HMC3 cells treated with different groups. (O) Schematic diagram of the mechanism by which PQR NPs alleviate macrophage polarization. Data presented as mean ± SD, *n* = 3 for each, *ANOVA test*, *p < 0.05, **p < 0.01, and ***p < 0.001.

Given the aforementioned neuroprotective performance of PQR NPs, we performed sequencing analysis to further clarify the underlying mechanism. The PC‐12 cells were classified into three groups, untreated control, Erastin induced PD, and Erastin+PQR NPs combined intervention. Volcano plots were used to visualize the differential expression genes (DEGs) among groups. DEGs between the control and the Erastin induced PD group were 3389, with 2035 upregulated and 1354 downregulated (Figure S34). DEGs between PD model and PQR NPs treated group were 3275, including 1143 upregulated and 2132 downregulated (Figure 6A). Venn diagram analysis revealed there were 2183 overlapped DEGs among control, PD, and PQR NPs treated groups (Figure 6B). Kyoto Encyclopedia of Genes and Genomes (KEGG) enrichment analysis showed that DEGs between the control and Erastin induced PD group were significantly enriched in pathways related to PD pathology including neurodegeneration pathways, MAPK and PI3K‐Akt signal pathways, et al. (Figure S35). DEGs between the PQR NPs and PD groups were enriched in pathways related to cell survival regulation, neural function repair, and stress response. Among them, TNF, NF‐κB, and Toll‐like receptor pathways got involved in inflammation; PI3K‐Akt and MAPK signaling pathways were related to DA neuron damage and oxidative stress; autophagy, mitophagy, and ubiquitin‐mediated proteolysis pathways were related to protein and mitochondria quality control (Figure 6C). Gene Set Enrichment Analysis (GSEA) revealed that, compared with the control, in PD group the genes involved in ferroptosis and oxidative stress were up‐regulated, while those related to proteasomal degradation and ubiquitin‐proteasomal degradation were down‐regulated. Whereas, PQR NPs treatment reversed the alteration of genes in PD (Figure 6D; Figure S36). To define the crucial genes involved in aforementioned pathways, Heatmaps analysis was conducted. It showed that in the PD group, pro‐ferroptosis gene such as ACSL4 was up‐regulated, while anti‐ferroptosis gene such as GPX4 was down‐regulated; pro‐inflammation genes such as IL6, TLR4 were up‐regulated; anti‐inflammation genes such as TGFB1 was down‐regulated; protein degradation related genes such as PSMB5, PAPK7 were down‐regulated; anti‐oxidation gene such as SOD3, GPX1 were down‐regulated. As expected, the regulation of above‐mentioned genes in PD was rigorously reversed by PQR NPs treatment (Figure 6E). Additionally, mitochondria function related genes such as ATP5F1A, COX5B, NDUFB8, and TNX2 were down‐regulated, which were all reversed by POR NP treatment (Figure 6F). Collectively, these results demonstrated that PQR NPs exerted neuroprotective effects by restoring mitochondrial function, enhancing antioxidant capacity, while inhibiting ferroptosis, alleviating inflammation, and promoting protein degradation (Figure 6G). To validate the transcriptome results, we performed WB, immunofluorescence (IFL) staining, and Quantitative polymerase chain reaction (qPCR) analyses. It showed that PQR NPs restored the expression of TH and GPX4 in the PD cells (Figure 6H,I; Figures S37 and S38). IFL results indicated that PQR NPs reduced α‐Syn aggregation in PC‐12 induced by Erastin (Figure 6J; Figure S39). The qPCR assay revealed that PQR NPs significantly inhibited expression of proinflammatory factors, IL‐6, TLR4, and restored anti‐oxidation SOD3 expression (Figure 6K–L; Figure S40). As well known, macrophage polarization (M1∼M2) was involved in ferroptosis. Therefore, we validated the impact of PQR NPs on polarization in human microphage (HMC3). As shown in Figure 6M,N, LPS treatment induced up‐regulation of M1 biomarker (TNF‐α), and reduced M2 biomarker (CD206, mannose receptor C‐type 1) expression. This observation was robustly reversed by PQR NPs treatment, indicating that PQR NPs could regulate macrophage polarization (Figure 6O). In summary, above systematic evidences from transcriptomic, proteomic, and cellular functional levels comprehensively clarify the neuroprotective mechanism of PQR NPs.

### 2.7 PQR NPs Monitoring Fe^2+^/Viscosity in PD Mice

**FIGURE 7:**
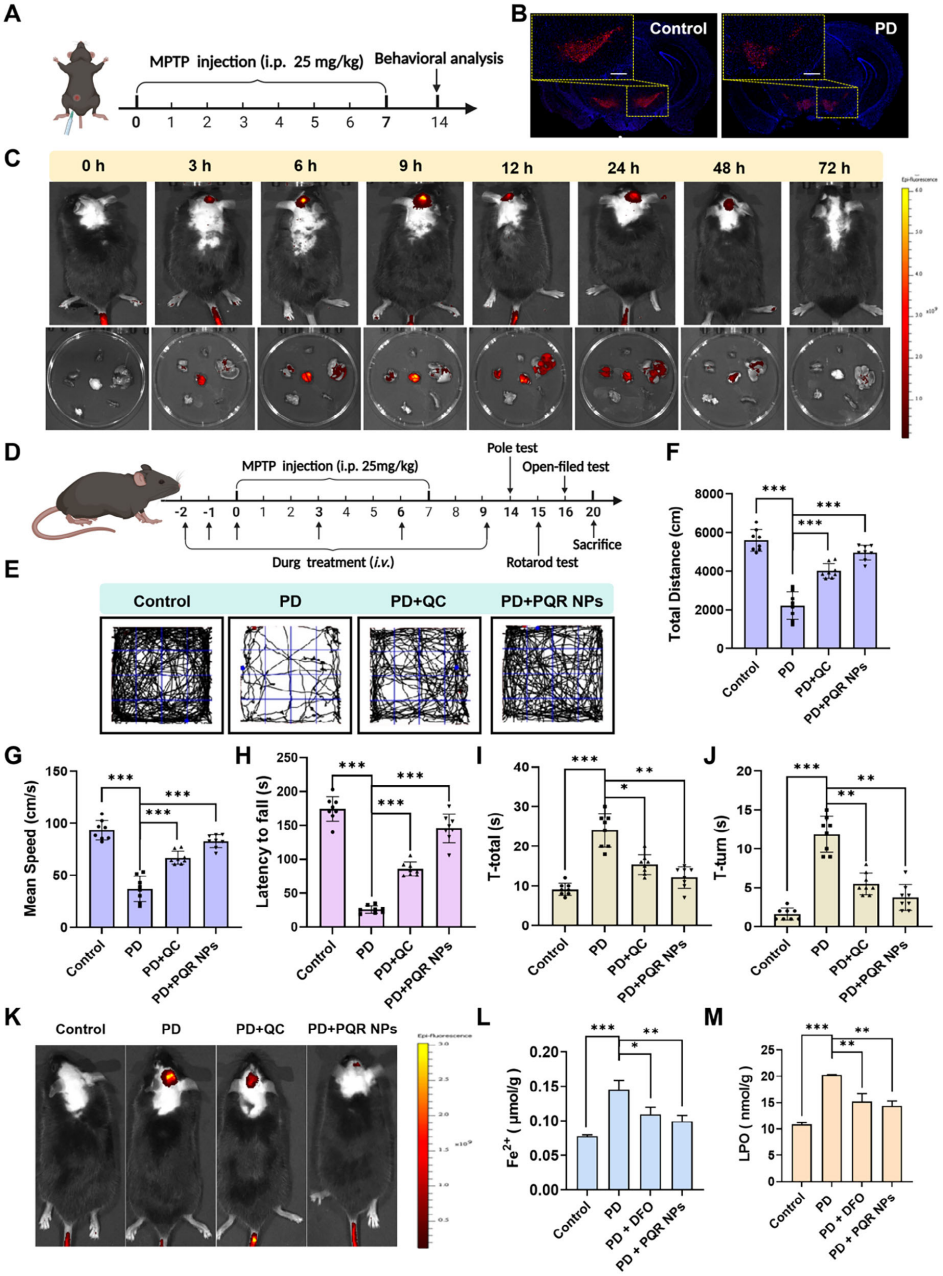
PQR NPs monitoring Fe^2+^/Viscosity in MPTP induced PD mice. (A) Schematic diagram of the construction process for MPTP induced mice PD model. (B) IFL staining images of DA neurons of PD and control mice (scale bar: 200 µm). (C) in vivo imaging of PD mice at different time points after PQR NPs administration (up), and corresponding fluorescence imaging of various organs (down). (D) Schematic diagram of PQR NPs treatment process on PD mice. (E) Movement trajectory diagrams, (F) Total distance, (G) Mean speed, (H) Number of entries into the central zone, (I) Distance traveled in the central zone, (J) Latency of drop off in the rotarod test (*n* = 8). (K) PQR NPs monitoring the intervention effect on PD mice. (L) Fe^2^^+^ and (M) LPO content in the substantia nigra of mice brain tissues among different treatment groups (*n* = 3). Data presented as mean ± SD, *ANOVA test*, *p < 0.05, **p < 0.01, and ***p < 0.001.

Based on the afore promising performance of PQR NPs, we wish to verify its application in mammalian PD model. First, we established the MPTP induced mice PD model, within which we validated the performance of the PQR NPs (Figure 7A) [51]. TH IFL staining showed a significant reduction of DA neurons in the SN of PD mice (Figure 7B). Behavioral assays, including open‐field test (Figure S41), pole test (Figure S42), and rotarod test (Figure S43), revealed obvious motor function impairment of PD mice. Those results indicated the successful establishment of PD mice model. Then, to clarify its distribution and metabolic profile, PQR NPs were administrated into PD mice via tail vein injection. In vivo bioimaging results showed fluorescent signals appeared in the brains of PD mice at 3 h post‐injection, peaked at 9 h, and nearly disappeared by 72 h. Among main organs, the liver and kidneys showed the strongest fluorescence signals 12 h after administration, and the fluorescence signals were hardly detectable after 72 h (Figure 7C). These findings indicated that PQR NPs could effectively accumulate in the brain and were primarily metabolized via the liver and kidneys. Mice were treated with different groups, including control group, PD group, QC group, and PQR NPs group, and the intervention effects were evaluated via behavioral assessments as schematically illustrated in Figure 7D. Open‐field test results showed that PD mice exhibited significant reductions in total movement distance, speed, number of entries into the central zone, and distance stayed in the central zone, all of which were substantially improved after PQR NPs intervention (Figure 7E–G; Figure S44). In the rotarod test, the latency of drop off in PD mice was shortened, while PQR NPs intervention prolonged it significantly (Figure 7H). The pole test showed that the Total time (T‐total) and Turning time (T‐Turn) of PD mice were significantly increased, which were reversed by PQR NPs intervention(Figure 7I,J). Behavioral assessment results suggested that PQR NPs effectively ameliorated motor deficit of PD mice, which was superior to that of QC. Moreover, to visualize the capacity for monitoring intervention effect, PQR NPs were secondarily administrated into mice with different treatments. In vivo imaging results showed that the strongest fluorescent signal was observed in brain of PD mice, and the signal was weakened in the QC treated mice; while the weakest signal was observed in PQR NPs treated mice (Figure 7K). Furthermore, DFO, a well‐recognized iron chelator, was adopted to verify the ferroptosis alleviating effect in PD mice. As shown in Figure S45, behavioral test results demonstrated that both the DFO and PQR NPs treatment significantly ameliorated motor deficits in MPTP induced PD mice. Consistently, in vivo imaging results showed that the brain fluorescence signal derived from PQR NPs was significantly attenuated in those mice (Figure S46). To determine the brain region localization of the fluorescent signal of PQR NPs, we conducted confocal imaging on mouse brain frozen sections. As shown in Figure S47, SN of MPTP induced PD mice exhibited pronounced fluorescent signals compared with that of cerebral cortex. The fluorescence signal was substantially reduced by DFO or PQR NPs. Beyond that, the Fe^2+^ in SN and cerebral cortex of mice with different treatments was also assessed with commercial detection kit. As shown in Figure 7L, compared with that of cerebral cortex, the Fe^2^^+^ content in the SN of MPTP induced PD mice significantly increased, which was obviously reduced by DFO or PQR NPs intervention. Notably, no significant difference of Fe^2^^+^ content was observed in the cerebral cortex of mice among different treatment groups (Figures S48A). These findings proved that there was Fe^2^^+^ accumulation of SN of PD mice, which could be monitored by PQR NPs. To further verity the ferroptosis in PD mice, the LPO content in the SN and cerebral cortex of brain was also detected in strict accordance with the operating procedures provided in the manual. As shown in Figure 7M, the LPO level in the SN of PD mice increased significantly, and it was significantly reduced by DFO or PQR NPs treatment. No significant difference was observed in LPO level in the cerebral cortex of mice among different treatment groups (Figures S48B). These findings confirmed that ferroptosis occurred in SN of MPTP induce mice, and DFO or PQR NPs could ameliorate the ferroptosis. Above results indicated that PQR NPs hold promise to monitor the alteration of Fe^2+^ and viscosity in PD and assess the therapeutic efficacy of pharmacological intervention.

### 2.8 Rescue Mechanism of PQR NPs in PD Mice

**FIGURE 8:**
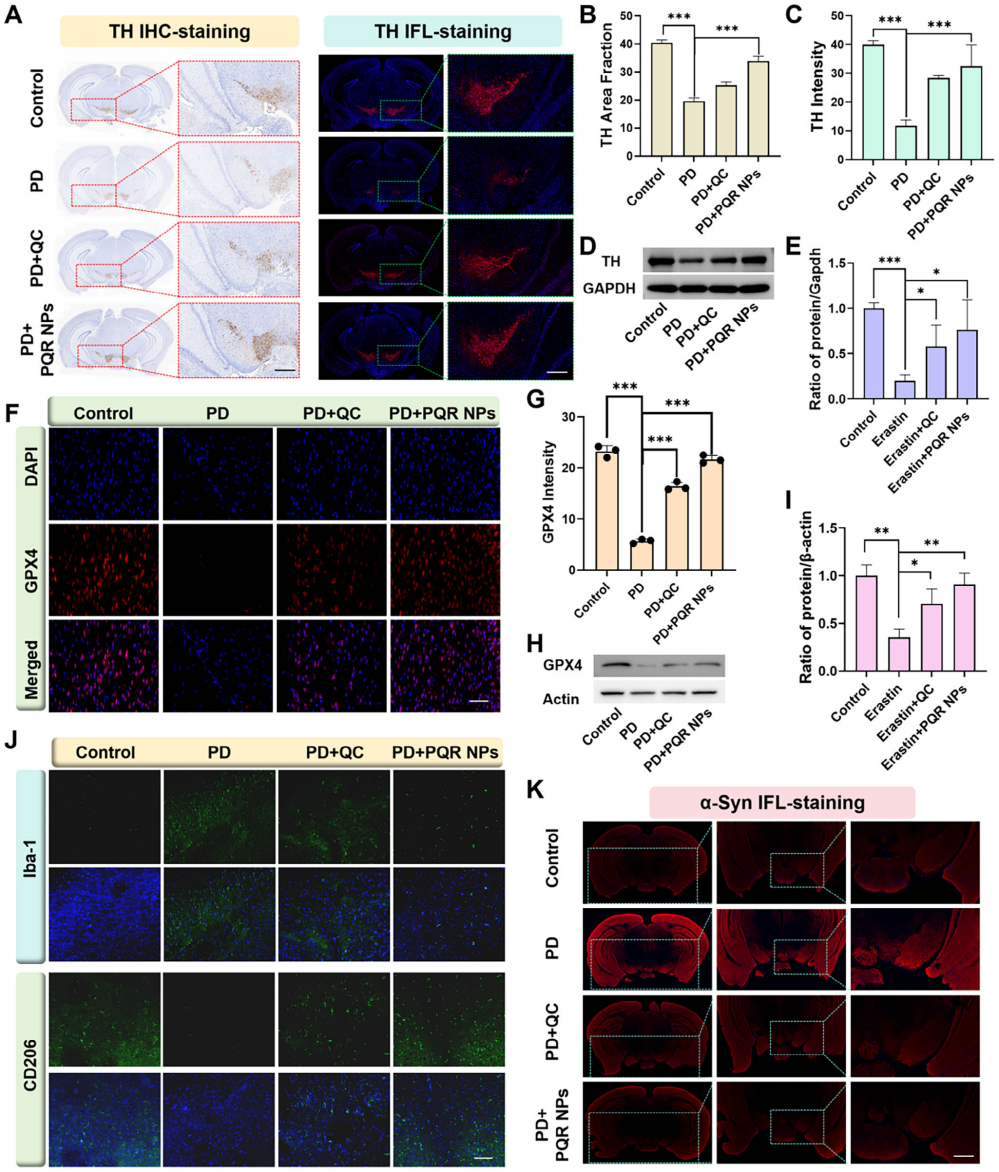
PQR NPs rescue mechanism on PD mice. (A) IHC and IFL staining images of TH in the SN of brain slices from mice with different treatment (Scale bars 500 µm). (B) Quantitative statistical analysis graph of IHC and (C) IFL of TH of brain slices. (D) WB image and (E) Quantitative statistical analysis graph of TH expression in brain. (F) IFL staining images and (G) Quantitative statistical analysis graph of GPX4 in brain slices from mice with different treatment (Scale bars 20 µm). (H) WB image and (I) Quantitative statistical analysis graph of GPX4 expression in mice brain. IFL staining images of (J) CD206 & Iba‐1, and (K) α‐ Syn in brain slice of mice with different treatment (Scale bars 100 µm CD206 & Iba‐1, 20 µm for α‐Syn). Data presented as mean ± SD, *n* = 3 for each, *ANOVA test*, *p < 0.05, **p < 0.01, and ***p < 0.001.

After observing the rescue effect of PQR NPs on PD mice, we wish to know its underlying mechanism. We carried out a series of cellular and molecular assay to determine the alteration of key proteins involved. As shown in Figure 8A–C, TH immunohistochemical (IHC) and IFL staining results demonstrated that PQR NPs significantly increased DA neuron counts, which was superior to than that of QC alone. DA neuron hallmark protein, TH, WB results also showed that PQR NPs treatment reversed reduction of TH expression induced by MPTP, which was more efficient than that of QC (Figure 8D,E). GPX4 was a featured protein that was down‐regulated in ferroptosis [52]. IFL results showed that GPX4 in PD brain slice was significantly reduced. PQR NPs or QC treatment elevated the expression of GPX4, with PQR NPs exhibiting superior effect (Figure 8F,G). WB assay further confirmed alteration pattern of GPX4 in mice with different treatment (Figure 8H,I). These results suggested that PQR NPs could effectively alleviate the ferroptosis in PD mice by up‐regulating GPX4 expression. Abnormal aggregation of α‐Syn was the core driver of PD pathogenesis, which could be induced by MPTP administration [53]. Moreover, microglia were unique resident immune cells in the brain, and polarization of microglia was strongly implicated in PD pathogenesis [54]. Ionized calcium‐binding adapter molecule 1 (Iba‐1) was the well‐recognized M1 polarization marker, whereas CD206 served as M2 polarization marker [55]. IFL results showed in the PD mice brain Iba‐1 was up‐regulated and CD206 was down‐regulated. PQR NPs administration reversed that pattern, up‐regulating CD206 and down‐regulating Iba‐1, indicating M2 polarization (Figure 8J). IFL results showed that PQR NPs effectively attenuated the aggregation of α‐Syn in the PD mice brain (Figure 8K; Figure S49). In summary, PQR NPs exert synergistic neuroprotective effects through multiple pathways, including up‐regulating GPX4, inhibiting α‐Syn aggregation, and inducting microglia M2 polarization, eventually rescuing DA neuron loss in PD mice. Finally, we systematically evaluated the biosafety of PQR NPs. The MTT assay showed that PQR NPs did not affect the viability of PC‐12 cell (Figure S50). The hemolysis test results showed that PQR NPs hardly induced hemolysis (Figure S51). Hematoxylin‐eosin (HE) staining of the main organs including heart, liver, spleen, lungs, and kidneys of mice with PQR NPs administration had no obvious change compared with that of control (Figure S52). Serum biochemical index, reflecting function of liver and kidney, such as aspartate transaminase (AST), alanine transaminase (ALT), blood urea nitrogen (BUN), and creatinine (CRE) showed no obvious fluctuation compared with control (Figure S53). Collectively, those results indicated that PQR NPs had good biosafety.

## 3 Conclusion

In this study, a Fe^2^^+^/Viscosity cascaded‐activated probes, NP1–5 was designed and synthesized, and their diagnostic and dynamic monitoring performance in PD was systematically verified through targeted therapeutic intervention. Among the series of probes, NP3 stood out, which exhibited Fe^2^^+^/Viscosity cascade activation properties, such as high selectivity, and excellent anti‐interference ability. It sensitively monitored Fe^2^^+^/Viscosity fluctuation in cellular, Drosophila and *C. elegans* PD models. To monitor Fe^2+^/Viscosity in in PD with ferroptosis intervention, an innovative nanoplatform, PQR NPs, was constructed by self‐assembly of NP3 and QC. In vitro and in vivo experiments demonstrated that PQR NPs not only alleviated ferroptosis‐induced loss of DA neurons by reducing oxidative stress and neuroinflammation, mitigating α‐synuclein aggregation, and restoring mitochondrial function, but also could detected the elevated Fe^2^^+^/Viscosity in PD models with ferroptosis targeted intervention. Notably, biosafety assessments showed that PQR NPs had good biocompatibility. Summarily, in present study we innovatively developed a cascade‐activated NIR probe which realized monitoring Fe^2^^+^/Viscosity in ferroptosis involved PD. It also paved ways for constructing precise theranostic strategies toward ferroptosis related diseases.

## 4 Experimental Section

### 4.1 Synthesis of NP1–5

Synthetic details were provided in the supporting information.

### 4.2 Measurement of Optical Property

For all optical property tests, the buffer was HEPES (pH 7.4) solution. The excitation slit and emission slit were set to 5 and 10 nm, respectively. The excitation and emission wavelengths for NP1–5were as follows: NP1 and NP5 (E_x_/E_m_ = 600/650 nm), NP2 and NP4 (E_x_/E_m_ = 610/650 nm), NP3 (E_x_/E_m_ = 590/650 nm).

#### 4.2.1 Spectral of NP1–5 in Response to Fe^2^^+^/Gly Test

Probes NP1–5 (10 µM), with Gly (10%, volume percentage), and Fe^2^^+^(100 µM). After at 37°C for 5 min, the solution was transferred to a cuvet for absorption and fluorescence spectral measurements.

#### 4.2.2 Stability of NP1–5 Test

Probes NP1–5 (10 µM) were transferred to a cuvet, and the changes in FI of different probes within 30 min were measured.

#### 4.2.3 Sensitivity of NP3 in Response to Fe^2^^+^, Gly, or Fe^2^^+^/Gly Test

Different concentrations of Fe^2^^+^ or different volume percentages of Gly with NP3 (10 µM), and then absorption and fluorescence spectral were performed at 37°C for 5 min. The concentrations of Fe^2^^+^ were 0, 5, 10, 20, 30, 40, 50, 60, 70, 80, 90, 100, 110, 120, 130, 140, and 150 µM, respectively. The volume percentages of Gly were 10%, 20%, 30%, 40%, 50%, 60%, 70%, 80%, and 90%, respectively. For the Fe^2+^/Gly, Gly(10%) with Fe^2+^ at different concentrations.

#### 4.2.4 Time‐Dependent of NP3 in Response to Fe^2^^+^, Gly, or Fe^2^^+^/Gly

Probe NP3 was used at a concentration of 10 µM, with Gly at 10% and Fe^2^^+^ at 100 µM. Fluorescence spectra were performed every 2.5 min at 37°C.

#### 4.2.5 Temperature and pH Stability of NP3 in Response to Fe^2^^+^/Gly

Probe NP3 was used at a concentration of 10 µM, with 10% Gly and 100 µM Fe^2^^+^. Fluorescence spectral were performed after reaction for 5 min under different temperatures or different pH, respectively. The temperatures were 25°C, 30°C, 35°C, 40°C, and 45°C, while the pH were 4.0, 5.0, 6.0, 7.0, 8.0, 9.0, and 10.0, respectively.

#### 4.2.6 Selectivity and Interference of NP3 in Response to the Fe^2^^+^/Gly

The concentration of NP3 was 10 µM. The relevant analytes tested including Mg^2^^+^ (1 mM), K^+^ (1 mM), Al^3^^+^ (1 mM), Fe^3^^+^ (1 mM), Pb^2^^+^ (1 mM), Cu^2^^+^ (1 mM), Mn^2^^+^ (1 mM), CN^−^ (100 µM), ONOO^−^ (100 µM), CH_2_O (100 µM), NO_3_^−^ (100 µM), SO_3_^2^^−^ (100 µM), HSO_3_^−^ (100 µM), GSH (100 µM), NO (100 µM), HS^−^ (100 µM), H_2_O_2_ (100 µM), Gly (10%), Fe^2^^+^ (100 µM), and Fe^2^^+^ (100 µM) + Gly (10%).

### 4.3 Cell Experiments

#### 4.3.1 MTT Assay

PC‐12 Cells were seeded into 96‐well plate at density of 1 × 10^4^ cells per well, and cultured in DMEM medium supplemented with 10% serum and 1% double antibodies (penicillin‐streptomycin). After the cells adhered to the plate, different concentrations NP3 was added. After incubated for 24 h, the culture medium was discarded. Then, 100 µL of MTT solution was added to each well, and the incubation was continued in the incubator for another 4 h. After that, the MTT solution was removed, and 150 µL of DMSO was added and measured absorbance at 490 nm by microplate reader.

#### 4.3.2 TH Expression Level Test

Cultured cells were treated with different groups, including the control group, the Erastin group, and the Erastin + Fer‐1 group. Cellular proteins were extracted by lysing cells on ice using RIPA lysis buffer (supplemented with 1% protease inhibitor PMSF). Subsequently, protein concentration was quantified by BCA kit. After protein denaturation, the samples were subjected to SDS‐PAGE gel electrophoresis, membrane transfer, blocking, and primary antibody incubation overnight (TH antibody, dilution ratio 1:1000). Following incubation of the samples with the secondary antibody (horseradish peroxidase‐labeled goat anti‐rabbit IgG, dilution ratio 1:5000) for 2 h, exposure and color development were performed.

#### 4.3.3 Cell Imaging

PC‐12 cells in the logarithmic growth phase were harvested and incubated in complete medium containing different concentrations of ferrous ions (0, 25, 50, and 100 µM) for 30 min to establish a ferrous ion‐only cellular model. The cells were subjected to 30 min of starvation treatment with serum‐free medium to establish a high‐viscosity cellular model. For the endogenous Fe^2^^+^/Viscosity model, Cultured cells were treated with different groups, including the control group, the NP3 group, the Erastin + NP3 group, and the Erastin + Fer‐1 + NP3 group and Erastin + OFO + NP3 group. Cells were treated with Erastin for 12 h, followed by incubation with Fer‐1 or DFO for another 12 h. The concentration of NP3 was 10 µM, Erastin and Fer‐1 were both 20 µM, DFO was 30 µg/mL. After successful establishment of the cellular model, cells were treated with NP3 (10 µM) for 30 min at 37°C. The detection protocol for intracellular LPO was implemented as following. After the successful establishment of the endogenous Fe^2^^+^/viscosity cellular model, commercial LPO probe BODIPY 581/591 C11 (10 µM) was added, followed by co‐incubation at 37°C for 30 min. Then washed with PBS, fixed with 4% paraformaldehyde, stained with DAPI for 20 min, washed three times with PBS, and finally subjected to confocal imaging analysis under CLSM. The confocal imaging parameters for each indicator were configured as follows. The excitation/emission wavelength (λex/λem) of DAPI was 405/430–470 nm; that of NP3 was 561/570–670 nm; for LPO detection, the λex/λem of the green channel was 488/500–540 nm, while that of the red channel was 561/570–620 nm.

#### 4.3.4 MDA Test

After the successful establishment of the endogenous Fe^2^^+^/viscosity cellular model, the MDA assay kit was used, and the indicator determination was completed in strict accordance with the kit's operating instructions.

### 4.4 Imaging of Drosophila

#### 4.4.1 Drosophila Culture

Drosophila were divided into two types: recessive mutant (parkin^−^/^−^) Parkin‐null Drosophila and wild‐type (WT) Drosophila. The Drosophila strains were obtained from the Neurodegeneration Research Laboratory (NDRL) of the National Neuroscience Institute, Singapore. Drosophila were cultured in CO_2_‐containing culture vials under constant temperature and humidity conditions, with the optimal temperature maintained at 25°C and relative humidity at approximately 60%. The culture medium was prepared using raw materials including corn flour, soybean flour, sucrose, maltose, high‐active dry yeast, and agar powder.

#### 4.4.2 Construction and Characterization of the Drosophila PD Model

WT Drosophila were treated with different groups, including the control group, the Erastin group, and the Erastin + Fer‐1 group. When the different treatments were completed, Drosophila were transferred into graduated tubes for behavioral statistics analysis.

#### 4.4.3 DA Neuron Imaging

Brain tissues were dissected from the cultured Drosophila with different treatment and incubated with the TH antibody. IFL imaging of DA neurons was performed under CLSM.

#### 4.4.4 Drosophila Brain Imaging

Drosophila were treated with different groups, including WT Drosophila group, WT Drosophila + Erastin group, WT Drosophila + Erastin + Fer‐1 group, PD Drosophila group. Brain tissues were dissected from the cultured Drosophila with different treatment, then co‐incubated with PBS solution containing NP3 for 30 min. Fluorescence imaging was conducted under CLSM.

### 4.5 Imaging of *C. elegans*

#### 4.5.1 *C. elegans* Culture

*C. elegans* strains were obtained from the NDRL of the National Neuroscience Institute, Singapore. It was cultured on Nematode Growth Medium (NGM) agar plates. The growth status of *C. elegans* was observed every other day, and they were seeded with the *Escherichia coli* (*E. coli*) strain OP50 at 20°C.

#### 4.5.2 Construction and Characterization of the *C. elegans* PD Model

WLZ1 *C. elegans* were treated with different groups, including the control group, the Erastin group, and the Erastin + Fer‐1 group. When the different treatments were completed, images of *C. elegans* were recorded for 20 s using microscope. The number of body bends was counted and analyzed using Image J.

#### 4.5.3 DA Neuron IFL Imaging

*C. elegans* were treated with different groups, including Erastin, and Erastin+Fer‐1. After different treatment, *C. elegans* were fixed with 4% paraformaldehyde, permeabilized with 0.1% Triton X‐100 for 10 min, and washed with PBS. Following the samples were blocked with 5% BSA for 1 h, added the diluted TH primary antibody, and incubated overnight at 4°C. After washing with PBS, secondary antibody was added for 1 h, followed by washes with PBS. Finally, *C. elegans* were mounted with anti‐fluorescence quenching agent and observed and imaged under CLSM.

#### 4.5.4 *C. elegans* Imaging

*C. elegans* were treated with different groups, including WLZ1 *C. elegans* group, WLZ1 *C. elegans* + Erastin group, WLZ1 *C. elegans* + Erastin + Fer‐1 group, PD *C. elegans* (WLZ3) group. After different treatment, *C. elegans* were co‐incubated with NP3 for 5 min, followed by imaging under CLSM.

### 4.6 Preparation and Characterization of PQR NPs

#### 4.6.1 Preparation of PQR NPs

Organic solvents and ultrapure water were treated with a 0.22 µm filter membrane. Poly(lactic‐co‐glycolic acid)—Polyethylene glycol—RVG 29 (PLGA‐PEG‐RVG29) (10 mg), NP3 (3.0 mg), QC (3.0 mg) were dissolved in tetrahydrofuran (THF). The mixture was then added to ultrapure water under ultrasonic conditions, sonicated for 3 min, and stirred at room temperature for 24 h. Finally, THF was removed by nitrogen bubbling, and the precipitate was collected by centrifugation and freeze‐dried to obtain PQR NPs.

#### 4.6.2 Morphology and structure

The morphology and structure of PQR NPs were characterized by TEM, DLS, and absorption spectrometer.

#### 4.6.3 Optical Performance Test

The absorption and fluorescence spectra of PQR NPs were determined using the same method as for probe NP3. The concentration of PQR NPs was 50 µg/mL, the temperature was 37°C, and the time was 60 min.

#### 4.6.4 ABTS Free Radical Scavenging Test

ABTS (7.4 mM) and K_2_S_2_O_8_ (2.6 mM) stock solution were prepared and mixed. After standing in the dark at room temperature for 12 h, the mixture was diluted 50‐fold with EtOH to obtain an ABTS^+^• radical working solution, which was stored at 4°C. Different concentrations of PQR NPs were added to the diluted ABTS. After the reaction was completed, the color change was recorded, and the absorbance at 734 nm was measured by spectrophotometer. The calculation formula for scavenging rate was as follows:

$$\mathrm{Scavenging}\;\mathrm{rate}\left(\%\right)=\left[\left(A_{0}-A\right)/A_{0}\right]\times 100\%$$

where A_0_ and A were the absorbance value of ABTS without or with PQR NPs, respectively.

### 4.7 Cellular Experimental of PQR NPs

#### 4.7.1 Cellular Uptake and Imaging

After cells were cultured, it were co‐incubated with culture medium containing PQR NPs (50 µg/mL). After different time points (0, 1, 2, 3, and 4 h), the cells were washed with PBS and fixed with 4% paraformaldehyde for 20 min. Then, 4',6‐diamidino‐2‐phenylindole (DAPI) was added for co‐incubation in the dark for 30 min, fluorescence imaging was performed using CLSM. The method for cellular imaging of PQR NPs was consistent with that of NP3.

#### 4.7.2 PQR NPs Against Rot‐ or Erastin Induced Cellular Damage Test

Cells were seeded into 96‐well plates at a density of 1 × 10^4^ cells/well. After adherence, culture media containing different concentrations of Rot or Erastin were added respectively, and co‐incubation for 12 h. MTT solution was then added, followed by further incubation in the incubator for 4 h. After discarding the MTT solution, DMSO was added for co‐incubation for 30 min, and the absorbance at 490 nm was measured using microplate reader. Finally, the concentrations of Rot or Erastin that resulted in 50% cell viability were selected as the concentrations for constructing the cellular PD model. After the successful establishment of the cellular PD model, culture media containing different concentrations of PQR NPs were added for 12 h. Subsequently, MTT solution and DMSO were added, and the absorbance at 490 nm was measured using microplate reader. The concentrations of PQR NPs were 0, 10, 20, 30, 40, 50, and 60 µg/mL, respectively. The concentrations of Rot were 0, 1, 2, 3, 4, and 5 µM, respectively. The concentrations of Erastin were 0, 15, 30, 45, 60, and 75 µM, respectively. The cell viability was calculated as follows:

$$\mathrm{CR}=\left(A/A_{0}\times 100\%\right)$$

where A and A_0_ are the absorbance values of PC‐12 cells treated or untreated with PQR NPs, respectively.

#### 4.7.3 AM/PI Staining

Cultured cells were treated with different groups, including the control group, the Rot (or Erastin) group, the Rot (or Erastin) + QC group, and the Rot (or Erastin) + PQR NPs group. After the cells were treated with different groups, they were washed with PBS, and AM‐PI stock solution was added for 30 min. Imaging was performed using fluorescence microscope.

#### 4.7.4 ATP Test

Cell grouping and preparation were consistent with the experimental methods described above. After the cells were treated with different groups, cell lysis buffer was added to each well of the 6‐well plate. After lysis, centrifugation was performed at 12 000 rpm for 5 min. The supernatant was collected and used for ATP content detection with ATP assay kit.

#### 4.7.5 JC‐10 Staining

Cell grouping and preparation were consistent with the experimental methods described above. After the cells were treated with different groups, JC‐10 working solution was added for 20 min. Subsequently, the cells were washed with PBS, and paraformaldehyde was added for 15 min. After the treatment, imaging was performed using CLSM.

#### 4.7.6 Intracellular ROS Imaging

Cell grouping and preparation were consistent with the experimental methods described above. After the cells were treated with different groups, DCFH‐DA probe was added for 30 min. Then, the cells were washed with PBS, and observation and imaging were conducted using fluorescence microscope. Meanwhile, the cells in different groups were also detected using flow cytometer.

#### 4.7.7 In Vitro BBB Model Penetration Assay of PQR NPs

To establish an in vitro BBB model, bEnd.3 cells were seeded into upper chamber of Transwell inserts at a density of 5 × 10^4^ cells/mL, and PC‐12 cells were seeded in the lower chamber, followed culture for 7 days. The trans‐epithelial electrical resistance (TEER) was measured using Beijing JINGONG RE1600 epithelial volt‐ohmmeter. When the TEER value reached 200 Ω·cm^2^, the in vitro BBB model was considered established. To evaluate the penetration capacity of PQR NPs, equal concentrations of NP3 and PQR NPs were added to the upper chamber. After 12 h, PC‐12 cells in the lower chamber were collected, treated with Erastin, and imaged using a laser confocal microscope.

#### 4.7.8 TH and GPX4 Expression Level Test

Cell grouping and preparation were consistent with the experimental methods described above. After the cells were treated with different groups, RIPA lysis buffer (supplemented as required) was added for lysis to obtain cellular proteins, and protein concentration was determined by BCA kit. Subsequently, the proteins were denatured at high temperature, followed by SDS‐PAGE gel electrophoresis, membrane transfer, blocking, and overnight co‐incubation with primary antibodies (TH antibody, GPX4 antibody, dilution ratio 1:1000). After co‐incubation with the secondary antibody (horseradish peroxidase‐labeled goat anti‐rabbit IgG, dilution ratio 1:5000) for2 h, exposure and color development were performed.

#### 4.7.9 α‐Syn IFL Staining

PC‐12 cells were treated with different groups, including control group, Erastin group, and Erastin combined with PQR NPs group. Subsequently, the cells were fixed with 4% paraformaldehyde, permeabilized with 0.1% Triton X‐100 for 10 min at room temperature, and washed 3 times with PBS. Following blocking with 5% BSA for 1 h, diluted α‐Syn primary antibody was added, and the cells were incubated overnight at 4°C. After washing with PBS, Cy3 labeled secondary antibody was added for 1 h incubation in the dark, followed by washes with PBS. Finally, the cells were mounted with anti‐fluorescence quenching agent and observed and imaged under fluorescence microscope.

#### 4.7.10 qPCR Assay

Total RNA was extracted from PC‐12 cells using RNAiso Plus (Takara, Shiga, Japan, Cat# 9109). Complementary DNA (cDNA) was synthesized using an RT‐reagent kit (Takara, Shiga, Japan, Cat# RR047A) followed by the assessment of gene expression using TB Green PCR reagent system (Takara, Shiga, Japan, Cat# RR820A), following the manufacturer's protocol. Primers pair sequences are shown in Table S1).

#### 4.7.11 Transcriptome Assay

Cell grouping and preparation were consistent with the experimental methods described above. After the cells were treated with different groups, they were sent to Baiqu Biotechnology Co., Ltd. for analysis.

### 4.8 Animal Experimental of PQR NPs

#### 4.8.1 Establishment of MPTP Induced Mice PD Model, Behavioral Tests, and TH IFL Staining

C57 mice aged 4–6 weeks were purchased from Beijing Jieman Biotechnology Co., Ltd. All animal experiments were conducted in accordance with the Regulations on the Administration of Experimental Animals of the People's Republic of China and approved by the Animal Ethics and Use Committee of Shanxi Medical University (Approval No.: IACU 2017–018). Experimental animals were randomly assigned to establish MPTP induced PD mice. The mice PD model was established by intraperitoneal injection of MPTP at a dose of 25 mg/kg, once daily for 7 consecutive days. Subsequently, PD model mice and drug‐intervened PD model mice were grouped separately, and the group allocation information was subjected to blinding treatment. Behavioral tests of mice included the open field test, pole test, and rotarod test. All behavioural experimental procedures and data analyses were conducted using a double‐blind manner. In addition, mice brain tissues were isolated for TH IFL staining, and imaged by fluorescence microscope.

#### 4.8.2 Small Animal In Vivo Imaging

The brains of MPTP induced mice PD models were depilated. PQR NPs were injected via the tail vein, and fluorescence imaging of the mice brain and the metabolism of various organs was observed at different time points by small animal in vivo imaging system. The wavelength of excitation filter was 600 nm, and emission filter was 670 nm.

#### 4.8.3 Behavioral Assessment

Mice were treated with different groups, including the control group, the MPTP group, the MPTP + QC group, the MPTP + PQR NPs group. Equal doses of QC and PQR NPs were injected via the tail vein on the third day before MPTP injection, the third day of MPTP injection, the sixth day of MPTP injection, and the second day after the end of MPTP injection, respectively. To investigate whether ferroptosis occurs in the MPTP induced PD mice model, PD mice and drug intervention groups treated with PQR NPs and DFO were reconstructed. DFO were administered via the intranasal route, and the administration time points were implemented in accordance with the aforementioned procedures. After the experiment, the behavioral assessment of mice in different groups was conducted using the open field test, pole test, and rotarod test.

#### 4.8.4 Fe^2^^+^ and LPO Assays with Brain Tissue

Following completion of annimal experiments (Control Group, MPTP induced PD Group, PQR NPs‐intervened PD Group, and DFO‐intervened PD Group). Following completion of animals experiments, brain tissue was isolated, added to extraction solution, and subjected to cryogenic homogenisation. Subsequent measurements were strictly conducted according to the operational protocols outlined in the commercial LPO and Fe^2^^+^ detection kits.

#### 4.8.5 Brain Tissue Staining

Upon completion of annimal experiments (Control Group, MPTP induced PD Group, PQR NPs‐intervened PD Group, and DFO‐intervened PD Group). Upon completion of animal experiments, brain tissues from mice in each group were isolated and processed into frozen sections. Subsequently, 50 µL of probe solution (20 µM) was added onto the target brain regions. The sections were placed in a humidified chamber and incubated at 37°C for 30 min, washed with PBS, and finally subjected to confocal imaging analysis under CLSM. The confocal imaging parameters for each indicator were configured as follows. The excitation/emission wavelength (λex/λem) of NP3 was 561/570–670 nm.

#### 4.8.6 IFL Staining

After the mice experiment, the heart, liver, spleen, lung, kidney, and brain of the mice were removed using sterile scissors and forceps. Fixative was immediately added to the excised tissues, which were then embedded in paraffin blocks. The SN striatum was selected as the target region for sectioning. The sections were incubated at room temperature for blocking for 40 min. After blocking, the sections were directly placed into a TH primary antibody mixture diluted with antibody dilution buffer and incubated overnight at 4°C. The sections incubated with the TH primary antibody were washed with PBS, then transferred to a secondary antibody mixture and incubated at room temperature for 2.5 h in the dark. The sections were mounted with a mounting medium containing DAPI and imaged by fluorescence microscope. The IFL staining procedures for GPX4, CD206, Iba‐1, and α‐Syn were identical to those described above.

#### 4.8.7 HE Staining

Sections of the heart, liver, spleen, lung, and kidney were sequentially placed in preheated xylene to remove paraffin, followed by sequential immersion in absolute ethanol (repeated three times), 95% ethanol, 70% ethanol, and ultrapure water, with each step lasting 5 min. Finally, the samples were stained with HE. After dehydration and mounting, the sections were imaged under microscope.

#### 4.8.8 Statistical Analysis

During data pre‐processing, outliers were identified and removed to reduce experimental errors’ interference with results. Data were expressed as mean ± standard deviation (SD). The sample size (n) for each statistical analysis was no less than three. Student t‐test and one‐way ANOVA were used for intergroup comparisons by Graphpad Prism 9.5 software. The statistical difference analyses were defined as *p < 0.05, **p < 0.01, and ***p < 0.001, respectively.

## Funding

The work is supported by National Natural Science Foundation of China (No: 82472030), Shanxi Scholarship Council of China (2024‐079), Shanxi Provincial Doctoral Fund Project (No. SD2020), Shanxi Medical University Doctoral Fund Project (NO. XD2030). The China Postdoctoral Science Foundation (No. 2023M732141).

## Conflicts of Interest

The authors declare no conflict of interest.

## Supporting information




**Supporting File**: advs74586‐sup‐0001‐SuppMat.docm.

## Data Availability

The data that support the findings of this study are available from the corresponding author upon reasonable request.

## References

[1] T. Kurth and R. Brinks , “Projecting Parkinson's Disease Burden,” Bmj 388 (2025): r350, 10.1136/bmj.r350. 40044228

[2] L. Xu , Z. Wang , and Q. Li , “Global Trends and Projections of Parkinson's Disease Incidence: a 30‐year Analysis Using GBD 2021 Data,” Journal of Neurology 272 (2025): 286, 10.1007/s00415-025-13030-2. 40131471

[3] M. Hodaie , J. S. Neimat , and A. M. Lozano , “The Dopaminergic Nigrostriatal Systemand Parkinson's Disease,” Neurosurgery 60 (2007): 17–30, 10.1227/01.NEU.0000249209.11967.CB. 17228250

[4] B. Garcia Santa Cruz , A. Husch , and F. Hertel , “Machine learning models for diagnosis and prognosis of Parkinson's disease using brain imaging: General overview, main challenges, and future directions,” Frontiers in Aging Neuroscience 15 (2023): 1216163, 10.3389/fnagi.2023.1216163. 37539346 PMC10394631

[5] A. A. Vijayakumari , H. H. Fernandez , and B. L. Walter , “MRI‐based multivariate gray matter volumetric distance for predicting motor symptom progression in Parkinson's disease,” Scientific Reports 13 (2023): 17704, 10.1038/s41598-023-44322-0. 37848592 PMC10582255

[6] M. Politis and P. Piccini , “Positron Emission Tomography Imaging in Neurological Disorders,” Journal of neurology 259 (2012): 1769–1780, 10.1007/s00415-012-6428-3. 22297461

[7] O. Hansson , “Biomarkers for Neurodegenerative Diseases,” Nature Medicine 27 (2021): 954–96310, 10.1038/s41591-021-01382-x. 34083813

[8] L. Guo , X. Li , S. Xie , et al., “Double‐locked probe for NIRF/PA imaging mitochondrial H2O2 and viscosity in Parkinson's disease,” Sensors and Actuators B: Chemical 426 (2025): 137104, 10.1016/j.snb.2024.137104.

[9] H. Dong , L. Zheng , Z. Wang , et al., “Dual‐mode ratiometric electrochemical and turn‐on fluorescent probe for reliably detecting H2O2 in Parkinson's disease serum,” Sensors and Actuators Reports 9 (2025): 100305, 10.1016/j.snr.2025.100305.

[10] T. Liu , X. Han , X. Sun , et al., “An activated fluorescent probe to monitor NO fluctuation in Parkinson's disease,” Chinese Chemical Letters 36 (2025): 110170, 10.1016/j.cclet.2024.110170.

[11] K. Wu , X. Wang , L. Gong , et al., “Screening of H2S donors With a red emission mitochondria‐targetable fluorescent probe: Toward discovering a new therapeutic strategy for Parkinson's disease,” Biosensors and Bioelectronics 237 (2023): 115521, 10.1016/j.bios.2023.115521. 37429146

[12] X. Sun , Q. Jiang , Y. Zhang , et al., “Advances in Fluorescent Probe Development for Bioimaging of Potential Parkinson's Biomarkers,” European Journal of Medicinal Chemistry 267 (2024): 116195, 10.1016/j.ejmech.2024.116195. 38330868

[13] C. Ma , W. Sun , L. Xu , et al., “A Minireview of Viscosity‐Sensitive Fluorescent Probes: Design and Biological Applications,” Journal Of Materials Chemistry B 8 (2020): 9642–9651, 10.1039/D0TB01146K. 32986068

[14] B. Xiong , Y. Wang , Y. Chen , et al., “Strategies for Structural Modification of Small Molecules to Improve Blood–Brain Barrier Penetration: A Recent Perspective,” Journal of Medicinal Chemistry 64 (2021): 13152–13173, 10.1021/acs.jmedchem.1c00910. 34505508

[15] L. Wu , J. Huang , K. Pu , and T. D. James , “Dual‐Locked Spectroscopic Probes for Sensing and Therapy,” Nature Reviews Chemistry 5 (2021): 406–421, 10.1038/s41570-021-00277-2. 37118027

[16] Z. L. Wang , L. Yuan , W. Li , et al., “Ferroptosis in Parkinson's Disease: Glia‐Neuron Crosstalk,” Trends in Molecular Medicine 28 (2022): 258–269, 10.1016/j.molmed.2022.02.003. 35260343

[17] D. Jiao , Y. Yang , K. Wang , and Y. Wang , “Ferroptosis: a Novel Pathogenesis and Therapeutic Strategies for Parkinson Disease: a Review,” Medicine 104 (2025): 41218, 10.1097/MD.0000000000041218. PMC11749581 39833092

[18] S. K. Ryan , M. Zelic , Y. Han , et al., “Microglia Ferroptosis Is Regulated by SEC24B and Contributes to Neurodegeneration,” Nature Neuroscience 26 (2023): 12–26, 10.1038/s41593-022-01221-3. 36536241 PMC9829540

[19] B. Dong , S. Li , Y. Wang , et al., “Recent Advance in the Development of the Fluorescent Responsive Probes for the Study of Ferroptosis,” TrAC Trends in Analytical Chemistry 168 (2023): 117327, 10.1016/j.trac.2023.117327.

[20] Y.‐L. Qi , H.‐R. Wang , L.‐L. Chen , Y.‐T. Duan , S.‐Y. Yang , and H.‐L. Zhu , “Recent Advances in Small‐Molecule Fluorescent Probes for Studying Ferroptosis,” Chemical Society Reviews 51 (2022): 7752–7778, 10.1039/D1CS01167G. 36052828

[21] X. Zeng , H. An , F. Yu , et al., “Benefits of Iron Chelators in the Treatment of Parkinson's Disease,” Neurochemical Research 46 (2021): 1239–1251, 10.1007/s11064-021-03262-9. 33646533 PMC8053182

[22] J. Sian‐Hulsmann and P. Riederer , “The Role of Alpha‐Synuclein as Ferrireductase in Neurodegeneration Associated With Parkinson's Disease,” Journal of Neural transmission 127 (2020): 749–754, 10.1007/s00702-020-02192-0. 32318880

[23] R. Cheng , V. V. Dhorajia , J. Kim , and Y. Kim , “Mitochondrial Iron Metabolism and Neurodegenerative Diseases,” Neurotoxicology 88 (2022): 88–101, 10.1016/j.neuro.2021.11.003. 34748789 PMC8748425

[24] H. Yu , Q. Chang , T. Sun , et al., “Metabolic Reprogramming and Polarization of Microglia in Parkinson's Disease: Role of Inflammasome and Iron,” Ageing Research Reviews 90 (2023): 102032, 10.1016/j.arr.2023.102032. 37572760

[25] N. Pareek , S. Mendiratta , N. Kalita , S. Sivaramakrishnan , R. S. Khan , and A. Samanta , “Unraveling Ferroptosis Mechanisms: Tracking Cellular Viscosity With Small Molecular Fluorescent Probes,” Chemistry—An Asian Journal 19 (2024), 10.1002/asia.202400056. 38430218

[26] L. Guo , X. Li , R. Zhang , et al., “In Situ Dual‐Activated NIRF/PA Carrier‐Free Nanoprobe for Diagnosis and Treatment of Parkinson's Disease,” Biosensors and Bioelectronics 282 (2025): 117473, 10.1016/j.bios.2025.117473. 40267542

[27] N. Sachan , S. Srikrishna , D. K. Patel , and M. P. Singh , “Deferoxamine Ameliorates Cypermethrin‐Induced Iron Accumulation and Associated Alterations,” Molecular Neurobiology 61 (2024): 4178–4187, 10.1007/s12035-023-03827-5. 38064103

[28] Q. Zheng , D. Huang , L. Zhao , et al., “Groundbreaking Insights Into SIRT1 / NRF2 ‐Mediated Ferroptosis Inhibition by Resveratrol in Parkinson's Disease Models,” CNS Neuroscience & Therapeutics 31 (2025): 70648, 10.1111/cns.70648. PMC12605972 41220093

[29] Z.‐H. Lin , Y. Liu , N.‐J. Xue , et al., “Quercetin Protects Against MPP + /MPTP‐Induced Dopaminergic Neuron Death in Parkinson's Disease by Inhibiting Ferroptosis,” Oxidative Medicine and Cellular Longevity 2022 (2022): 7769355, 10.1155/2022/7769355. 36105483 PMC9467739

[30] M. D. S. Islam , C. Quispe , R. Hossain , et al., “Neuropharmacological Effects of Quercetin: a Literature‐Based Review,” Frontiers in Pharmacology 12 (2021): 665031, 10.3389/fphar.2021.665031. 34220504 PMC8248808

[31] Y. Jiang , G. Xie , A. Alimujiang , et al., “Protective Effects of Querectin Against MPP+‐Induced Dopaminergic Neurons Injury via the Nrf2 Signaling Pathway,” Frontiers in Bioscience‐Landmark 28 (2023): 42, 10.31083/j.fbl2803042. 37005755

[32] A. Bardestani , S. Ebrahimpour , A. Esmaeili , and A. Esmaeili , “Quercetin Attenuates Neurotoxicity Induced by Iron Oxide Nanoparticles,” Journal of Nanobiotechnology 19 (2021): 327, 10.1186/s12951-021-01059-0. 34663344 PMC8522232

[33] I. Debnath , S. Ghosh , S. K. Jha , et al., “Mechanistic Insights and Therapeutic Potential of Quercetin in Neuroprotection: a Comprehensive Review of Pathways and Clinical Perspectives,” BIO Integration 6 (2025): 1–31, 10.15212/bioi-2025-0073.

[34] E. Lee , D. Yang , and J. H. Hong , “Prominent Naturally Derived Oxidative‐Stress‐Targeting Drugs and Their Applications in Cancer Treatment,” Antioxidants 14 (2025): 49, 10.3390/antiox14010049. 39857383 PMC11760868

[35] S. Zhu , Y. Zhang , C. Li , et al., “Multiple Synergistic Anti‐aging Effects of Vascular Cell Adhesion Molecule 1 Functionalized Nanoplatform to Improve Age‐related Neurodegenerative Diseases,” Journal of Controlled Release 379 (2025): 363–376, 10.1016/j.jconrel.2025.01.022. 39798706

[36] D. Zhao , C. Tian , M. Cheng , et al., “Carrier‐free Quercetin Nanomedicine Blocks NLRP3 Deubiquitination and TXNIP Recruitment for Parkinson's Disease Therapy,” Chemical Engineering Journal 464 (2023): 142697, 10.1016/j.cej.2023.142697.

[37] Y. Wu , C. Yin , W. Zhang , Y. Zhang , and F. Huo , “Mitochondrial‐Targeting Near‐Infrared Fluorescent Probe for Visualizing Viscosity in Drug‐Induced Cells and a Fatty Liver Mouse Model,” Analytical Chemistry 94 (2022): 5069–5074, 10.1021/acs.analchem.1c05288. 35286070

[38] Y. Lu , G. Ruan , W. Du , et al., “Recent Progress in Rational Design of Fluorescent Probes for Fe2+ and Bioapplication,” Dyes and Pigments 190 (2021): 109337, 10.1016/j.dyepig.2021.109337.

[39] Y. Li , F. Guo , R. Suo , et al., “A Caged Luciferin Analogue Generating Near‐infrared Bioluminescence for Activity‐sensing of Labile Iron,” Biosensors and Bioelectronics 278 (2025): 117290, 10.1016/j.bios.2025.117290. 40020638

[40] C. Xu , H. Zou , Z. Zhao , et al., “A New Strategy Toward “Simple” Water‐Soluble AIE Probes for Hypoxia Detection,” Advanced Functional Materials 29 (2019): 1903278, 10.1002/adfm.201903278.

[41] G. Feng , G. Q. Zhang , and D. Ding , “Design of Superior Phototheranostic Agents Guided by Jablonski Diagrams,” Chemical Society Reviews 49 (2020): 8179–8234, 10.1039/D0CS00671H. 33196726

[42] S. Prasad and D. P. Ojha , “Theoretical and Experimental Studies on Novel Liquid Crystals‐The Role of Position of Oxygen,” Journal of Materials Science 7 (2017): 35–40, 10.5923/j.materials.20170702.02.

[43] F. Naz , C. Si , S. Kellici , M. T. Sajjad , and E. Zysman‐Colman , “Red and Near‐Infrared Thermally Activated Delayed Fluorescence Emitters Based on a Dibenzo[f,h]pyrido[2,3‐b]quinoxaline Acceptor,” Chemistry—An Asian Journal 20 (2025): 202500136, 10.1002/asia.202500136. 40000407

[44] H. Li , J. Han , H. Zhao , et al., “Lighting up the Invisible Twisted Intramolecular Charge Transfer State by High Pressure,” The Journal of Physical Chemistry Letters 10 (2019): 748–753, 10.1021/acs.jpclett.9b00026. 30704239

[45] X. An , W. Zhang , X. He , M. Li , C. Rong , and S. Liu , “Density‐based Reactivity Theory Applied to Excited States,” AAPPS Bulletin 34 (2024): 8, 10.1007/s43673-023-00114-2.

[46] S. J. Dixon , K. M. Lemberg , M. R. Lamprecht , et al., “Ferroptosis: An Iron‐Dependent Form of Nonapoptotic Cell Death,” Cell 149 (2012): 1060–1072, 10.1016/j.cell.2012.03.042. 22632970 PMC3367386

[47] J. Liu , X. Xu , P. Xie , X. Yang , Y. Ye , and Y. Zhao , “A Novel Activatable Fluorescent Probe for Revealing the Dynamic of Esterase and Viscosity during Ferroptosis and DILI,” Sensors and Actuators B: Chemical 396 (2023): 134594, 10.1016/j.snb.2023.134594.

[48] D. Xu , M.‐J. Hu , Y.‐Q. Wang , and Y.‐L. Cui , “Antioxidant Activities of Quercetin and Its Complexes for Medicinal Application,” Molecules (Basel, Switzerland) 24 (2019): 1123, 10.3390/molecules24061123. 30901869 PMC6470739

[49] J. Khatun , J. D. Gelles , and J. E. Chipuk , “Dynamic Death Decisions: How Mitochondrial Dynamics Shape Cellular Commitment to Apoptosis and Ferroptosis,” Developmental Cell 59 (2024): 2549–2565, 10.1016/j.devcel.2024.09.004. 39378840 PMC11469553

[50] Z. Lv , Y. Zeng , T. Lv , Q. Liu , and L. Han , “GRP94 Mediates Blood‐brain Barrier Permeation and Substantia Nigra‐specific Drug Distribution in Parkinson's Disease,” Colloids and Surfaces B: Biointerfaces 250 (2025): 114585, 10.1016/j.colsurfb.2025.114585. 39983452

[51] M. Xiu , Y. Liu , Z. Wang , et al., “Abnormal Iron Metabolism in the Zona Incerta in Parkinson's Disease Mice,” Journal of Neural Transmission 132 (2025): 845–857, 10.1007/s00702-025-02913-3. 40119221

[52] K. Wang , X.‐Z. Chen , Y.‐H. Wang , et al., “Emerging Roles of Ferroptosis in Cardiovascular Diseases,” Cell Death Discovery 8 (2022): 394, 10.1038/s41420-022-01183-2. 36127318 PMC9488879

[53] G. Bellini , V. D'Antongiovanni , G. Palermo , et al., “α‐Synuclein in Parkinson's Disease: From Bench to Bedside,” Medicinal Research Reviews 45 (2025): 909–946, 10.1002/med.22091. 39704040 PMC11976381

[54] J. A. Kabba , Y. Xu , H. Christian , et al., “Microglia: Housekeeper of the Central Nervous System,” Cellular and Molecular Neurobiology 38 (2018): 53–71, 10.1007/s10571-017-0504-2. 28534246 PMC11481884

[55] S. Feng , C. Wu , P. Zou , et al., “High‐intensity Interval Training Ameliorates Alzheimer's Disease‐Like Pathology by Regulating Astrocyte Phenotype‐Associated AQP4 Polarization,” Theranostics 13 (2023): 3434–3450, 10.7150/thno.81951. 37351177 PMC10283053

