# Supplementary material for: Molecularly Engineered Phenoxazinone‐Skeleton Cascade‐Activated NIR Probes for Monitoring Fe2+/Viscosity in Ferroptosis‐Mediated Parkinson's Disease
Source: Adv Sci (Weinh). 2026 Mar 1;13(26):e24057. doi: 10.1002/advs.202524057 (PMC13159097; doi:10.1002/advs.202524057)
Supplement: Supplementary file 1 — Supporting File: advs74586‐sup‐0001‐SuppMat.docm. [file ADVS-13-e24057-s001.docm]

Supporting Information

**Molecularly Engineered Phenoxazinone-Skeleton Cascade-Activated NIR Probes for Monitoring Fe^2+^/Viscosity in Ferroptosis-Mediated Parkinson's Disease**

Lixia Guo(a,b), Tao Liu(c), Hongfei Wang(b), Qi Gao(b), Zumeng Wang(b), Zhaobo Yang(b), Chenlong Li(b), Yubing Kang(b), Kahleong Lim(d), Wen Liu(b)*, Li Lu(b)*, Chengwu Zhang(b)*

L.X. Guo

Third Hospital of Shanxi Medical University, Shanxi Bethune Hospital, Taiyuan 030032, P.R. China

School of Basic Medical Sciences, Shanxi Medical University, Taiyuan 030001, P.R. China

1. Liu

Department of Chemical and Materials Engineering, Lyuliang University, Lvliang, 033001, P.R. China

H.F Wang, Q. Gao, Z.B. Yang, Z.M. Wang, C.L. Li, Y.B. Kang, W. Liu, L. Lu, C.W. Zhang

School of Basic Medical Sciences, Shanxi Medical University, Taiyuan 030001, P.R. China

1. mail: liuwen@sxmu.edu.cn (W. Liu); luli@sxmu.edu.cn (L. Lu); [chengwu_zhang@sxmu.edu.cn](mailto:chengwu_zhang@sxmu.edu.cn) (C.W. Zhang)

K. L. Lim

Lee Kong Chian School of Medicine, Nanyang Technological University, Singapore 308232, Singapore

1. **Experimental Section**

**1.1. Materials and instruments**

m-Chloroperoxybenzoic acid (CPBA), NaHCO_3_, Glycerol (Gly), 4',6-diamidino-2-phenylindole (DAPI), Calcein-Acetoxymethyl (Calcein-AM), Propidium Iodide (PI), Deferoxamine (DFO) and Quercetin were purchased from Aladdin Reagent (Shanghai) Co., LTD. Ferrous chloride (FeCl_2_), 3-(4,5-Dimethylthiazol-2-yl)-2,5-diphenyltetrazolium bromide (MTT), Dulbecco's modified eagle medium (DMEM), Fetal bovine serum (FBS), Trypsin-EDTA solution, Paraformaldehyde (PFA) solution, Erastin (ferroptosis activator), Ferrostatin-1 (Fer-1, ferroptosis inhibitor), 2,7-Dichlorodihydrofluorescein diacetate (DCFH-DA), 2,2'-Azino-bis(3-ethylbenzothiazoline-6-sulfonic acid) (ABTS), Fe^2+^ assay kits, LPO assay kits, MDA assay kits, BODIPY™ 581/591 C11 (LPO probe), and Rotenone (Rot) were purchased by Beijing Solarbio Science & Technology Co., Ltd. Tyrosine hydroxylase (TH) antibody, Ionized calcium-binding adapter molecule 1 (Iba-1) antibody, CD206 antibody, α-Syn antibody, Glutathione peroxidase 4 (GPX4) antibody, Fluorescent secondary antibody, and Mitochondrial membrane potential assay kit (JC-10) were purchased from Wuhan Sevier Biotechnology Co., Ltd. ATP Assay Kit, Protein loading marker, Loading buffer, and Goat serum were purchased from Shanghai Biyuntian Biotechnology Co., Ltd. The developing solution was purchased from Guangzhou Yamei Biotechnology Co., Ltd. Poly(lactic-co-glycolic acid)-Poly(ethylene glycol)-Rabies Virus Glycoprotein 29-mer peptide (PLGA-PEG-RVG29) was purchased from Hubei Bo-Green Biotechnology Co., Ltd. Anhydrous ethanol, Anhydrous methanol (MeOH), and Dichloromethane (DCM) were purchased by Tianjin Damao Chemical Reagent Co., Ltd. C57BL/6 mouse were provided by Jetman Inc. Mass spectrum was recorded on a Liquid Chromatography-Mass Spectrometry (Waters UPLC H-Class SQD2, American). ^1^H NMR and ^13^C NMR were taken on a Bruker nuclear magnetic resonance (NMR) spectrometer (AVANCE III 400, German). Fluorescence emission was recorded on fluorescence spectrophotometer (Hitachi F-7100, xenon lamp, Japan). UV-vis absorption spectra was recorded on spectrophotometer (UH 5300 (Japan). The absorbance value was measured by molecular devices microplate reader (Spectra Max190, American). High-performance liquid chromatography (HPLC) analysis for the probe was performed with SHIMADZU LC-20AT systemvwith UV detector, column, Globalsil 5 μm, C18 LP, 4.6 × 250 mm, was performed on a SHIMADZU LC-20AT, and the mobile phase consisted of methanol and water. The particle size distribution and zeta potential were measured using a particle size and zeta potential analyzer (Nano Series, Malvern Panalytical). Cell images were captured by a Olympus confocal laser scanning microscopy (CLSM) (FV3000, Japan). The morphology were captured by scanning electron microscope (SEM) (JSM-7001, Japan). The ultrastructural morphology of the nanoparticles was characterized by Thermo Fisher Scientific High-resolution transmission electron microscope (TEM) (Tecnai G2 20-S-TWIN, American). Small animal fluorescence imaging *in vivo* was performed on Revvity small animal *in vivo* optical imaging system (IVIS Lumina LT, American). The motor balance and coordination mouse were evaluated by a Rotarod Tester (XR-6C, Shanghai).

**1.2. Synthesis of** NP1–5

2-Amino-7-(diethylamino)-3H-benzoxazin-3-one (Pre-NP1), N-(7-(diethylamino)-3-oxo-3H-benzoxazin-2-yl)formamide (Pre-NP2), N-(7-(diethylamino)-3-oxo-3H-benzoxazin-2-yl)propionamide (Pre-NP3), 2-chloro-N-(7-(diethylamino)-3-oxo-3H-benzoxazin-2-yl)acetamide (Pre-NP4), and N-(7-(diethylamino)-3-oxo-3H-benzoxazin-2-yl)acetamide (Pre-NP5) were synthesized by our research group ^[1]^.

**Synthesis of NP1**. Pre-NP1 (0.142 g, 0.50 mmol), CPBA (0.129 g, 0.75 mmol), and NaHCO₃ (0.042 g, 0.50 mmol) were dissolved in DCM, and the reaction was carried out at room temperature for 5 h. After the reaction was completed, the solvent in the reaction solution was removed, and the crude product was purified by silica gel column chromatography (MeOH:DCM = 1:5, v/v) to obtain NP1 as a dark yellow solid (0.042 g, 28.1%). ^1^H NMR (400 MHz, CDCl_3_-*d*) δ 8.20 (s, 1H), 7.78 (d, J=12 Hz, 1H), 7.55 (d, J=8 Hz, 1H), 6.45 (d, J=8 Hz, 2H), 5.21 (s, 2H), 3.70 (t, J=8 Hz, 4H), 1.70 (s, 6H). ^13^C NMR (100 MHz, MeOD-*d*_4_) δ 182.12, 151.09, 150.96, 149.41, 149.36, 143.54, 134.90, 129.15, 119.41, 112.33, 105.29, 99.53, 67.99, 8.64. HR-MS for C_16_H_17_N_3_O_3_ (m/z), Calculated, 300.1348 [M+1]; found, 300.1348.

**Synthesis of NP2**. NP2 was obtained by the condensation of Pre-NP2 (0.156 g, 0.500 mmol) and CPBA (0.129 g, 0.75 mmol) with the same procedure as mentioned above. The crude product was purified by silica gel column chromatography (MeOH:DCM = 1:10, v/v) to obtain NP2 (0.048 g, 29.4 %). ^1^H NMR (400 MHz, MeOD-*d*_4_) δ 8.54 (s, 1H), 8.32 (s, 1H), 8.11 (s, 1H), 7.95 (d, J=8 Hz, 1H), 7.84 (d, J=12 Hz, 1H), 6.44 (s, 1H), 3.95 (m, 2H), 3.73 (m, 2H), 1.14 (t, J=8 Hz, 6H). ^13^C NMR (100 MHz, MeOD-d_4_) δ 181.02, 162.93, 152.16, 151.74, 150.71, 144.70, 134.92, 131.26, 119.80, 115.95, 112.62, 105.69, 68.10, 8.60. HR-MS for C_17_H_18_N_3_O_4_ (m/z), Calculated, 328.1297 [M+1]; found, 328.1300.

**Synthesis of NP3**. NP3 was obtained by the condensation of Pre-NP3 (0.163 g, 0.5 mmol) and CPBA (0.129 g, 0.75 mmol) with the same procedure as mentioned above. The crude product was purified by silica gel column chromatography (MeOH:DCM = 1:10, v/v) to obtain NP3 (0.072 g, 42.3 %). ^1^H NMR (400 MHz, MeOD-*d*_4_) δ 8.27 (s, 1H), 8.09 (s, 1H), 7.92 (d, *J* = 12 Hz, 1H), 7.82 (d, *J* = 8 Hz, 1H), 6.40 (s, 1H), 3.94 (s, 2H), 3.71 (s, 2H), 2.24 (s, 3H), 1.13 (t, *J* = 8 Hz, 6H). ^13^C NMR (101 MHz, MeOD-*d*_4_) δ 181.19, 172.65, 152.01, 151.83, 150.54, 144.62, 139.39, 134.9q1, 131.16, 119.77, 115.02, 112.60, 105.59, 68.10, 24.44, 8.60. HR-MS for C_18_H_19_N_3_O_4_ (m/z), Calculated, 342.1454 [M+1]; found, 342.1455.

**Synthesis of NP4**. NP4 was obtained by the condensation of Pre-NP4 (0.170 g, 0.50 mmol) and CPBA (0.129 g, 0.75 mmol) with the same procedure as mentioned above. The crude product was purified by silica gel column chromatography (MeOH:DCM = 1:10, v/v) to obtain NP4 (0.063 g, 35.6 %). ^1^H NMR (400 MHz, MeOD-*d*_4_) δ 8.27 (s, 1H), 8.08 (s, 1H), 7.91 (s, 1H), 7.81 (s, 1H), 6.40 (s, 1H), 3.93 (s, 2H), 3.71 (s, 2H), 2.54 (s, 2H), 1.17 (s, 3H), 1.12 (s, 6H). ^13^C NMR (100 MHz, MeOD-*d*_4_) δ 181.49, 176.31, 152.18, 152.10, 150.83, 144.85, 139.61, 135.16, 131.36, 119.97, 115.04, 112.82, 105.79, 68.32, 31.45, 9.78, 8.81. HR-MS for C_19_H_21_N_3_O_4_ (m/z), Calculated, 356.1610 [M+1]; found, 356.1617.

**Synthesis of NP5**. NP5 was obtained by the condensation of Pre-NP5 (0.180 g, 0.50 mmol) and CPBA (0.129 g, 0.75 mmol) with the same procedure as mentioned above. The crude product was purified by silica gel column chromatography (MeOH:DCM = 1:20, v/v) to obtain NP5 (0.072 g, 38.2 %). ^1^H NMR (400 MHz, MeOD-*d*_4_) δ 8.33 (s, 1H), 8.13 (d, *J* = 4 Hz, 1H), 7.98 (d, *J* = 8 Hz, 1H), 7.84 (dd, *J* = 4 Hz, 4 Hz, 1H), 6.49 (s, 1H), 4.40 (s, 2H), 3.96 (m, 2H), 3.74 (m, 2H), 1.14 (t, *J* = 4 Hz, 6H). ^13^C NMR (100 MHz, MeOD-*d*_4_) δ 180.70, 167.72, 152.04, 151.40, 150.66, 144.54, 138.51, 134.76, 131.14, 119.65, 115.43 112.44, 105.34, 67.91, 54.59, 43.80, 8.38. HR-MS for C_18_H_18_ClN_3_O_4_ (m/z), Calculated, 376.1074 [M+1]; found, 376.1066.

**1.2.** **Synthesis Route**

**Scheme S1.** Synthesis route of NP1-5.

**1.3.** **Structural Characterizations of NP1-5**


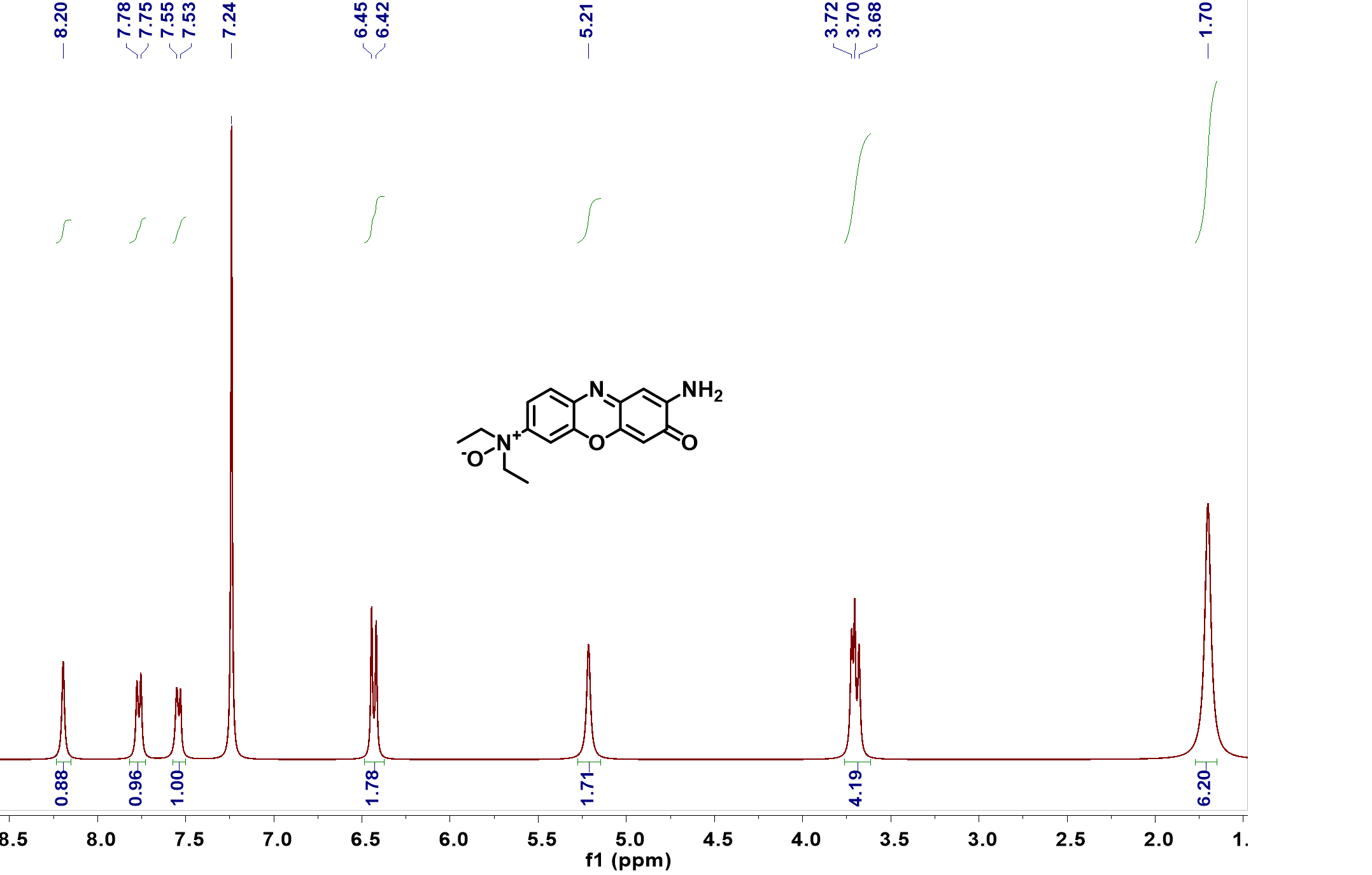


***Figure S1****.* ^1^H NMR of NP-1.


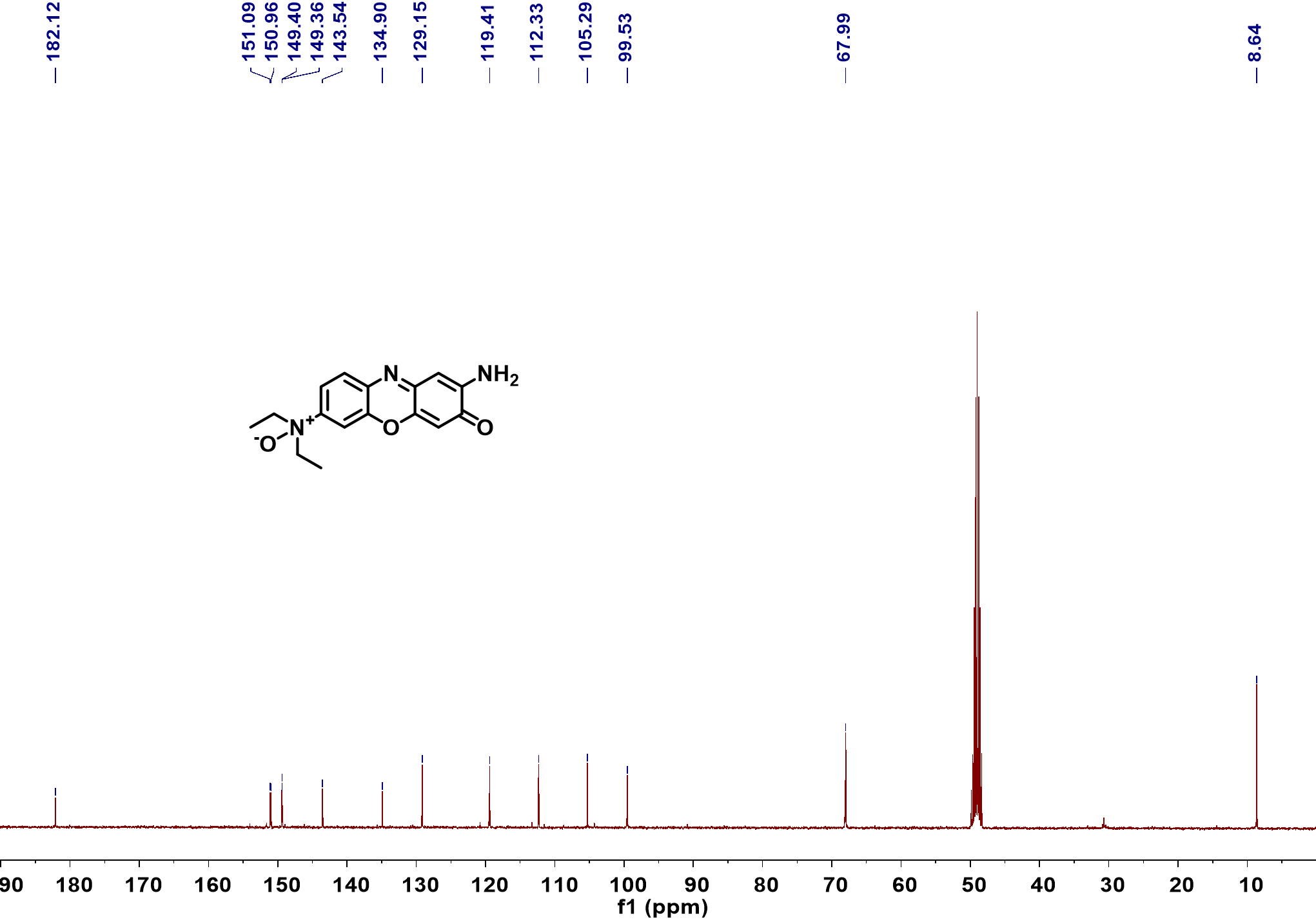


***Figure S2.*** ^13^C NMR of NP-1.


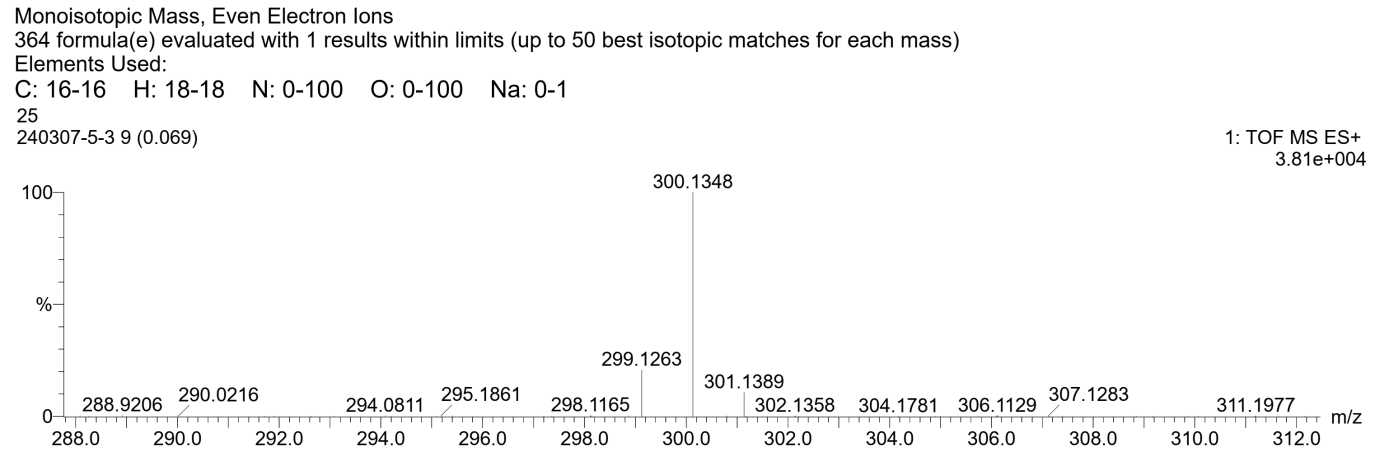


***Figure S3.*** HRMS of NP-1.


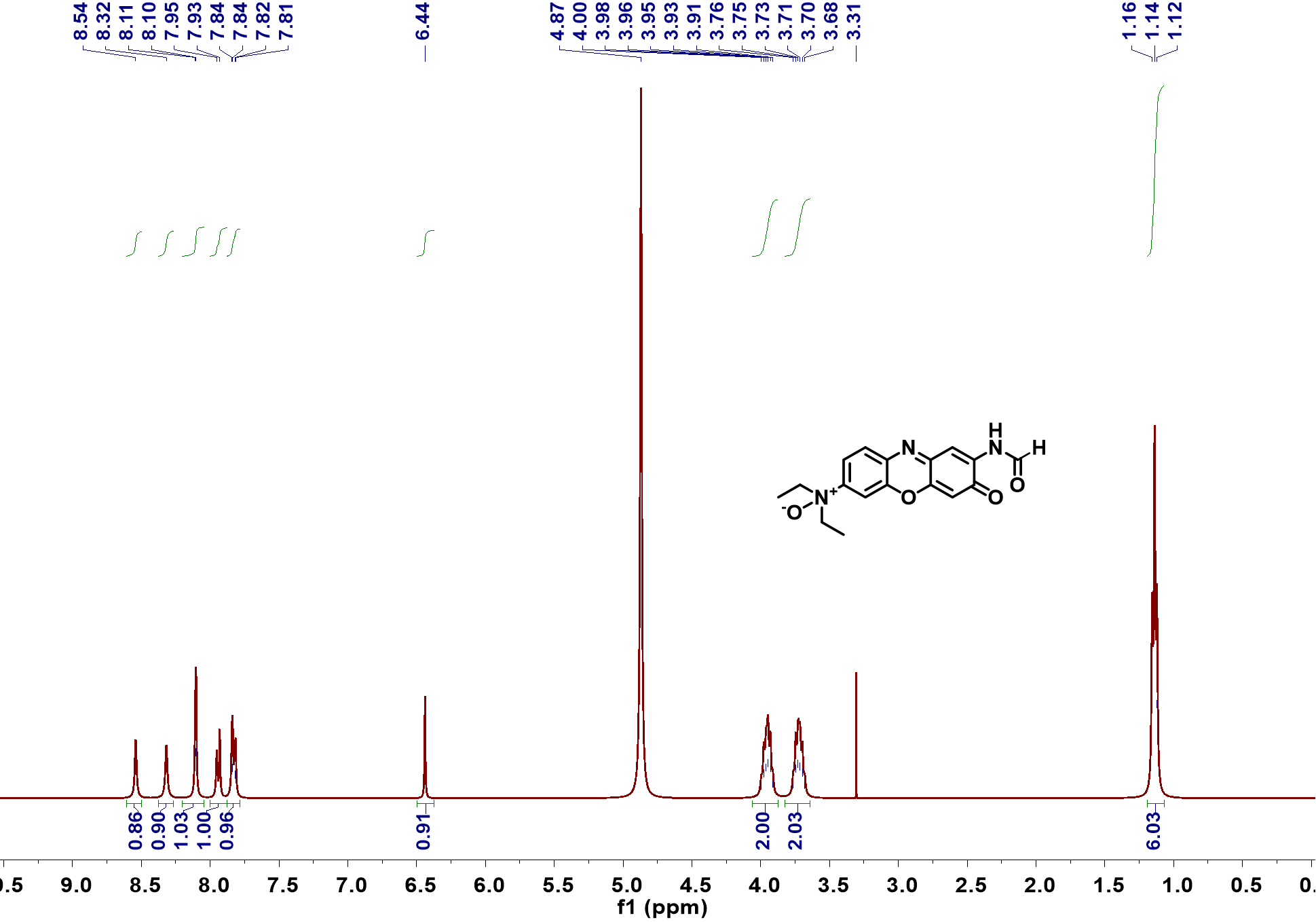


***Figure S4****.* ^1^H NMR of NP-2.


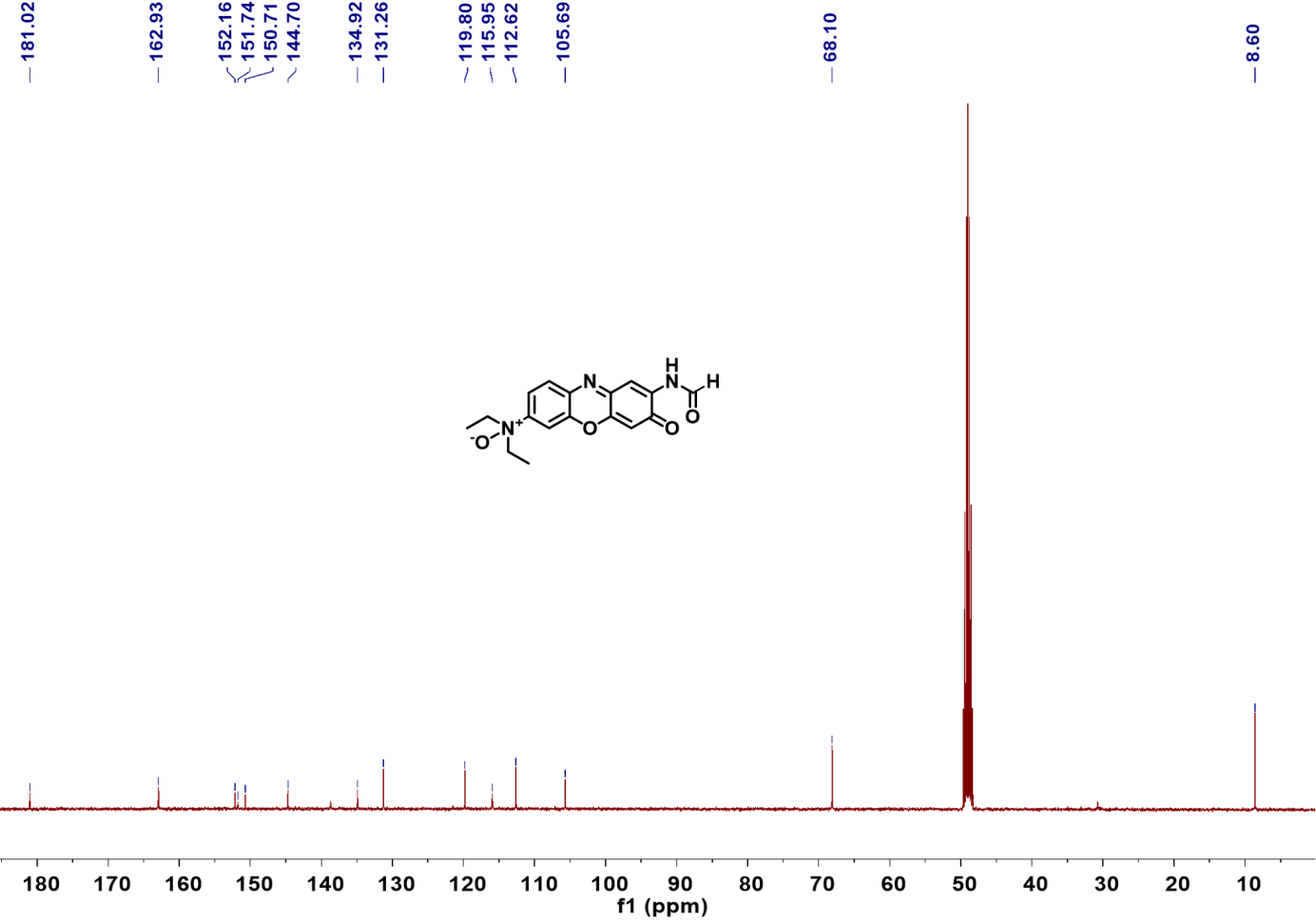


***Figure S5.*** ^13^C NMR of NP-2.


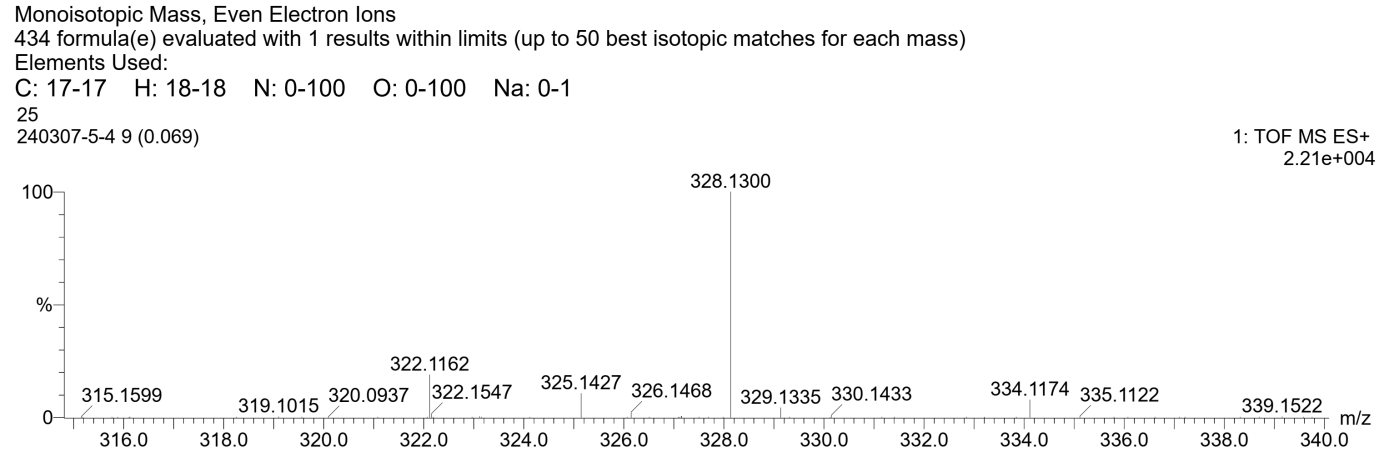


***Figure S6.*** HRMS of NP-2.


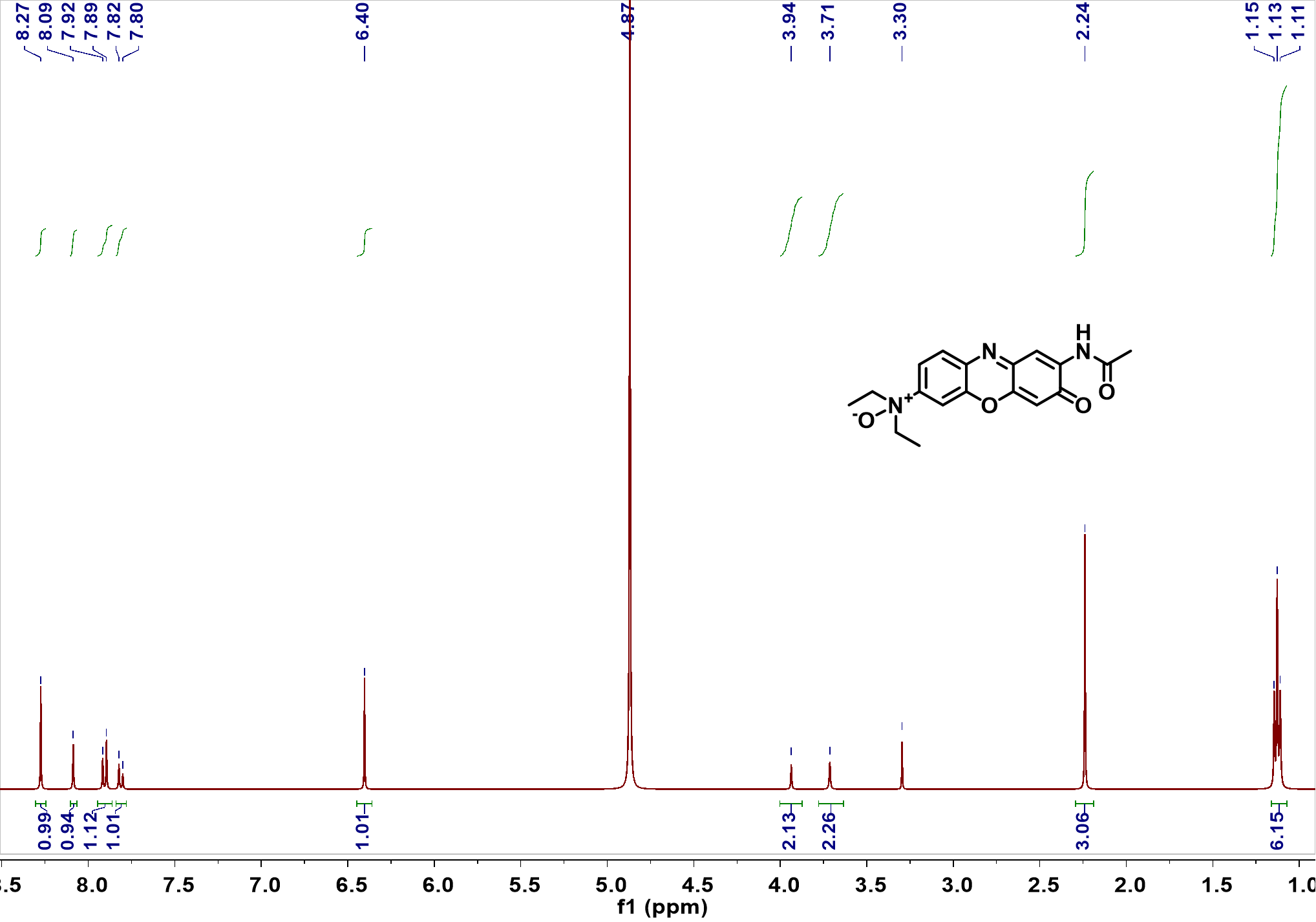


***Figure S7****.* ^1^H NMR of NP-3.


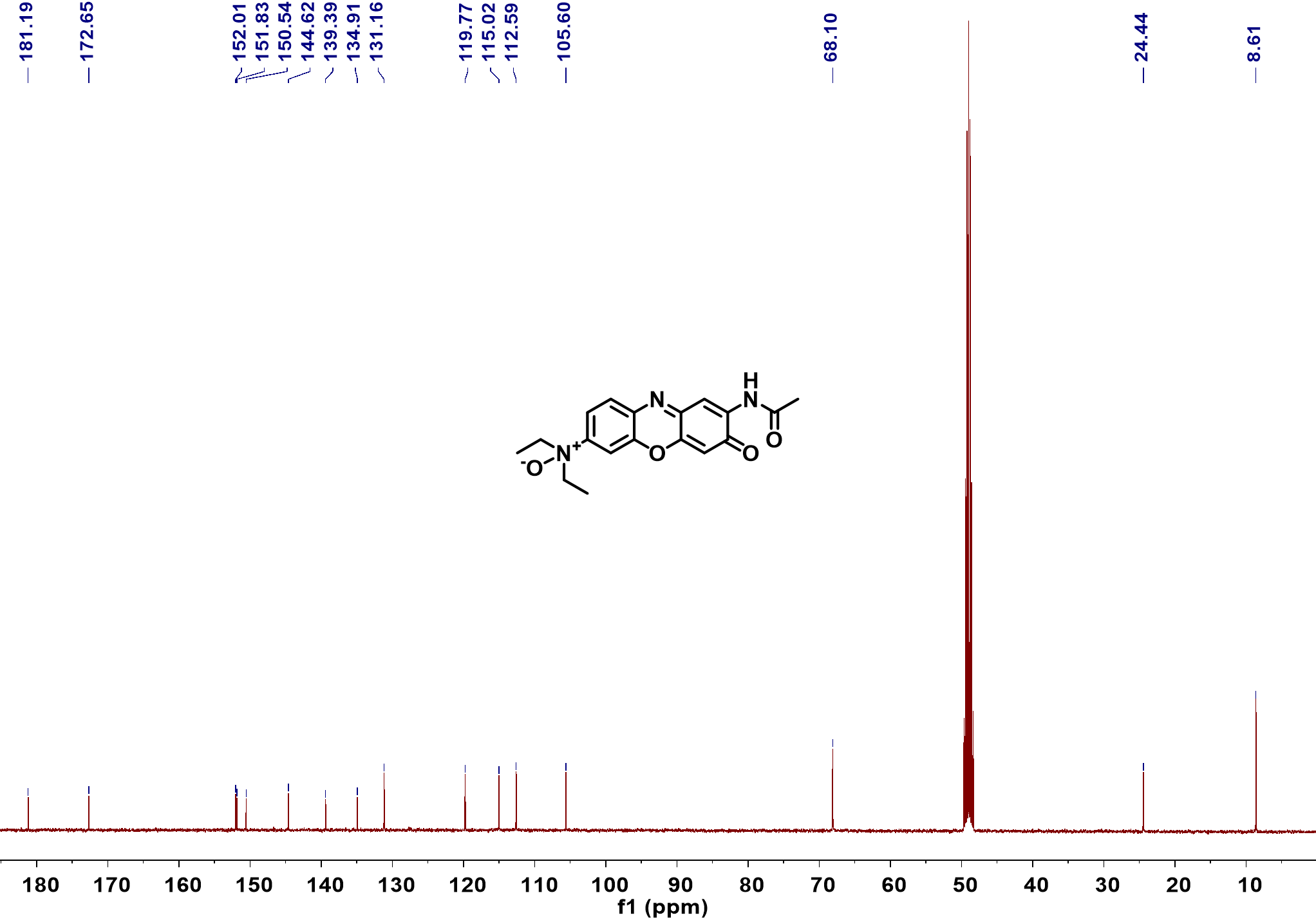


***Figure S8.*** ^13^C NMR of NP-3.


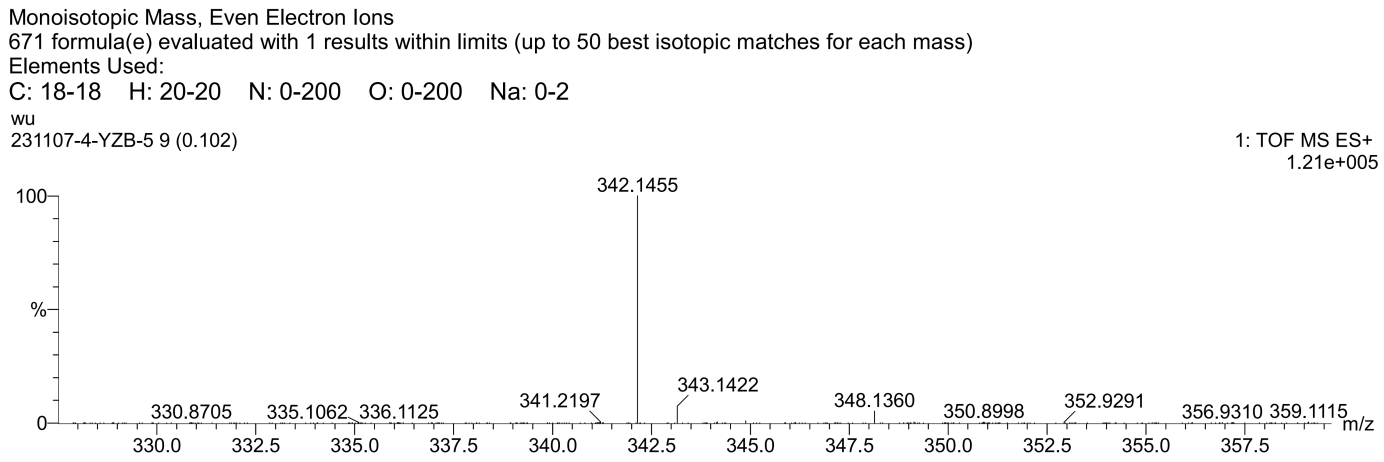


***Figure S9.*** HRMS of NP-3.


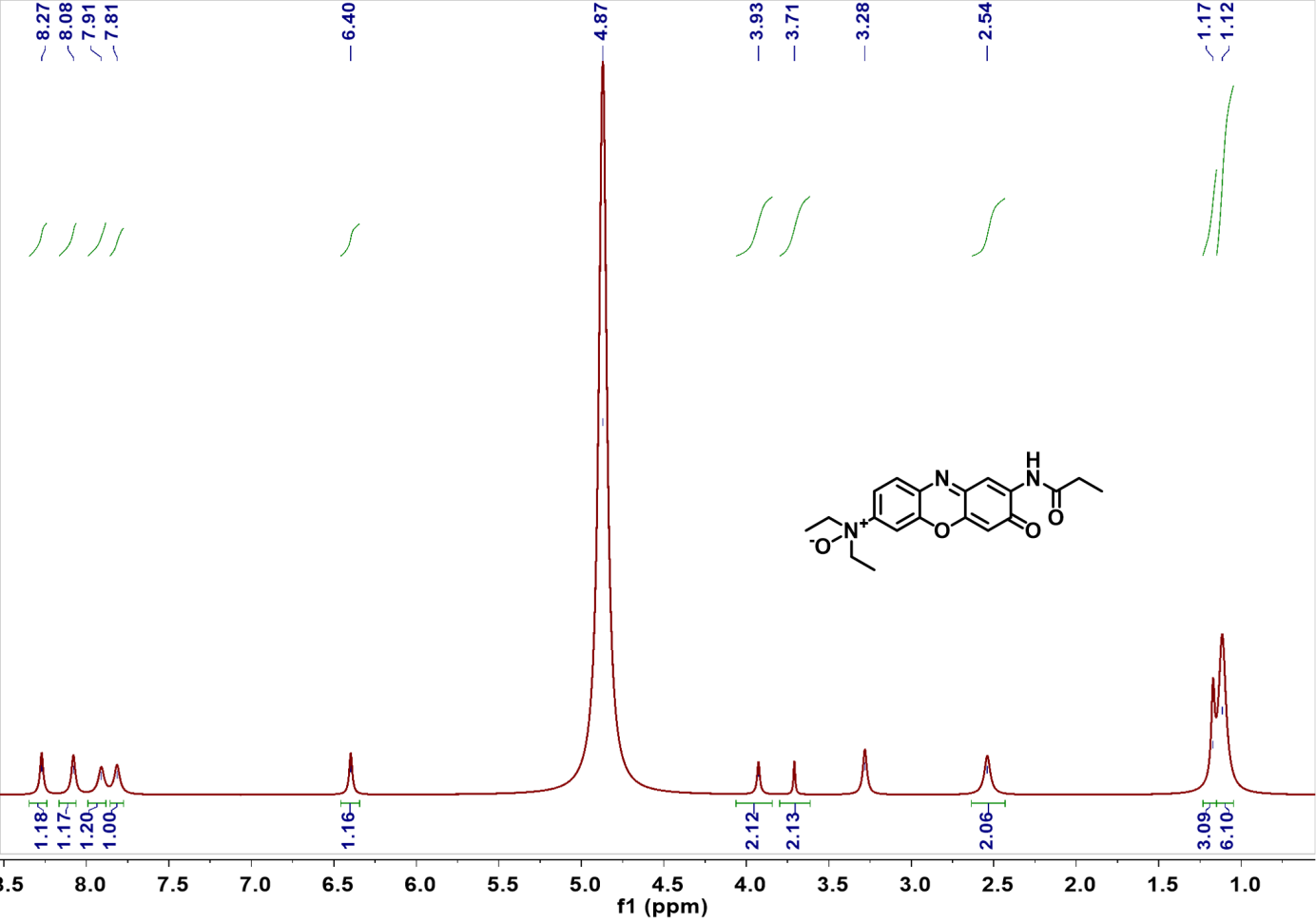


***Figure S10****.* ^1^H NMR of NP-4.


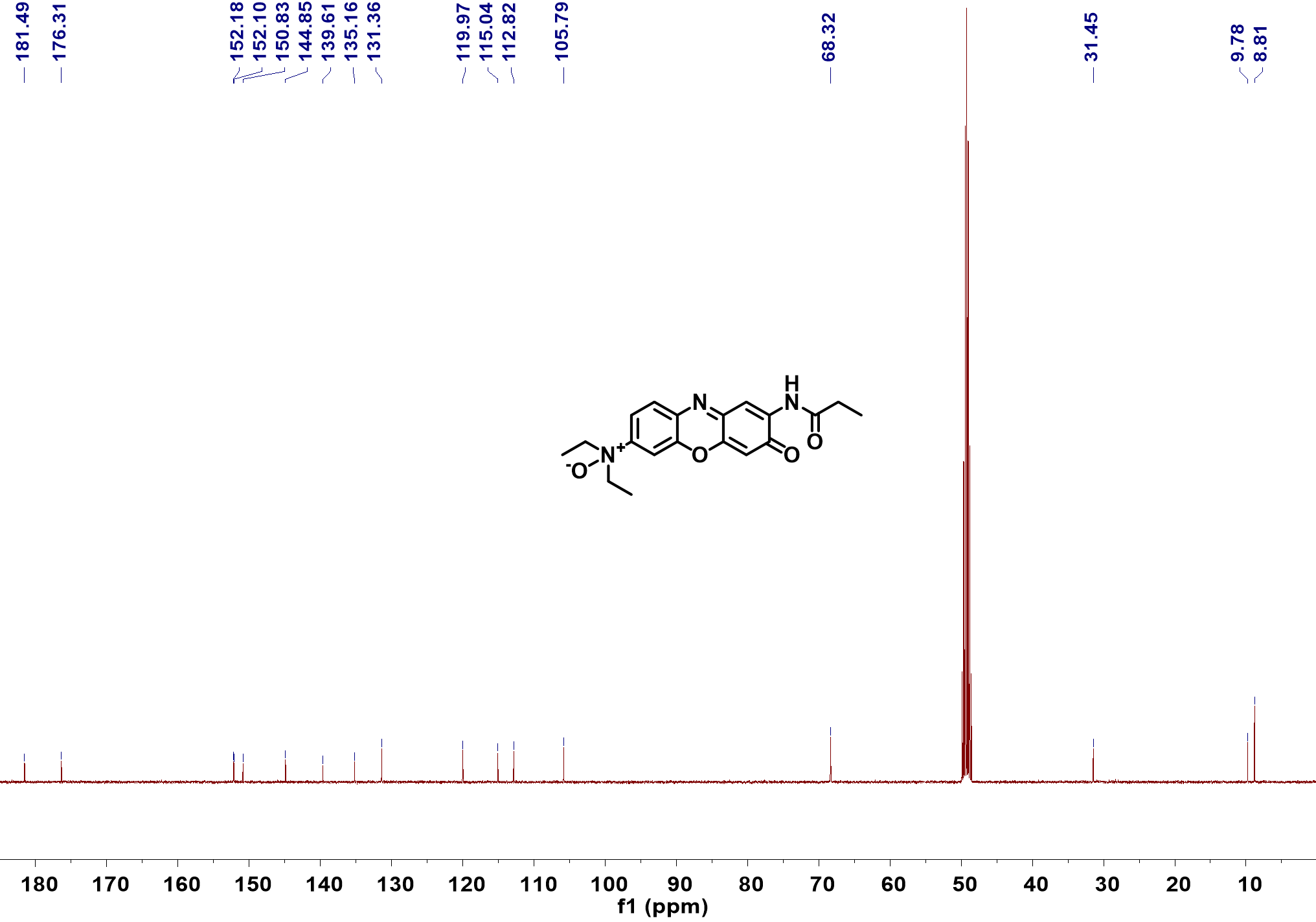


***Figure S11.*** ^13^C NMR of NP-4.


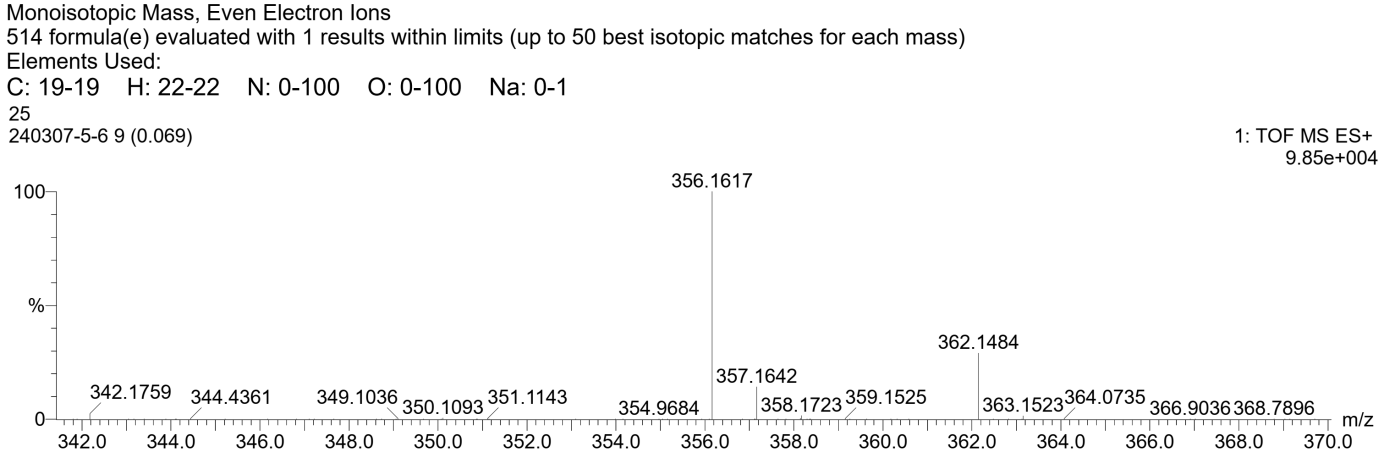


***Figure S12.*** HRMS of NP-4.


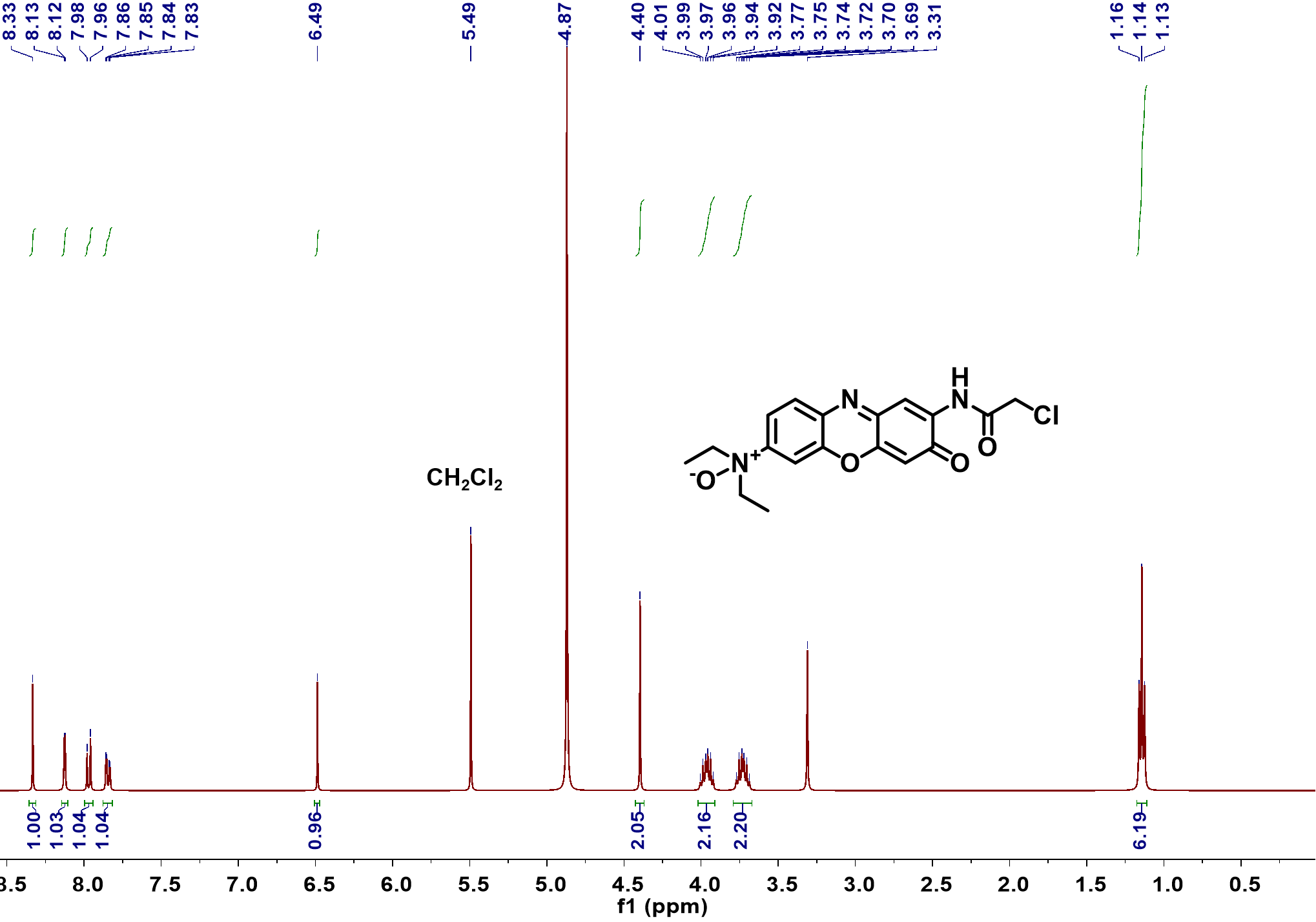


***Figure S13****.* ^1^H NMR of NP-5.


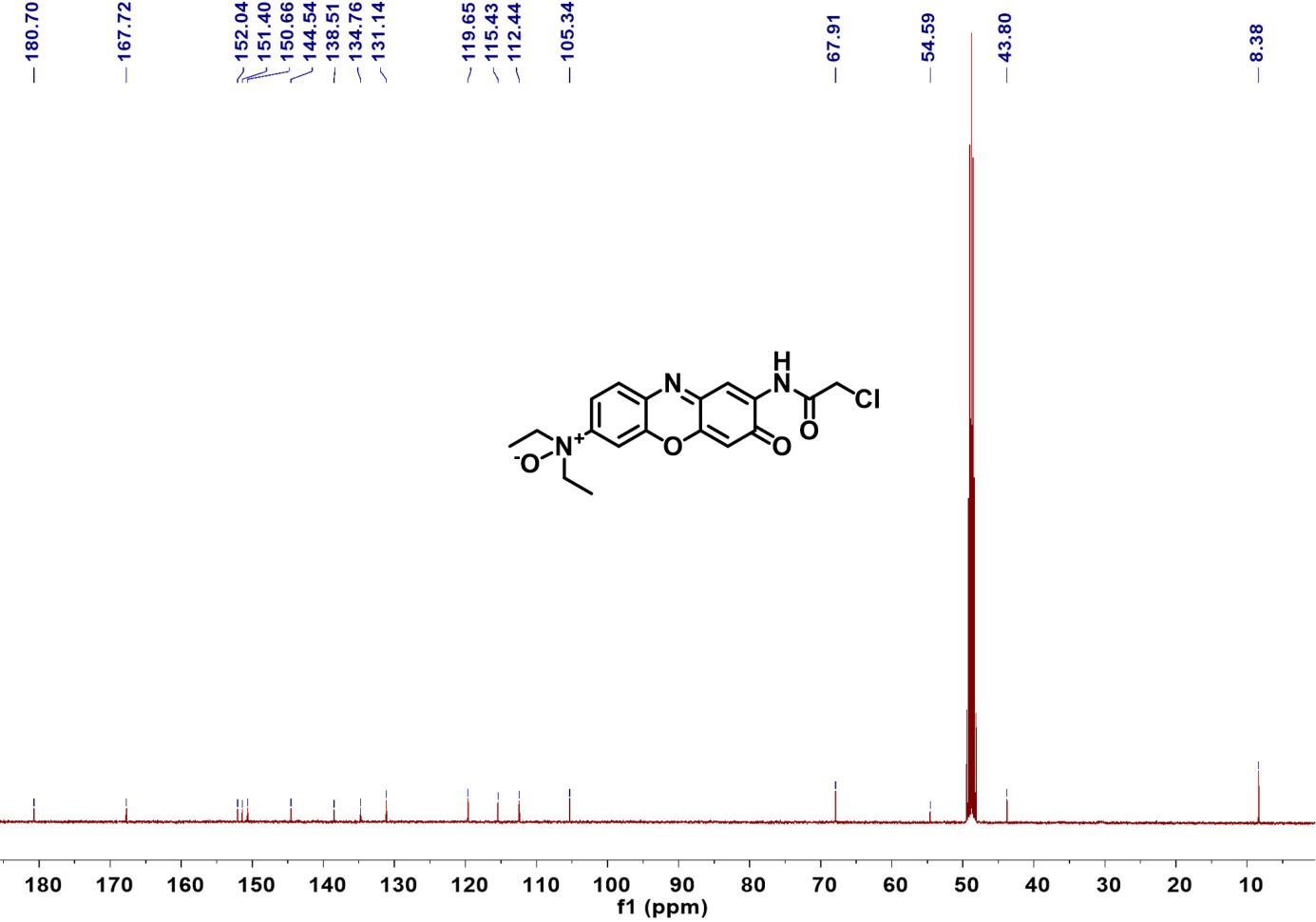


***Figure S14.*** ^13^C NMR of NP-5.


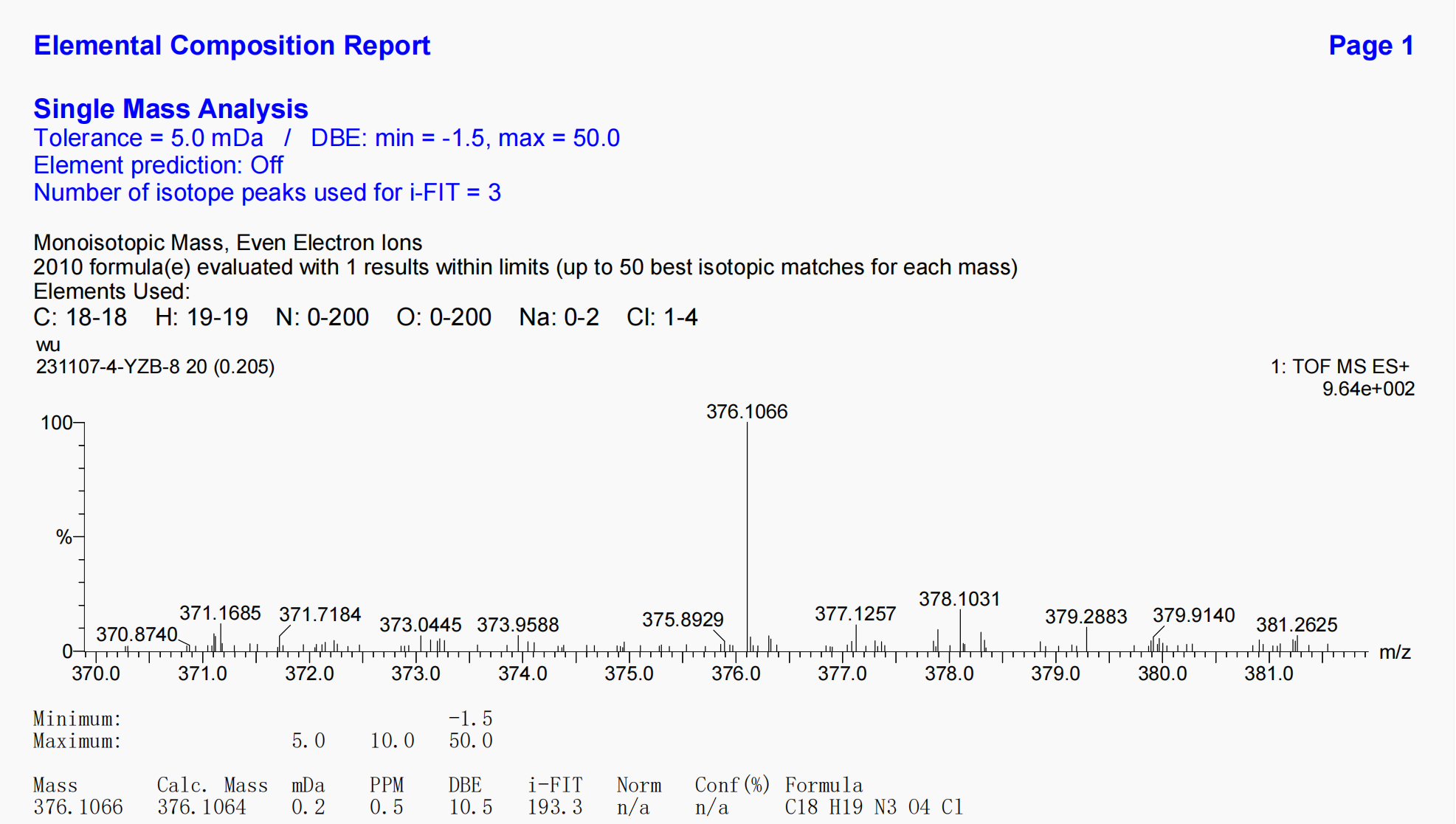


***Figure S15.*** HRMS of NP-5.

1. **Supporting figures**

***Figure S16.*** Time-dependence of probe NP3 (10 μM) in response to Fe²⁺(100 μM), Gly (10%), and Fe²⁺/Gly. The error bars represent the standard deviation (n=3).

***Figure S17.*** Fluorescence intensity of probe NP3 (10 μM) at different pH in the presence or absence of the Fe²⁺/Gly ( Fe²⁺ was 100 μM, Gly was 10%). The error bars represent the standard deviation (n=3).

***Figure S18.*** Fluorescence intensity of probe NP3 (10 μM) at different temperatures in the presence or absence of the Fe²⁺/Gly ( Fe²⁺ was 100 μM, Gly was 10%). The error bars represent the standard deviation (n=3).

***Figure S19.*** Cell viability of PC-12 cell treated with different concentrations of NP3. The error bars represent the standard deviation (n=6).


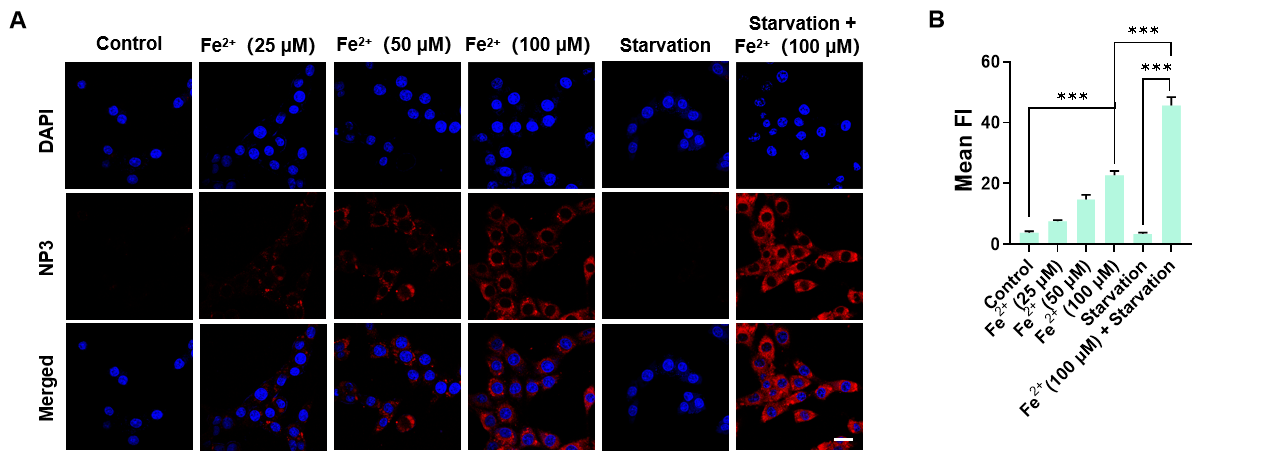


***Figure S20.*** (A) Confocal imaging and (B) Quantitative statistical graph of PC-12 cells with different treatment (Scale bar: 20 µm). PC-12 cells supplemented with different concertration of Fe^2+^ or starvation at 37 ^o^C for 30 min to construct high Fe^2+^ cellular model and high-viscosity cellular model. Data presented as mean ± SD, n=3 for each, *Student’s t-test*, ***p < 0.001.


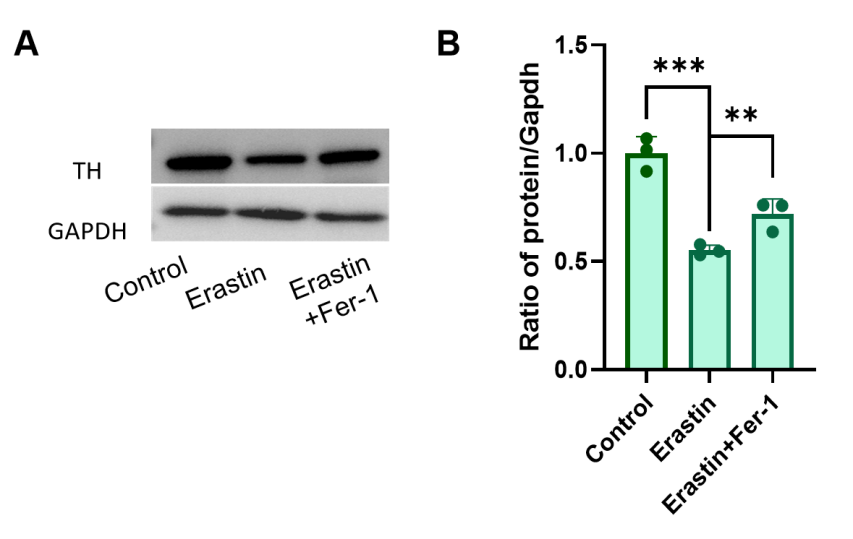


***Figure S21.*** (A) WB showing the effects of Erastin and Fer-1 on TH expression in PC-12 cells. (B) Quantitative statistical graph of WB of TH expression with different treatment. Data presented as mean ± SD, n=3 for each, *ANOVA test*, **p < 0.01, and ***p < 0.001.


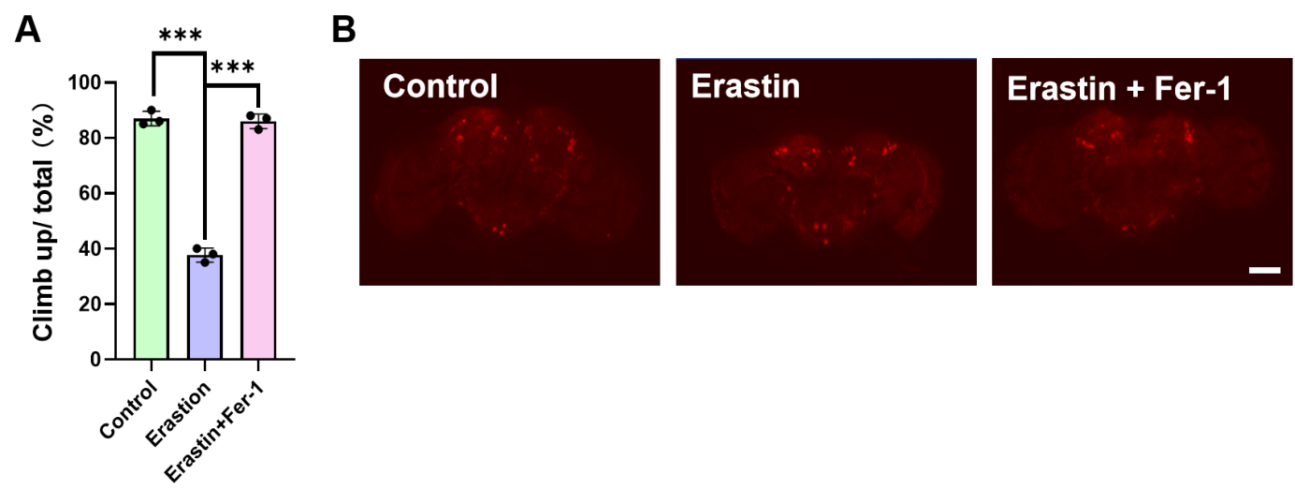


***Figure S22.*** (A) Statistical graph of climbing behavior in Drosophila. (B) Fluorescence imaging of DA neurons in drosophila brain (Scale bar: 200 μm). Data presented as mean ± SD, n=3 for each, *ANOVA test*, ***p < 0.001.


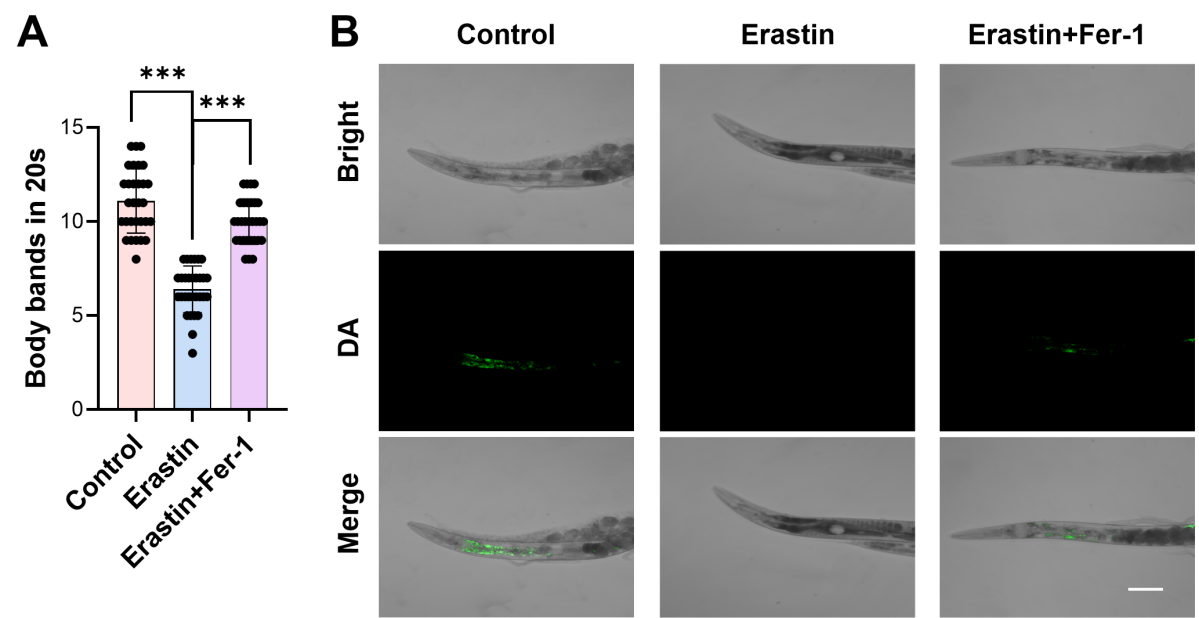


***Figure S23.*** (A) Quantitative statistical graph of *C. elegans* behavior. (B) Fluorescence imaging of DA neurons in *C. elegans* (Scale bar: 200 μm). Data presented as mean ± SD, n=30 for each, *ANOVA test*, ***p < 0.001.

***Figure S24.*** Fluorescence spectra of PQR NPs (50 μg/mL) in response to Gly of different volume percentages.

***Figure S25.*** Fluorescence spectra of PQR NPs (50 μg/mL) in response to Fe²⁺ of different concentrations (0-150 μM).


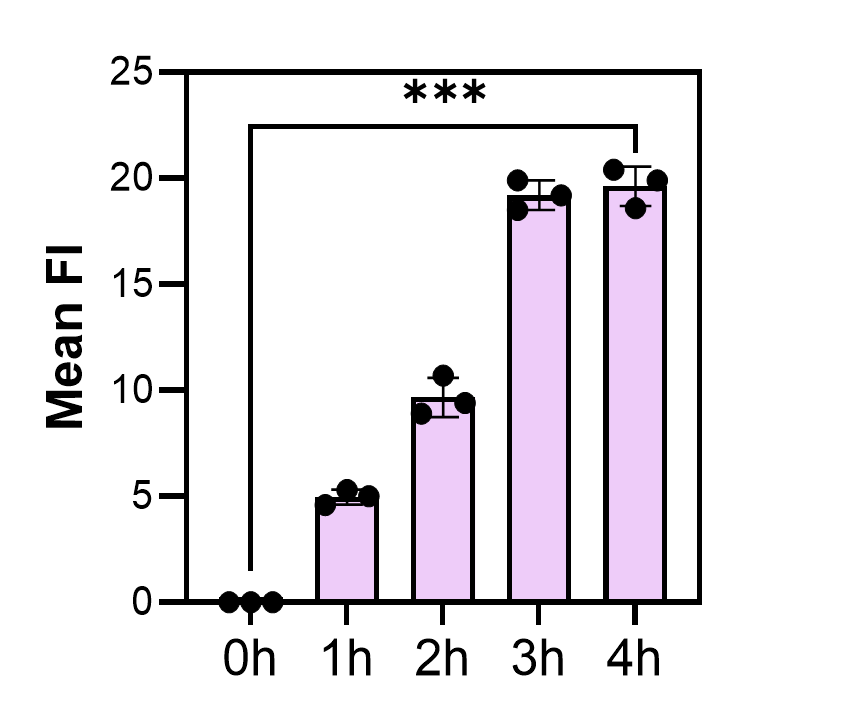


***Figure S26.*** Quantitative statistical graph of cellular fluorescence images of PC-12 cell with PQR NPs (50 μg/mL) at different time points. Data presented as mean ± SD, n=3 for each, *Student’s t-test*, ***p < 0.001.

***Figure S27.*** Quantitative statistical graph of fluorescence images of PC-12 cells with PQR NPs (50 μg/mL) after treated by Erastin, Erastin+Fer-1. Data presented as mean ± SD, n=3 for each, *ANOVA test*, ***p < 0.001.

***Figure S28.*** Viability of PC-12 cells with different concentration Rot. Data presented as mean ± SD, n=3 for each, *Student’s t-test*, **p < 0.01 and ***p < 0.001.


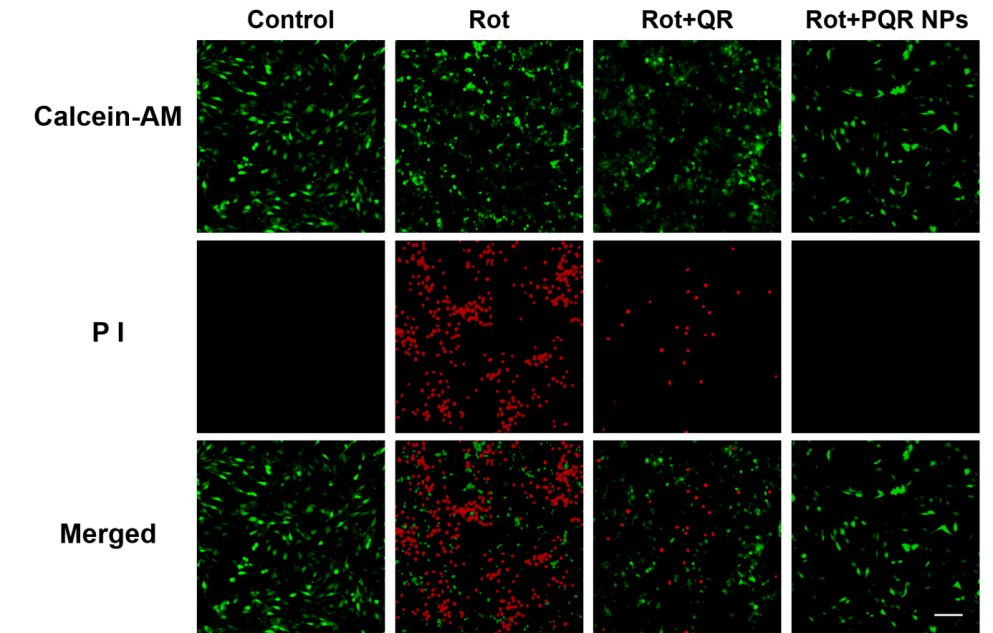


***Figure S29.*** Calcein-AM/PI double-staining PC-12 cells in different treatment groups (Scale bar: 100 μm).


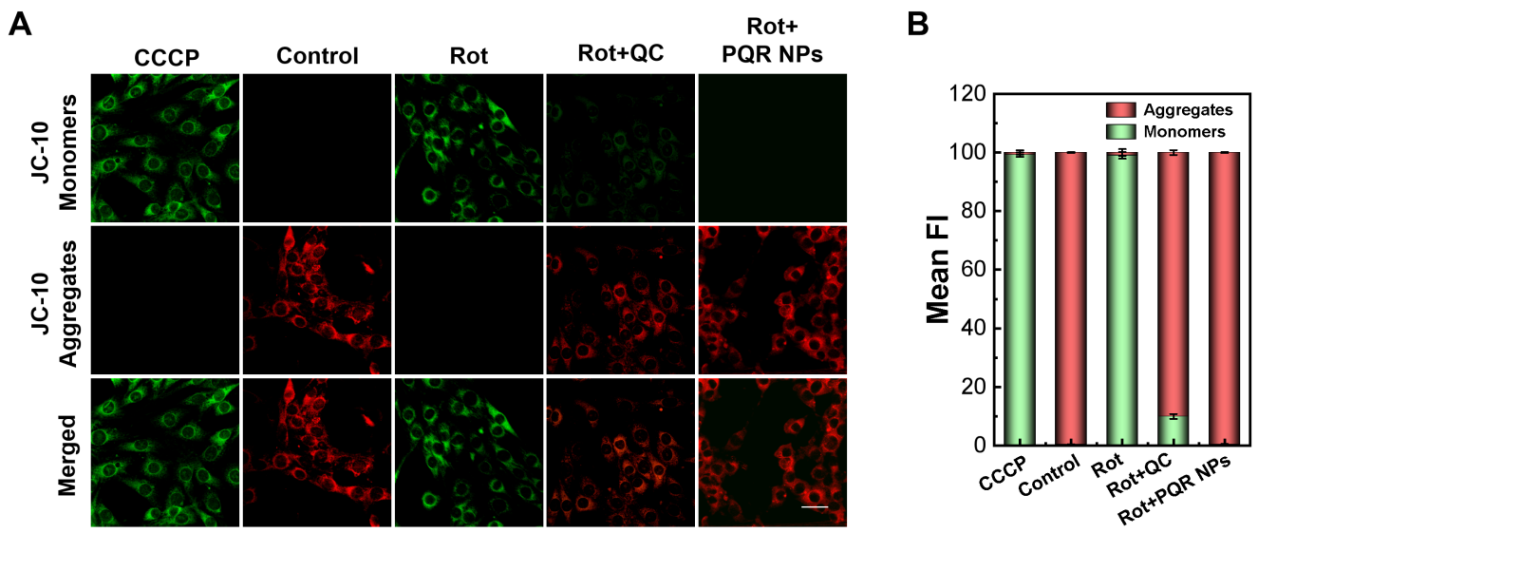


***Figure S30.*** (A) Fluorescence imaging and (F) Quantitative statistical graph of MMP in PC-12 cells of different treatments labeled with JC-10 probe (Scale bar: 20 μm). The error bars represent the standard deviation (n=3).

***Figure S31.*** ATP levels in PC-12 cells with different treatments. Data presented as mean ± SD, n=3 for each, *Student’s t-test*, ***p < 0.001.


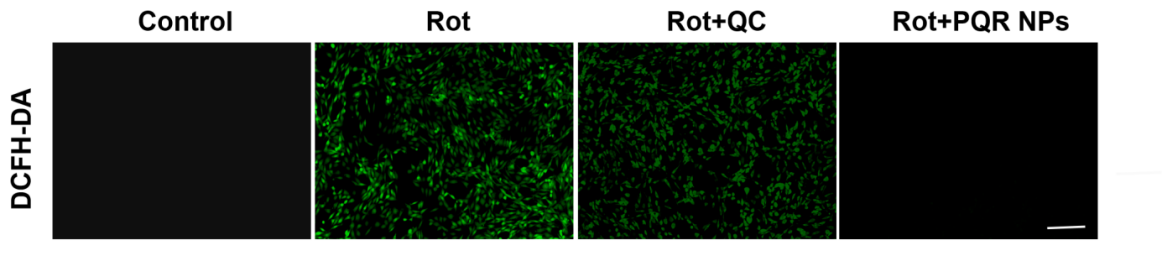


***Figure S32*.** Fluorescence imaging of ROS in PC-12 cells of different treatment groups labeled with DCFH-DA probe (Scale bar: 100 μm).

***Figure S33.*** Quantitative statistical graph of fluorescence imaging of PC-12 cells in the lower chamber of Transwell. Data presented as mean ± SD, n=6 for each, *ANOVA test,* ***p < 0.001.


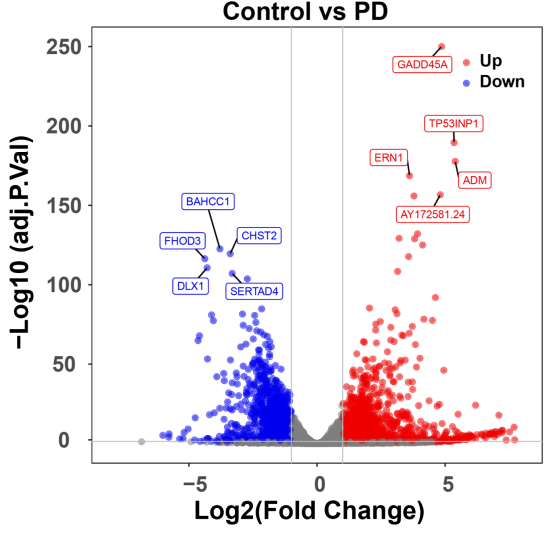


***Figure S34.*** Volcano plot showing the DEGs in Control and Erastin induced PC-12 cells.


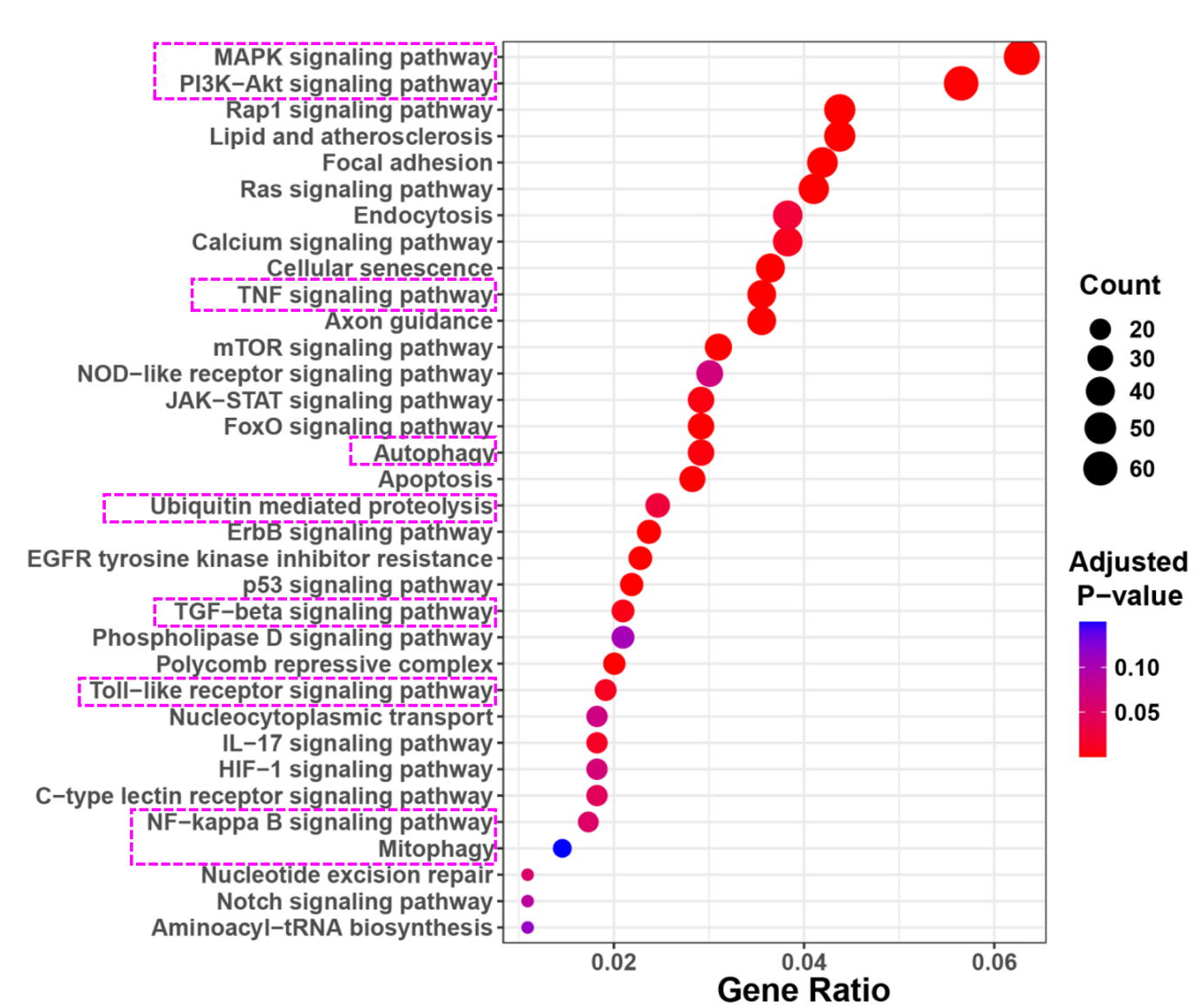


***Figure S35.*** KEGG enrichment analysis of DEGs between the control and PD group.


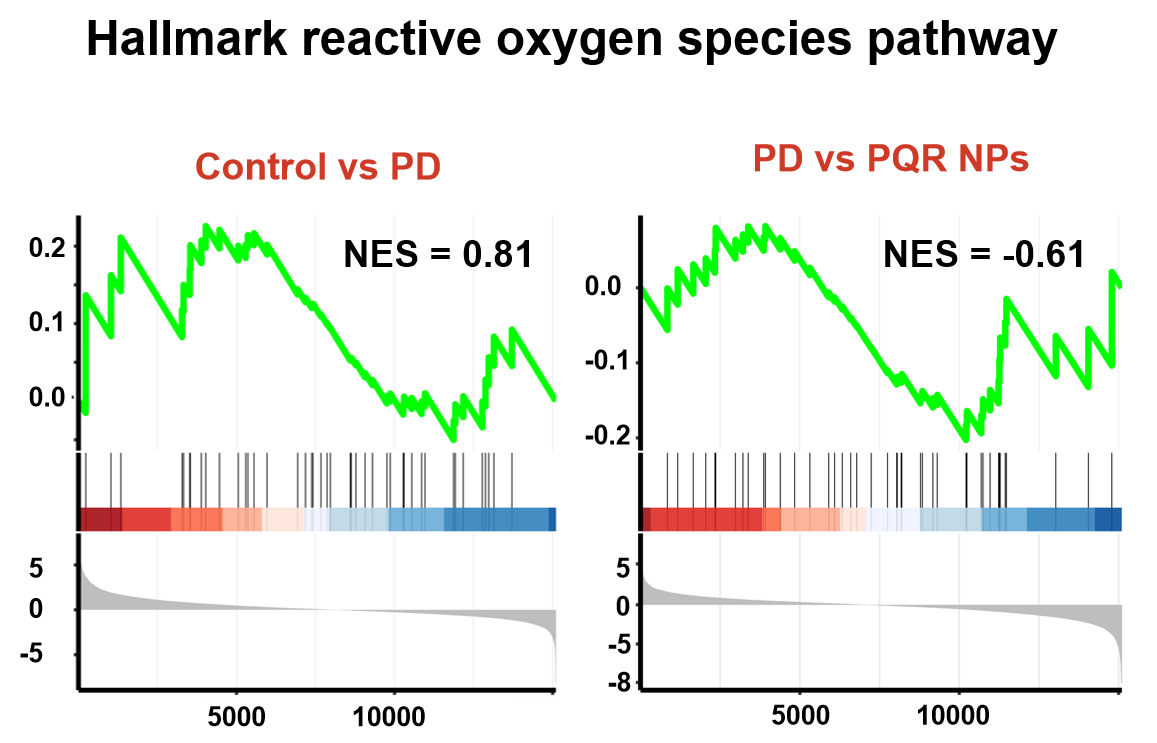


***Figure S36.*** GSEA analysis presenting the expression trend changes of reactive oxygen species pathway in the different groups.

***Figure S37*.** Quantitative statistical graph of WB of TH expression with different treatment. Data presented as mean ± SD, n=3 for each, *ANOVA test*, *p < 0.05, **p < 0.01, and ***p < 0.001.

***Figure S38.*** Quantitative statistical graph of WB of GPX4 expression with different treatment. Data presented as mean ± SD, n=3 for each, *ANOVA test*, *p < 0.05, **p < 0.01.

***Figure S39.*** Quantitative statistical graph of α-Syn IFL staining in different groups. Data presented as mean ± SD, n=6 for each, *ANOVA test*, ***p < 0.001.


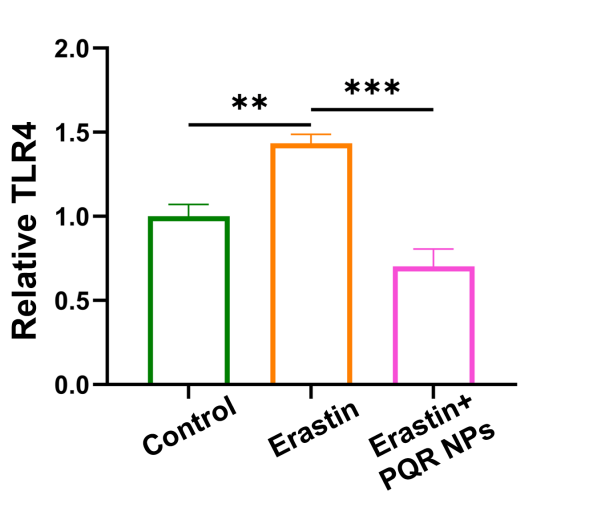


***Figure S40*.** qPCR validation of TLR4 mRNA levels in PC-12 cells. Data presented as mean ± SD, n=3 for each, *ANOVA test*, **p < 0.01, and ***p < 0.001.


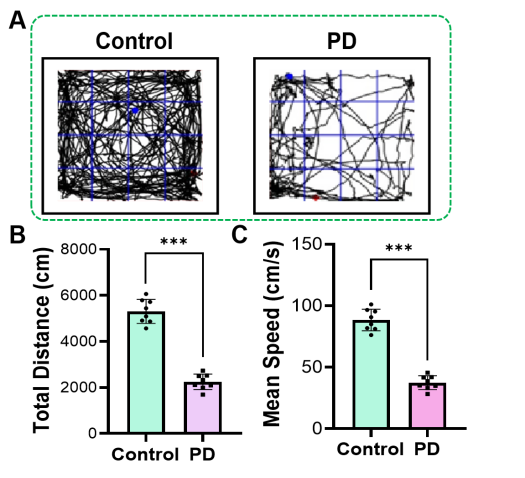


***Figure S41.*** (A) Movement trajectory diagrams, (B) Total distance and (C) Mean speed of control and MPTP induced PD mice in open-field test. Data presented as mean ± SD, n=8 for each, *Student’s t-test*, ***p < 0.001.


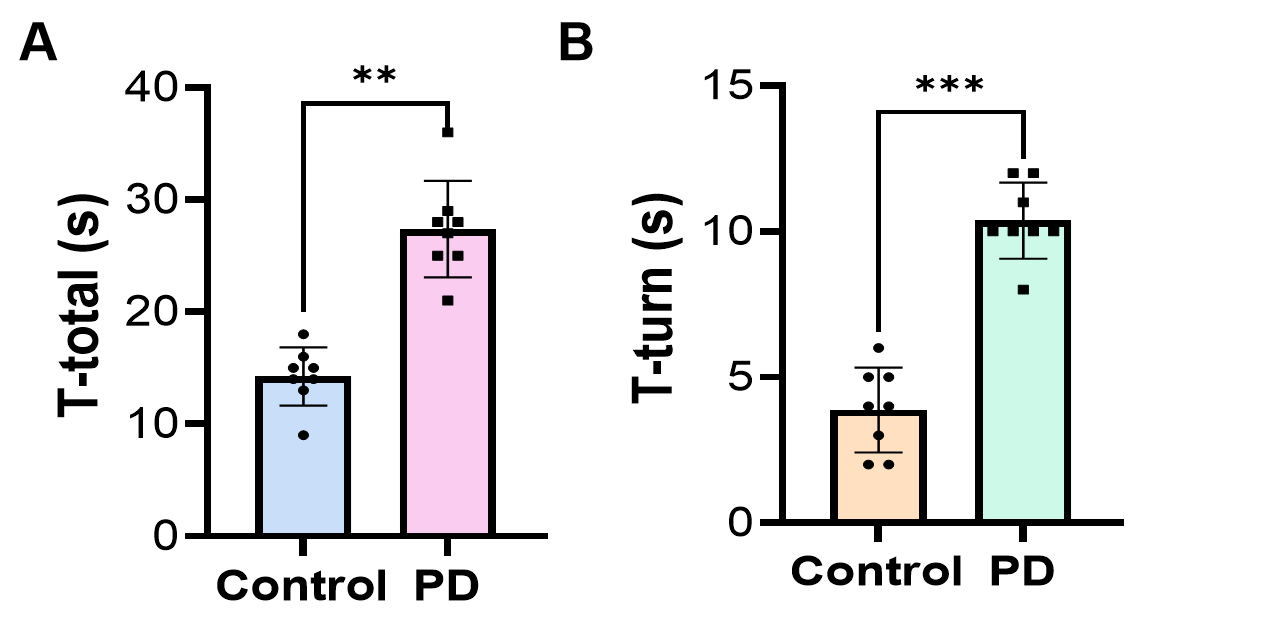


***Figure S42*.** (A) Total time (T-total) and (B) Turning time (T-Turn) in the pole test of Control and MPTP induced PD mice. Data presented as mean ± SD, n=8 for each, *Student’s t-test*, **p < 0.01, and ***p < 0.001.

***Figure S43*.** Latency of drop off in the rotarod test of control and MPTP induced PD mice. Data presented as mean ± SD, n=8 for each, *Student’s t-test*, ***p < 0.001.


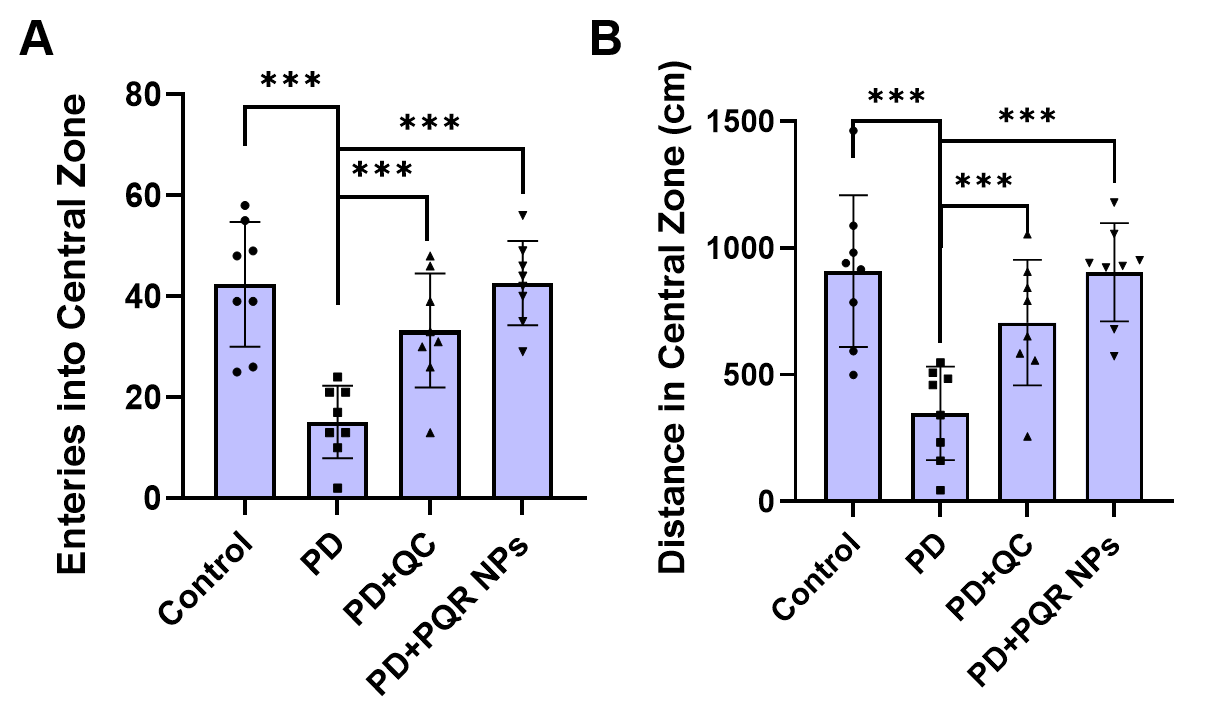


***Figure S44*.** Open field test of mice with different treatments. (A) Number of entries into the central zone, (B) Distance traveled in the central zone. Data presented as mean ± SD, n=8 for each, *ANOVA test*, ***p < 0.001.


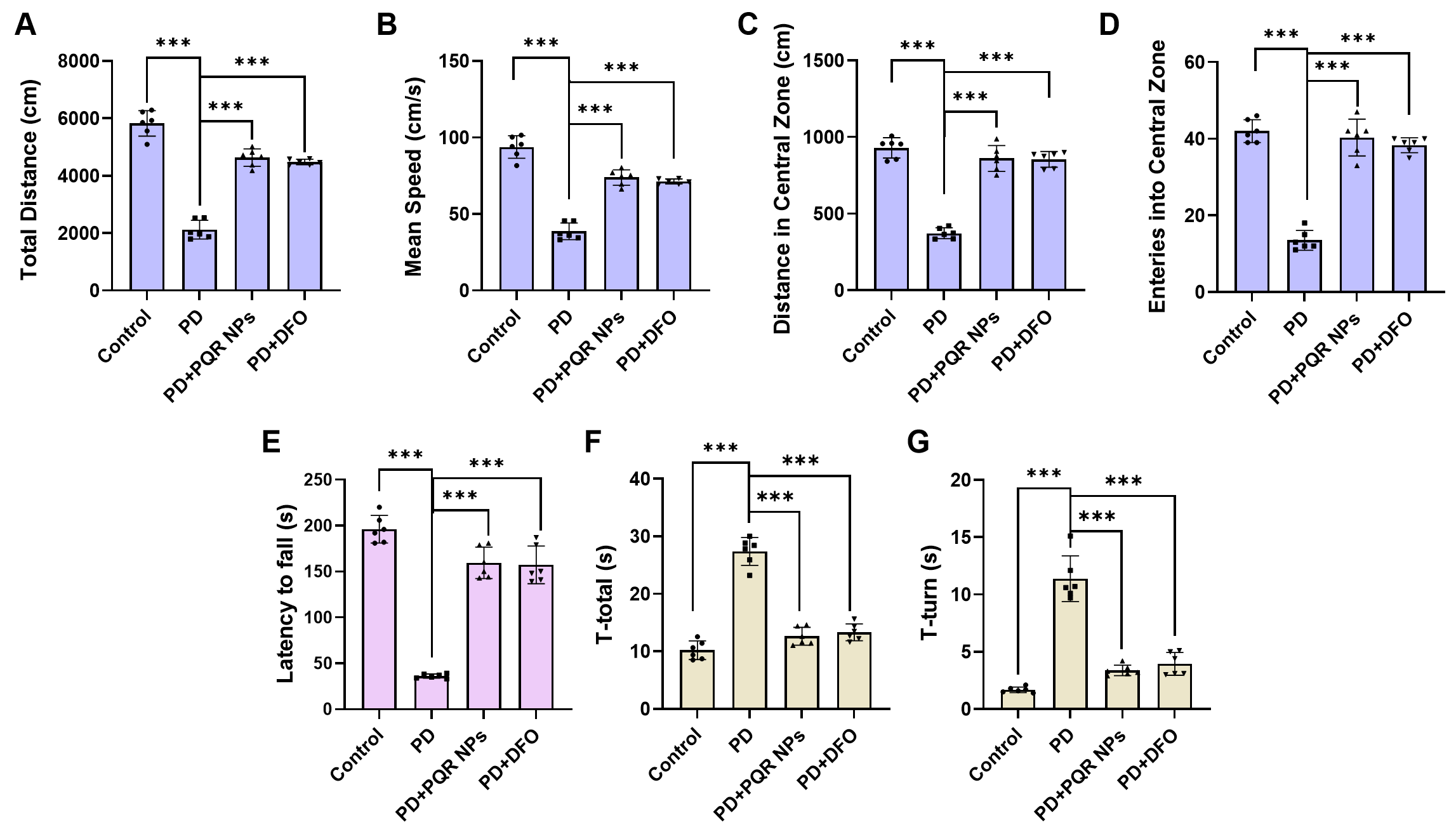


**Figure *S45***. Behavioral test of mice with different treatments. (A) Total distance, (B) Mean speed, (C) Distance traveled in the central zone, (D) Number of entries into the central zone, (E) Latency of drop off in the rotarod test, (F) T-total and (G) T-Turn in the pole test of mice in different groups. Data presented as mean ± SD, n=6 for each, *ANOVA test*, ***p < 0.001.


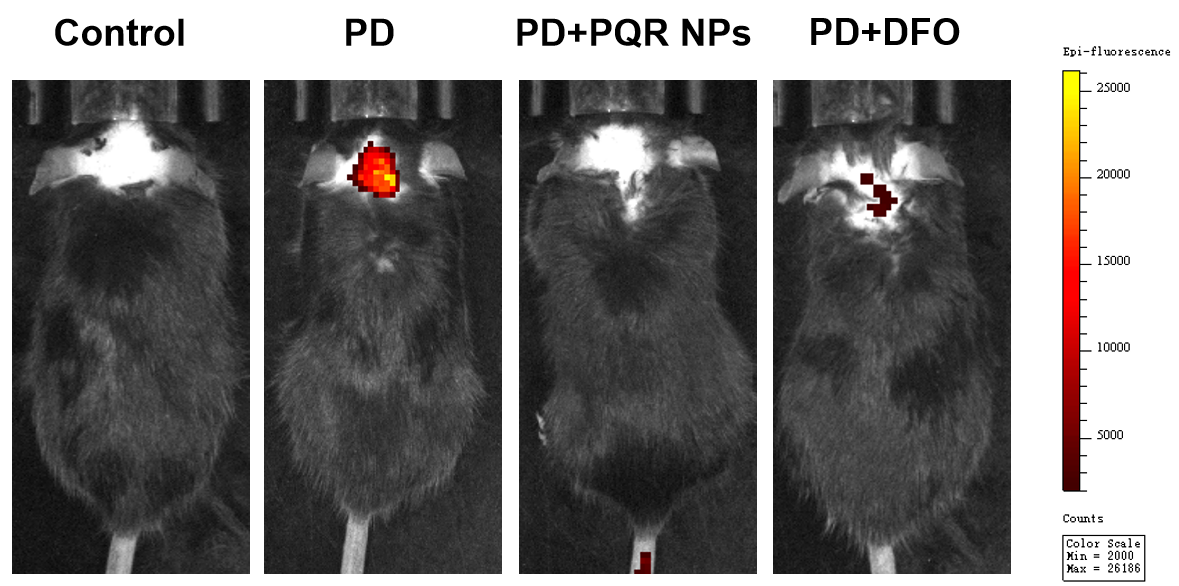


***Figure S46*.** PQR NPs monitoring the intervention effect on MPTP induced PD mice.


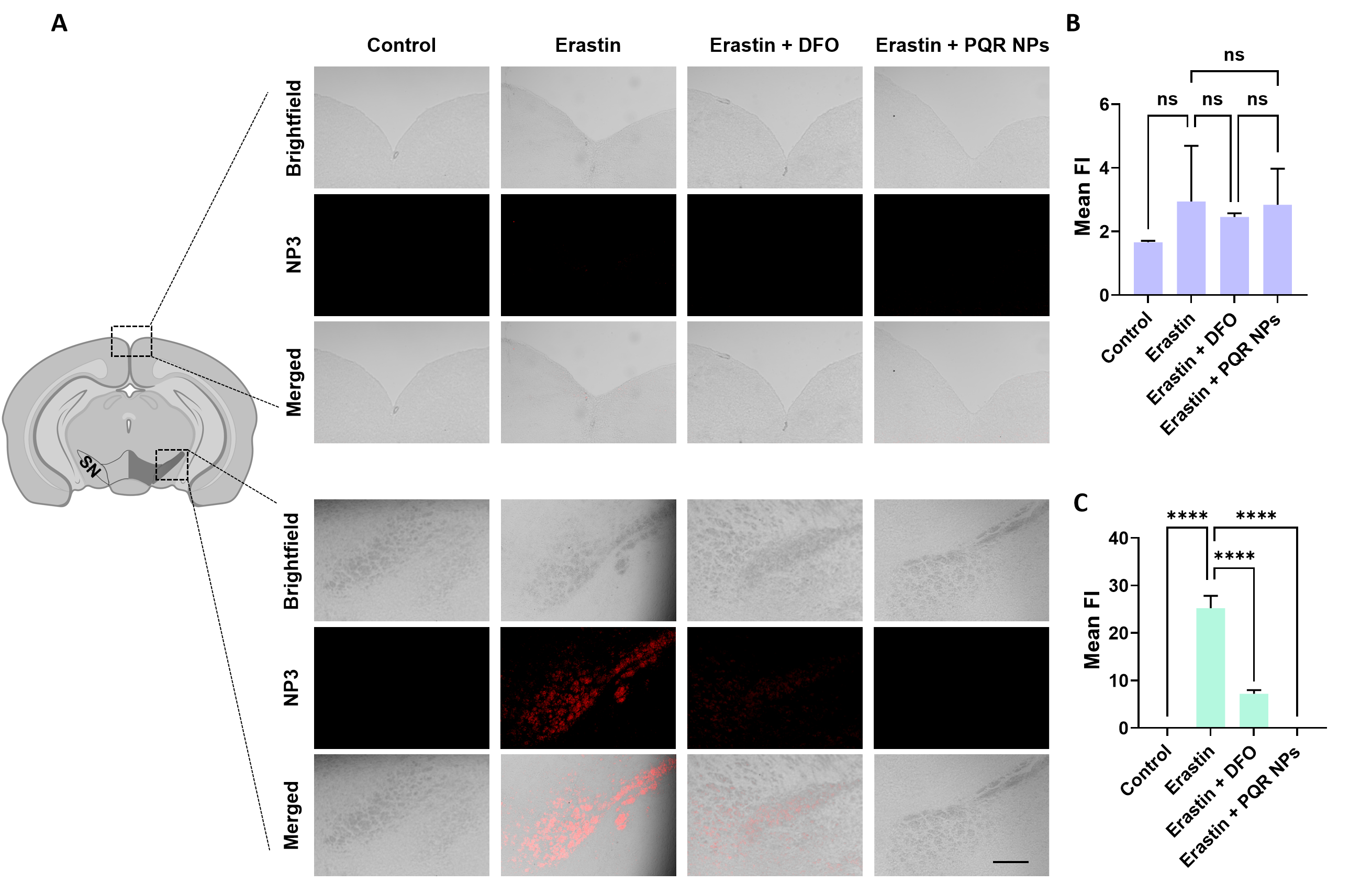


***Figure S47*.** (A) Confocal imaging of NP3 in cerebral cortex and substantia nigra, and Quantitative statistical analysis of (B) the cerebral cortex and (C) substantia nigra in mice brain with different treatments. Data presented as mean ± SD, n=3 for each, *ANOVA test*, ^ns^P > 0.05, *p < 0.05, **p < 0.01, and ***p < 0.001.


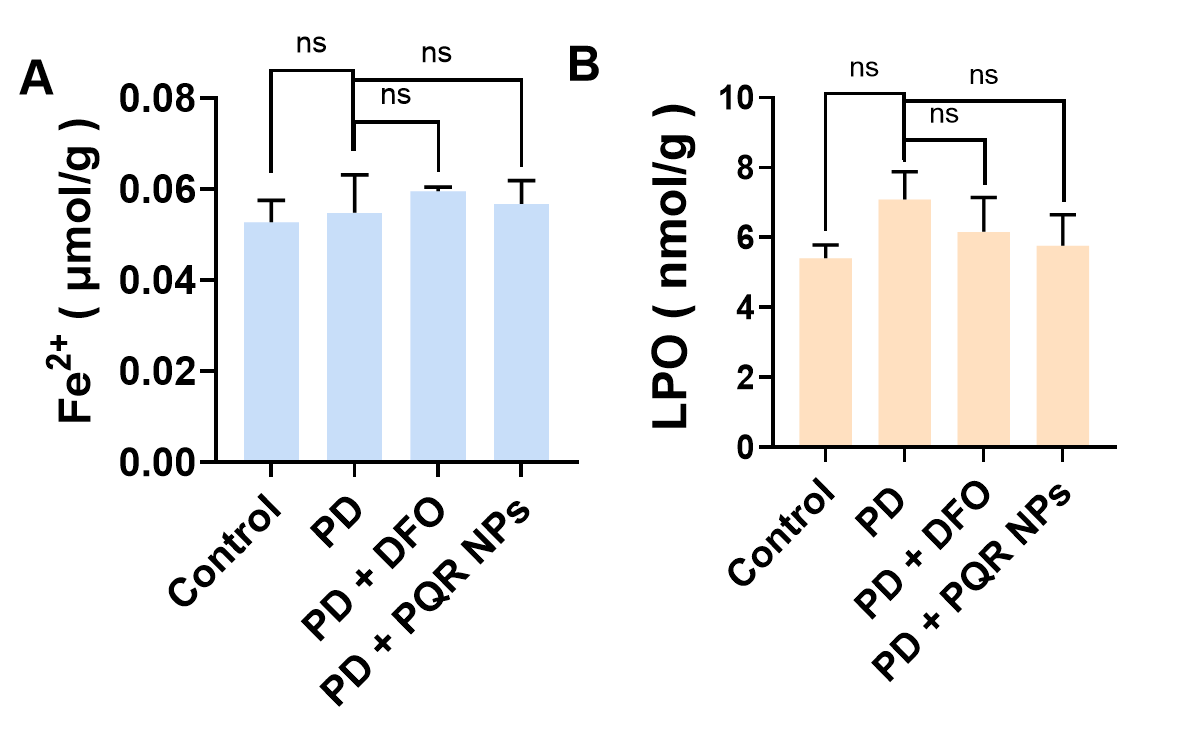


***Figure S48*.** (A) LPO and (B) Fe²⁺ content in the cerebral cortex regions of mice brain among different treatment groups, Data presented as mean ± SD, n=3 for each, *ANOVA test*, ^ns^P > 0.05, *p < 0.05, **p < 0.01, and ***p < 0.001.

***Figure S49*.** Quantitative statistical analysis of IFL staining images of α-Syn from mice treated with different groups. Data presented as mean ± SD, n=3 for each, *ANOVA test*, ***p < 0.001.

***Figure S50.*** Cell viability of PC-12 cell treated with different concentrations of PQR NPs. Data presented as mean ± SD, n=3 for each.


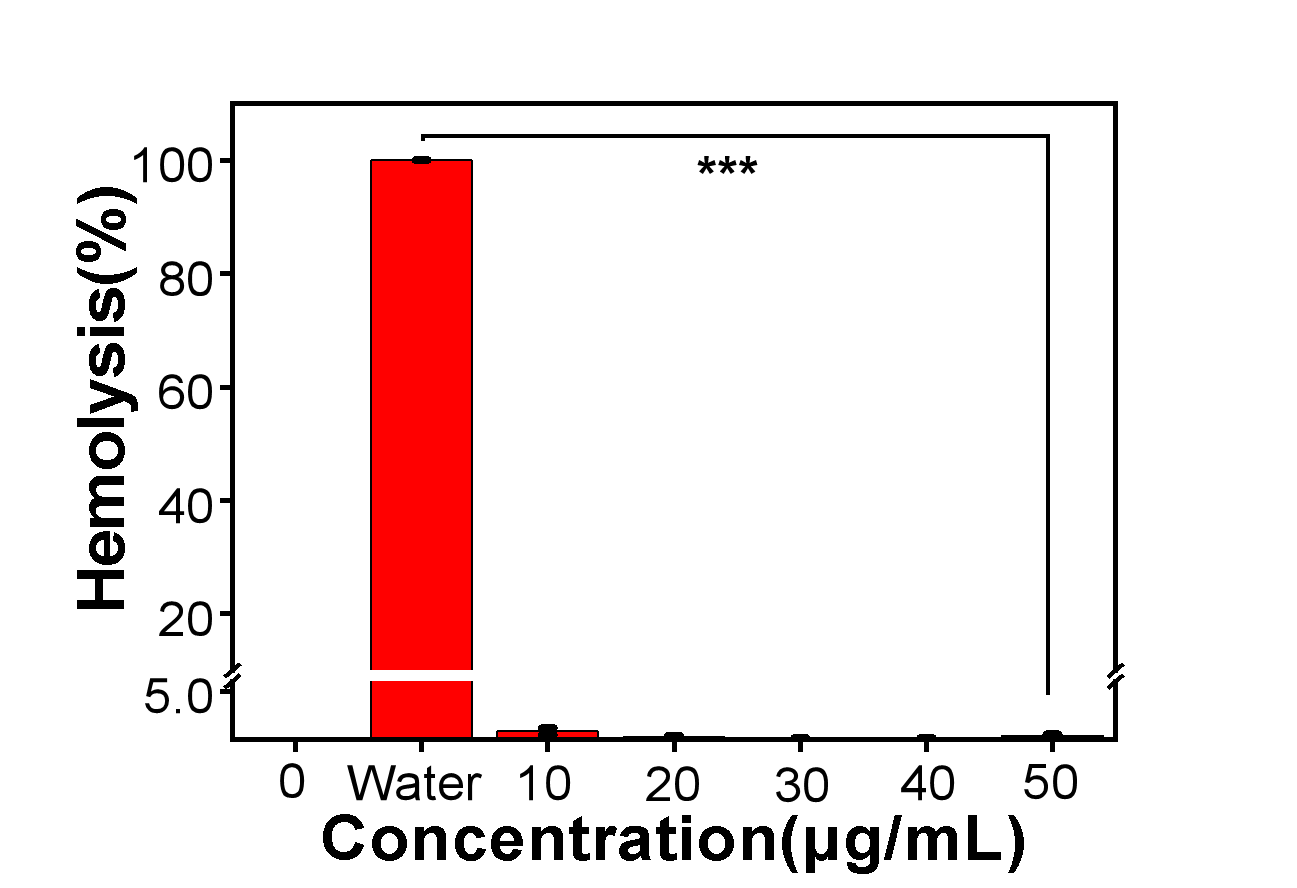


***Figure S51.*** Hemolysis test of different concentrations of PQR NPs. Data presented as mean ± SD, n=3 for each, *Student’s t-test*, *p < 0.05, **p < 0.01, and ***p < 0.001.


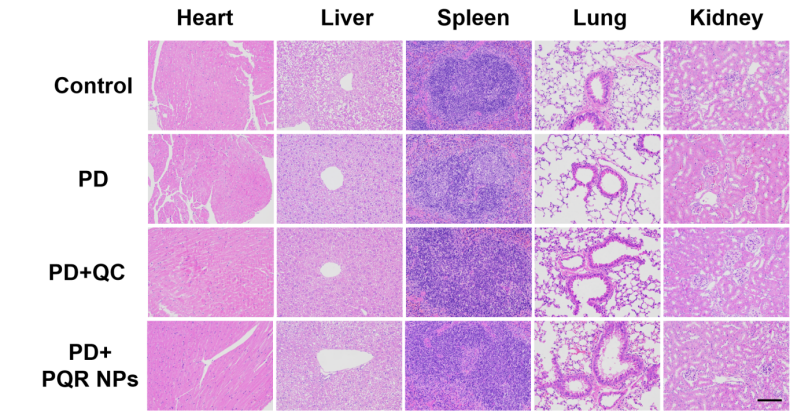


***Figure S52*.** Hematoxylin and Eosin (H&E) staining results of major organs in mice with different treatment (Scale bar: 100 μm).


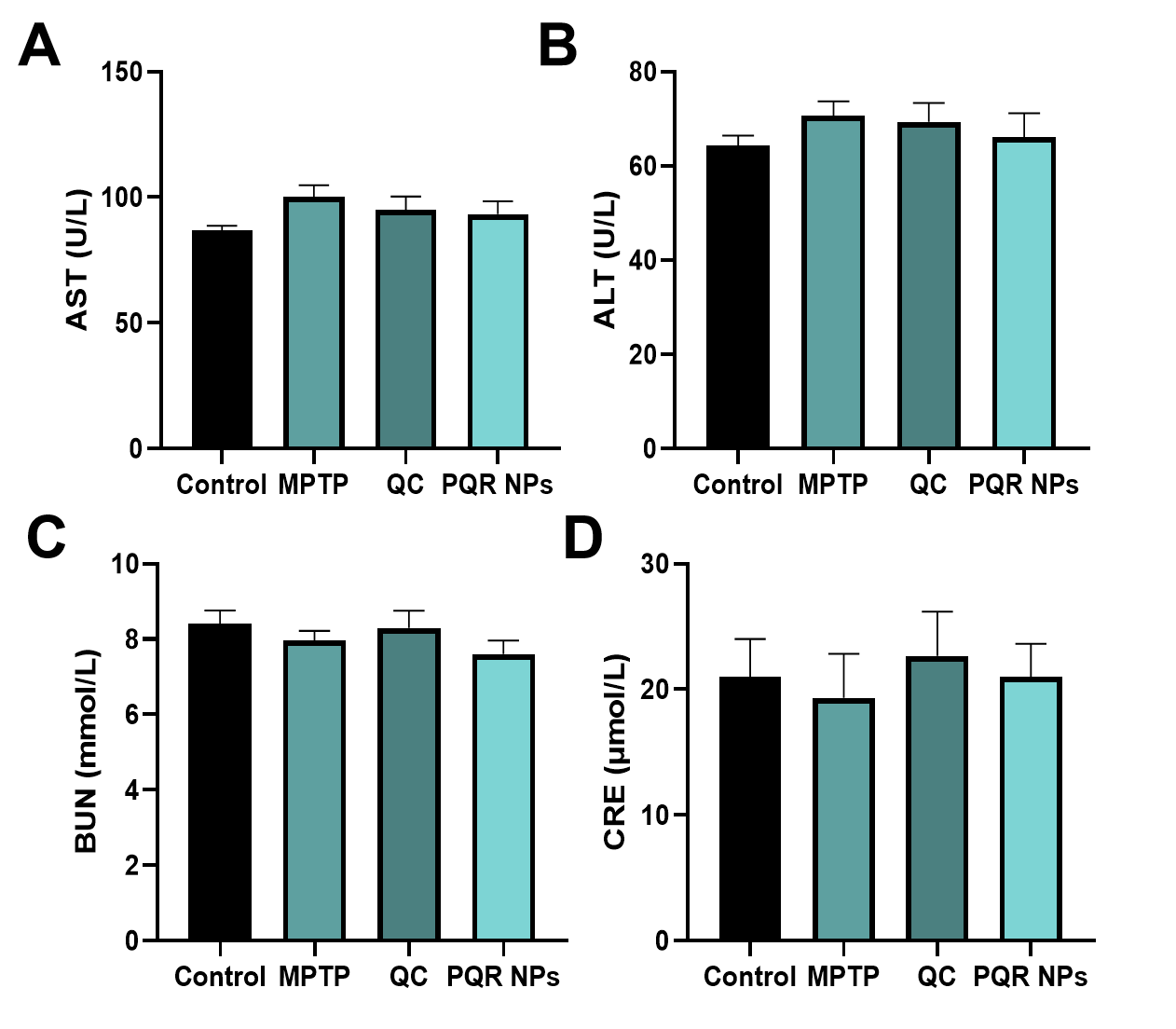


***Figure S53.*** Analysis of serum biochemical index in different treatment groups, (A) Aspartate Aminotransferase (AST), (B) Alanine Aminotransferase (ALT), (C) Blood Urea Nitrogen (BUN), (D) Creatinine (CRE). Data presented as mean ± SD, n=3 for each.

***Table S1.*** Primer pair sequences.

| **Gene name** | **Primer sequences(5’ to 3’)** |
| --- | --- |
| *TNF-α* | GACAAGCCTGTAGCCCATGTGTGTA  CAGCCTTGGCCCTTGAAGA |
| *CD206* | GCCCGGAGTCAGATCACACA  AGTGGCTCAACCCGATGACAG |
| *IL-6* | CTGCAAGAGACTTCCATCCAG  ACAGGTAGAAACGGAACTCCAG |
| *SOD3* | AAAGAGCTCTTGGGAGAGC  CCGTAGAACAAGAAGGCCA |
| *TLR4* | GGCTAGGACTCTGATCATGG  TTAGGAACTACCTCTATGCAGG |

References

1. X. Zhao, T. Liu, X. Han, F. Huo, Y. Yue, C. Yin, *Dyes Pigm.* **2025**, 241, 112890.
